# Tropical refined curve counting from higher genera and lambda classes

**DOI:** 10.1007/s00222-018-0823-z

**Published:** 2018-10-05

**Authors:** Pierrick Bousseau

**Affiliations:** Department of Mathematics, Imperial College London, London, England

**Keywords:** 14T05, 14N10, 14N35

## Abstract

Block and Göttsche have defined a *q*-number refinement of counts of tropical curves in $\mathbb {R}^2$. Under the change of variables $q=e^{iu}$, we show that the result is a generating series of higher genus log Gromov–Witten invariants with insertion of a lambda class. This gives a geometric interpretation of the Block-Göttsche invariants and makes their deformation invariance manifest.

## Introduction

Tropical geometry gives a combinatorial way to approach problems in complex and real algebraic geometry. An early success of this approach is Mikhalkin’s correspondence theorem [34], proved differently and generalized by Nishinou and Siebert [38], between counts of complex algebraic curves in complex toric surfaces and counts with multiplicity of tropical curves in $\mathbb {R}^2$. Our main result, Theorem 1, is an extension to a correspondence between some generating series of higher genus log Gromov–Witten invariants of toric surfaces and counts with *q*-multiplicity of tropical curves in $\mathbb {R}^2$.

Counts of tropical curves in $\mathbb {R}^2$ with *q*-multiplicity were introduced by Block and Göttsche [8]. The usual multiplicity of a tropical curve is defined as a product of integer multiplicities attached to the vertices. Block and Göttsche remarked that one can obtain a refinement by replacing the multiplicity *m* of a vertex by its *q*-analogue

$$\begin{aligned}{}[m]_q {:}{=}\frac{q^{\frac{m}{2}} -q^{-\frac{m}{2}}}{q^{\frac{1}{2}}-q^{-\frac{1}{2}}}= q^{-\frac{m-1}{2}}\left( 1+q+\cdots +q^{m-1}\right) . \end{aligned}$$

The *q*-multiplicity of a tropical curve is then the product of the *q*-multiplicities of the vertices. The count with *q*-multiplicity of tropical curves specializes for $q=1$ to the ordinary count with multiplicity. This definition is done at the tropical level so is combinatorial in nature and its geometric meaning is *a priori* unclear.

Let $\varDelta$ be a balanced collection of vectors in $\mathbb {Z}^2$ and let *n* be a non-negative integer.^1^ This determines a complex toric surface $X_\varDelta$ and a counting problem of virtual dimension zero for complex algebraic curves in $X_\varDelta$ of some genus $g_{\varDelta , n}$, of some class $\beta _\varDelta$, satisfying some tangency conditions with respect to the toric boundary divisor, and passing through *n* points of $X_\varDelta$ in general position. Let $N^{\varDelta , n} \in \mathbb {N}$ be the solution to this counting problem. According to Mikhalkin’s correspondence theorem, $N^{\varDelta , n}$ is a count with multiplicity of tropical curves in $\mathbb {R}^2$, and so it has a Block-Göttsche refinement $N^{\varDelta , n}(q) \in \mathbb {N}[q^{\pm \frac{1}{2}}]$.

For every $g \geqslant g_{\varDelta , n}$, we consider the same counting problem as before—same curve class, same tangency conditions—but for curves of genus *g*. The virtual dimension is now $g-g_{\varDelta , n}$. To obtain a number, we integrate a class of degree $g-g_{\varDelta ,n}$, the lambda class $\lambda _{g-g_{\varDelta ,n}}$, over the virtual fundamental class of a corresponding moduli space of stable log maps. For every $g \geqslant g_{\varDelta , n}$, we get a log Gromov–Witten invariant $N_g^{\varDelta , n} \in \mathbb {Q}$.

### Theorem 1

For every $\varDelta$ balanced collection of vectors in $\mathbb {Z}^2$, and for every non-negative integer *n* such that $g_{\varDelta , n} \geqslant 0$, we have the equality

$$\begin{aligned} \sum _{g \geqslant g_{\varDelta ,n}} N_g^{\varDelta ,n} u^{2g-2+|\varDelta |} =N^{\varDelta , n}(q) \left( (-i) \left( q^{\frac{1}{2}}-q^{-\frac{1}{2}}\right) \right) ^{2g_{\varDelta ,n}-2+|\varDelta |} \end{aligned}$$

of power series in *u* with rational coefficients, where

$$\begin{aligned} q=e^{iu} =\sum _{n \geqslant 0} \frac{(iu)^n}{n!} , \end{aligned}$$

and $|\varDelta |$ is the cardinality of $\varDelta$.

**Remarks**

- According to Theorem 1, the knowledge of the Block-Göttsche invariant $N^{\varDelta ,n }(q)$ is equivalent to the knowledge of the log Gromov–Witten invariants $N^{\varDelta , n}_g$ for all $g \geqslant g_{\varDelta , n}$. This provides a geometric meaning to Block-Göttsche invariants, independent of any choice of tropical limit, making their deformation invariance manifest.
- Given a family $\pi :\mathcal {C}\rightarrow B$ of nodal curves, the Hodge bundle $\mathbb {E}$ is the rank *g* vector bundle over *B* whose fiber over $b \in B$ is the space $H^0(C_b, \omega _{C_b})$ of sections of the dualizing sheaf $\omega _{C_b}$ of the curve $C_b=\pi ^{-1}(b)$. The lambda classes are classically [36] the Chern classes of the Hodge bundle:
  $$\begin{aligned} \lambda _j {:}{=}c_j (\mathbb {E}) . \end{aligned}$$
  The log Gromov–Witten invariants $N_g^{\varDelta , n}$ are defined by an insertion of $(-1)^{g-g_{\varDelta , n}} \lambda _{g-g_{\varDelta , n}}$ to cut down the virtual dimension from $g-g_{\varDelta , n}$ to zero.
- One can interpret Theorem 1 as establishing integrality and positivity properties for higher genus log Gromov–Witten invariants of $X_\varDelta$ with one lambda class inserted.
- The change of variables $q=e^{iu}$ makes the correspondence of Theorem 1 quite non-trivial. In particular, it cannot be reduced to an easy enumerative correspondence. It is essential to have a virtual/non-enumerative count on the Gromov–Witten side: for *g* large enough, most of the contributions to $N_g^{\varDelta , n}$ come from maps with contracted components.
- In Theorem 6, we present a generalization of Theorem 1 where some intersection points with the toric boundary divisor can be fixed.
- One could ask for a generalization of Theorem 1 including descendant log Gromov–Witten invariants, i.e. with insertion of psi classes. In the simplest case of a trivalent vertex with insertion of one psi class, it is possible to reproduce the numerator $q^{\frac{m}{2}}+q^{-\frac{m}{2}}$ of the multiplicity introduced by Göttsche and Schroeter [18] in the context of refined broccoli invariants, in a way similar to how we reproduce the numerator $q^{\frac{m}{2}}-q^{-\frac{m}{2}}$ of the Block-Göttsche multiplicity in Theorem 1. This will be described in some further work.

### Relation with previous and future works

#### *q*-Analogues

It is a general principle in mathematics, going back at least to Heine’s introduction of *q*-hypergeometric series in 1846, that many “classical” notions have a *q*-analogue, recovering the classical one in the limit $q \rightarrow 1$. The Block-Göttsche refinement of the tropical curve counts in $\mathbb {R}^2$ is clearly an example of this principle. In many other examples, it is well known that it is a good idea to write $q=e^{\hbar }$, the limit $q \rightarrow 1$ becoming the limit $\hbar \rightarrow 0$. From this point of view, the change of variable $q=e^{iu}$ in Theorem 1 is maybe not so surprising.

#### Göttsche–Shende refinement by Hirzebruch genus

Whereas the specialization of Block-Göttsche invariants at $q=1$ recovers a count of complex algebraic curves, the specialization $q=-1$ recovers in some cases a count of real algebraic curves in the sense of Welschinger [43]. This strongly suggests a motivic interpretation of the Block-Göttsche invariants and indeed one of the original motivations of Block and Göttsche was the fact that, under some ampleness assumptions, the refined tropical curve counts should coincide with the refined curve counts on toric surfaces defined by Göttsche and Shende [19] in terms of Hirzebruch genera of Hilbert schemes. Using motivic integration, Nicaise, Payne and Schroeter [37] have reduced this conjecture to a conjecture about the motivic measure of a semialgebraic piece of the Hilbert scheme attached to a given tropical curve.

Our approach to the Block-Göttsche refined tropical curve counting is clearly different from the Göttsche–Shende approach: we interpret the refined variable *q* as coming from the resummation of a genus expansion whereas they interpret it as a formal parameter keeping track of the refinement from some Euler characteristic to some Hirzebruch genus.

The Göttsche–Shende refinement makes sense for surfaces more general than toric ones, as do the higher genus log Gromov–Witten invariants with one lambda class inserted. So one might ask if Theorem 1 can be extended to more general surfaces, as a relation between Göttsche–Shende refined invariants and generating series of higher genus log Gromov–Witten invariants. Combining known results about Göttsche–Shende refined invariants [19] and higher genus Gromov–Witten invariants, [11, 33], one can show that it is indeed the case for K3 and abelian surfaces. In particular, Theorem 1 is not an isolated fact but part of a family of similar results. The case of a log Calabi-Yau surface obtained as complement of a smooth anticanonical divisor in a del Pezzo surface, and its relation with, in physics terminology, a worldsheet definition of the refined topological string of local del Pezzo threefolds, will be discussed in a future work.

#### Tropical vertex

Filippini and Stoppa [15] have related refined tropical curve counting to the *q*-version of the tropical vertex of [23], i.e. of the 2-dimensional Kontsevich-Soibelman scattering diagram. Combined with the main result of the present paper, we get an enumerative interpretation of the *q*-version of the tropical vertex. Details will be given in a separate publication [9]. With this enumerative interpretation, it is possible to give an higher genus generalization of the Gross-Hacking-Keel [22] mirror symmetry construction for log Calabi-Yau surfaces [10].

Using the connection with the *q*-version of the tropical vertex, Filippini and Stoppa [15] have related refined tropical curve counting to refined Donaldson–Thomas theory of quivers. This story was the initial motivation for the work eventually leading to the present paper. Applications of the present paper in this context will be discussed elsewhere.

#### MNOP

The change of variables $q=e^{iu}$ is reminiscent of the MNOP Gromov–Witten/Donaldson–Thomas (DT) correspondence on threefold [31, 32]. The log Gromov–Witten invariants $N_g^{\varDelta , n}$ can be rewritten as $\mathbb {C}^*$-equivariant log Gromov–Witten invariants of the threefold $X_\varDelta \times \mathbb {C}$, where $\mathbb {C}^*$ acts by scaling on $\mathbb {C}$, see Lemma 7 of [33]. If a log DT theory and a log MNOP correspondence were developed, this would predict that the generating series of $N_g^{\varDelta , n}$ is a rational function in $q=e^{iu}$, which is indeed true by Theorem 1. But it would not be enough to imply Theorem 1 because the relation between log DT invariants and Block-Göttsche invariants is *a priori* unclear. In fact, the Göttsche–Shende conjecture and the result of Filippini and Stoppa suggest that Block-Göttsche invariants are refined DT invariants whereas the MNOP correspondence involves unrefined DT invariants. This topic will be discussed in more details elsewhere.

#### BPS integrality

When the log Gromov–Witten invariants of $X_\varDelta \times \mathbb {C}$ coincide with ordinary Gromov–Witten invariants of $X_\varDelta \times \mathbb {C}$, which is probably the case if $|v|=1$ for every $v \in \varDelta$ and if the toric boundary divisor of $X_\varDelta$ is positive enough, then the integrality implied by Theorem 1 coincides with the BPS integrality predicted by Pandharipande in [41], and proved via symplectic methods by Zinger in [44], for generating series of Gromov–Witten invariants of a threefold and of curve class intersecting positively the anticanonical divisor.

#### Mikhalkin refined real count

Mikhalkin has given in [35] an interpretation of some particular Block-Göttsche invariants in terms of counts of real curves. We do not understand the relation with our approach in terms of higher genus log Gromov–Witten invariants. We merely remark that both for us and for Mikhalkin, it is the numerator of the Block-Göttsche multiplicities which appears naturally.

#### Parker theory of exploded manifolds

This paper owes a great intellectual debt towards the paper [42] of Brett Parker, where a correspondence theorem between tropical curves in $\mathbb {R}^3$ and some generating series of curve counts in exploded versions of toric threefold is proved. Indeed, a conjectural version of Theorem 1 was known to the author around April 2016^2^ but it was only after the appearance of [42] in August 2016 that it became clear that this result should be provable with existing technology. In particular, the idea to reduce the amount of explicit computations by exploiting the consistency of some gluing formula (see Sect. 8) follows [42]. In the present paper, we use the theory of log Gromov–Witten invariants because of the algebraic bias of the author, but it should be possible to write a version in the language of exploded manifolds.

### Plan of the paper

In Sect. 2, we fix our notations and we describe precisely the objects involved in the formulation of Theorem 1. In Sect. 3, we review some gluing and vanishing properties of the lambda classes.

The next five Sections form the proof of Theorem 1.

The first step of the proof, described in Sect. 4, is an application of the decomposition formula of Abramovich, Chen, Gross, Siebert [3] to the toric degeneration of Nishinou, Siebert [38]. This gives a way to write our log Gromov–Witten invariants as a sum of contributions indexed by tropical curves.

In the second step of the proof, described in Sects. 6 and 7, we prove a gluing formula which gives a way to write the contribution of a tropical curve as a product of contributions of its vertices. Here, gluing and vanishing properties of the lambda classes reviewed in Sect. 3, combined with a structure result for non-torically transverse stable log maps proved in Sect. 5, play an essential role. In particular, we only have to glue torically transverse stable log maps and we don’t need to worry about the technical issues making the general gluing formula in log Gromov–Witten theory difficult (see Abramovich, Chen, Gross, Siebert [4]).

After the decomposition and gluing steps, what remains to do is to compute the contribution to the log Gromov–Witten invariants of a tropical curve with a single trivalent vertex. The third and final step of the proof of Theorem 1, carried out in Sect. 8, is the explicit evaluation of this vertex contribution. Consistency of the gluing formula leads to non-trivial relations between these vertex contributions, which enable us to reduce the problem to particularly simple vertices. The contribution of these simple vertices is computed explicitly by reduction to Hodge integrals previously computed by Bryan and Pandharipande [12] and this ends the proof of Theorem 1.

In Appendix A, we present for the sake of concreteness an explicit example.

## Precise statement of the main result

### Toric geometry

Let $\varDelta$ be a balanced collection of vectors in $\mathbb {Z}^2$, i.e. a finite collection of vectors in $\mathbb {Z}^2 - \{0\}$ summing to zero.^3^ Let $|\varDelta |$ be the cardinality of $\varDelta$. For $v \in \mathbb {Z}^2-\{0\}$, let |*v*| the divisibility of *v* in $\mathbb {Z}^2$, i.e. the largest positive integer *k* such that we can write $v=kv'$ with $v' \in \mathbb {Z}^2$. Then the balanced collection $\varDelta$ defines the following data by standard toric geometry.

- A projective^4^ toric surface $X_\varDelta$ over $\mathbb {C}$, whose fan has rays $\mathbb {R}_{\geqslant 0}v$ generated by the vectors $v \in \mathbb {Z}^2-\{0\}$ contained in $\varDelta$. We denote $\partial X_\varDelta$ the toric boundary divisor of $X_\varDelta$.
- A curve class $\beta _\varDelta$ on $X_\varDelta$, whose polytope is dual to $\varDelta$. If $\rho$ is a ray in the fan of $X_\varDelta$, we write $D_\rho$ for the prime toric divsisor of $X_\varDelta$ dual to $\rho$ and $\varDelta _\rho$ the set of elements $v \in \varDelta$ such that $\mathbb {R}_{\geqslant 0} v=\rho$. Then we have
  $$\begin{aligned} \beta _\varDelta .D_{\rho } =\sum _{v \in \varDelta _\rho } |v| , \end{aligned}$$
  and these intersection numbers uniquely determine $\beta _\varDelta$. The total intersection number of $\beta _\varDelta$ with the toric boundary divisor $\partial X_\varDelta$ is given by
  $$\begin{aligned} \beta _\varDelta .(-K_{X_\varDelta })=\sum _{v \in \varDelta } |v|. \end{aligned}$$
- Tangency conditions for curves of class $\beta _\varDelta$ with respect to the toric boundary divisor of $X_\varDelta$. We say that a curve *C* is of type $\varDelta$ if it is of class $\beta _\varDelta$ and if for every ray $\rho$ in the fan of $X_\varDelta$, the curve *C* intersects $D_\rho$ in $|\varDelta _\rho |$ points with multiplicities |*v*|, $v \in \varDelta _\rho$. Similarly, we have a notion of stable log map of type $\varDelta$.
- An asymptotic form for a parametrized tropical curve $h :\varGamma \rightarrow \mathbb {R}^2$ in $\mathbb {R}^2$. We say that a parametrized tropical curve in $\mathbb {R}^2$ is of type $\varDelta$ if it has $|\varDelta |$ unbounded edges, with directions *v* and with weights |*v*|, $v \in \varDelta$.

### Log Gromov–Witten invariants

The moduli space of *n*-pointed genus *g* stable maps to $X_\varDelta$ of class $\beta _\varDelta$ intersecting properly the toric boundary divisor $\partial X_\varDelta$ with tangency conditions prescribed by $\varDelta$ is not proper: a limit of curves intersecting $\partial X_\varDelta$ properly does not necessarily intersect $\partial X_\varDelta$ properly. A nice compactification of this space is obtained by considering stable log maps. The idea is to allow maps intersecting $\partial X_\varDelta$ non-properly, but to remember some additional information under the form of log structures, which give a way to make sense of tangency conditions even for non-proper intersections. The theory of stable log maps has been developed by Gross and Siebert [24], and Abramovich and Chen [2, 14]. By stable log maps, we always mean basic stable log maps in the sense of [24]. We refer to Kato [26] for elementary notions of log geometry.

We consider the toric divisorial log structure on $X_\varDelta$ and use it to view $X_\varDelta$ as a log scheme. Let ${\overline{M}}_{g,n, \varDelta }$ be the moduli space of *n*-pointed genus *g* stable log maps to $X_\varDelta$ of type $\varDelta$. By *n*-pointed, we mean that the source curves are equipped with *n* marked points *in addition* to the marked points keeping track of the tangency conditions with respect to the toric boundary divisor. We consider that the latter are notationally already included in $\varDelta$.

By the work of Gross, Siebert [24] and Abramovich, Chen [2, 14], ${\overline{M}}_{g,n, \varDelta }$ is a proper Deligne-Mumford stack^5^ of virtual dimension

$$\begin{aligned} {\text {vdim}}\,{\overline{M}}_{g,n,\varDelta } \!=\! g-1+n+\beta _\varDelta .(-K_{X_\varDelta })- \sum _{v \in \varDelta } (|v|-1)\!=\!g-1+n+|\varDelta | , \end{aligned}$$

and it admits a virtual fundamental class

$$\begin{aligned}{}[\overline{M}_{g,n,\varDelta }]^{\mathrm {virt}} \in A_{{\text {vdim}}\,{\overline{M}}_{g,n,\varDelta }} ({\overline{M}}_{g,n,\varDelta },\mathbb {Q}). \end{aligned}$$

The problem of counting *n*-pointed genus *g* curves passing though *n* fixed points has virtual dimension zero if

$$\begin{aligned} {\text {vdim}}\,{\overline{M}}_{g,n,\varDelta }=2n , \end{aligned}$$

i.e. if the genus *g* is equal to

$$\begin{aligned} g_{\varDelta ,n} {:}{=}n+1-|\varDelta | . \end{aligned}$$

In this case, the corresponding count of curves is given by

$$\begin{aligned} N^{\varDelta ,n} {:}{=}\left\langle \tau _0(\mathrm {pt})^n \right\rangle _{g_{\varDelta ,n},n,\varDelta } {:}{=}\int _{[{\overline{M}}_{g_{\varDelta ,n},n,\varDelta }]^{\mathrm {virt}}} \prod _{j=1}^n {\text {ev}}_j^{*}(\mathrm {pt}), \end{aligned}$$

where $\mathrm {pt} \in A^2(X_\varDelta )$ is the class of a point and ${\text {ev}}_j$ is the evaluation map at the *j*-th marked point.

According to Mandel and Ruddat [30], Mikhalkin’s correspondence theorem can be reformulated in terms of these log Gromov–Witten invariants. Our refinement of the correspondence theorem will involve curves of genus $g \geqslant g_{\varDelta , n}$.

For $g > g_{\varDelta , n}$, inserting *n* points is no longer enough to cut down the virtual dimension to zero. The idea is to consider the Hodge bundle $\mathbb {E}$ over ${\overline{M}}_{g,n,\varDelta }$. If $\pi :\mathcal {C}\rightarrow {\overline{M}}_{g,n,\varDelta }$ is the universal curve, of relative dualizing^6^ sheaf $\omega _\pi$, then

$$\begin{aligned} \mathbb {E}\,{:}{=}\,\pi _* \omega _\pi \end{aligned}$$

is a rank *g* vector bundle over ${\overline{M}}_{g,n,\varDelta }$. The Chern classes of the Hodge bundle are classically [36] called the lambda classes and denoted as

$$\begin{aligned} \lambda _j \,{:}{=}\,c_j(\mathbb {E}) , \end{aligned}$$

for $j=0,\ldots ,g$. Because the virtual dimension of ${\overline{M}}_{g,n,\varDelta }$ is given by

$$\begin{aligned} {\text {vdim}}\,{\overline{M}}_{g,n,\varDelta }=g-g_{\varDelta ,n} +2n , \end{aligned}$$

inserting the lambda class $\lambda _{g-g_{\varDelta , n}}$ and *n* points will cut down the virtual dimension to zero, so it is natural to consider the log Gromov–Witten invariants with one lambda class inserted

$$\begin{aligned} N^{\varDelta ,n}_g&{:}{=}&\langle (-1)^{g-g_{\varDelta ,n}} \lambda _{g-g_{\varDelta ,n}} \tau _0(\mathrm {pt})^n \rangle _{g,n,\varDelta }\\&{:}{=}&\int _{[{\overline{M}}_{g,n,\varDelta }]^{\mathrm {virt}}} (-1)^{g-g_{\varDelta ,n}} \lambda _{g-g_{\varDelta ,n}} \prod _{j=1}^n \text {ev}_j^{*}(\mathrm {pt}). \end{aligned}$$

Our refined correspondence result, Theorem 5, gives an interpretation of the generating series of these invariants in terms of refined tropical curve counting.

### Tropical curves

We refer to Mikhalkin [34], Nishinou, Siebert [38], Mandel, Ruddat [30], and Abramovich, Chen, Gross, Siebert [3] for basics on tropical curves. Each of these references uses a slightly different notion of parametrized tropical curve. We will use a variant of [3], Definition 2.5.3, because it is the one which is the most directly related to log geometry. It is easy to go from one to the other.

For us, a graph $\varGamma$ has a finite set $V(\varGamma )$ of vertices, a finite set $E_f(\varGamma )$ of bounded edges connecting pairs of vertices and a finite set $E_\infty (\varGamma )$ of legs attached to vertices that we view as unbounded edges. By edge, we refer to a bounded or unbounded edge. We will always consider connected graphs.

A parametrized tropical curve $h :\varGamma \rightarrow \mathbb {R}^2$ is the following data:

- A non-negative integer *g*(*V*) for each vertex *V*, called the genus of *V*.
- A bijection of the set $E_\infty (\varGamma )$ of unbounded edges with
  $$\begin{aligned} \{ 1, \ldots , |E_\infty (\varGamma )| \} , \end{aligned}$$
  where $|E_\infty (\varGamma )|$ is the cardinality of $E_\infty (\varGamma )$.
- A vector $v_{V,E} \in \mathbb {Z}^2$ for every vertex *V* and *E* an edge adjacent to *V*. If $v_{V,E}$ is not zero, the divisibility $|v_{V,E}|$ of $v_{V,E}$ in $\mathbb {Z}^2$ is called the weight of *E* and is denoted *w*(*E*). We require that $v_{V,E} \ne 0$ if *E* is unbounded and that for every vertex *V*, the following balancing condition is satisfied:
  $$\begin{aligned} \sum _E v_{V,E} =0 , \end{aligned}$$
  where the sum is over the edges *E* adjacent to *V*. In particular, the collection $\varDelta _V$ of non-zero vectors $v_{\varDelta ,E}$ for *E* adjacent to *V* is a balanced collection as in Sect. 2.1.
- A non-negative real number $\ell (E)$ for every bounded edge of *E*, called the length of *E*.
- A proper map $h :\varGamma \rightarrow \mathbb {R}^2$ such that
  - If *E* is a bounded edge connecting the vertices $V_1$ and $V_2$, then *h* maps *E* affine linearly on the line segment connecting $h(V_1)$ and $h(V_2)$, and $h(V_2)-h(V_1) = \ell (E)v_{V_1,E}$.
  - If *E* is an unbounded edge of vertex *V*, then *h* maps *E* affine linearly to the ray $h(V)+\mathbb {R}_{\geqslant 0} v_{V,E}$.

The genus $g_h$ of a parametrized tropical curve $h :\varGamma \rightarrow \mathbb {R}^2$ is defined by

$$\begin{aligned} g_h {:}{=}g_\varGamma + \sum _{V \in V(\varGamma )} g(V) , \end{aligned}$$

where $g_\varGamma$ is the genus of the graph $\varGamma$.

We fix $\varDelta$ a balanced collection of vectors in $\mathbb {Z}^2$, as in Sect. 2.1, and we fix a bijection of $\varDelta$ with $\{1, \ldots , |\varDelta | \}$. We say that a parametrized tropical curve $h :\varGamma \rightarrow \mathbb {R}^2$ is of type $\varDelta$ if there exists a bijection between $\varDelta$ and $\{ v_{V,E} \}_{E \in E_\infty (\varGamma )}$ compatible with the fixed bijections to

$$\begin{aligned} \{ 1,\ldots , |\varDelta | \} = \{ 1, \ldots , |E_\infty (\varGamma )|\} . \end{aligned}$$

Remark that

$$\begin{aligned} \sum _{E \in E_\infty (\varGamma )} v_{V,E}=0 \end{aligned}$$

by the balancing condition.

We say that a parametrized tropical curve $h :\varGamma \rightarrow \mathbb {R}^2$ is *n*-pointed if we have chosen a distribution of the labels $1, \ldots , n$ over the vertices of $\varGamma$, a vertex having the possibility to have several labels. Vertices without any label are said to be unpointed whereas those with labels are said to be pointed. For $j=1, \ldots , n$, let $V_j$ be the pointed vertex having the label *j*. Let $p=(p_1, \ldots , p_n)$ be a configuration of *n* points in $\mathbb {R}^2$. We say that a *n*-pointed parametrized tropical curve $h :\varGamma \rightarrow \mathbb {R}^2$ passes through *p* if $h(V_j)=p_j$ for every $j=1, \ldots , n$. We say that a *n*-pointed parametrized tropical curve $h :\varGamma \rightarrow \mathbb {R}^2$ passing through *p* is rigid if it is not contained in a non-trivial family of *n*-pointed parametrized tropical curves passing through *p* of the same combinatorial type.

#### Proposition 2

For every balanced collection $\varDelta$ of vectors in $\mathbb {Z}^2$, and *n* a non-negative integer such that $g_{\varDelta , n} \geqslant 0$, there exists an open dense subset $U_{\varDelta , n}$ of $(\mathbb {R}^2)^n$ such that if $p=(p_1, \ldots , p_n) \in U_{\varDelta , n}$ then $p_j \ne p_k$ for $j \ne k$ and if $h :\varGamma \rightarrow \mathbb {R}^2$ is a rigid^7^*n*-pointed parametrized tropical curve of genus $g \leqslant g_{\varDelta , n}$ and of type $\varDelta$ passing through *p*, then

- $$g=g_{\varDelta ,n}$$.
- We have $g(V)=0$ for every vertex *V* of $\varGamma$. In particular, the graph $\varGamma$ has genus $g_{\varDelta , n}$.
- Images by *h* of distinct vertices are distinct.
- No edge is contracted to a point.
- Images by *h* of two distinct edges intersect in at most one point.
- Unpointed vertices are trivalent.
- Pointed vertices are bivalent.

#### Proof

This is essentially Proposition 4.11 of Mikhalkin [34], which itself is essentially some counting of dimensions. In [34], there is no genus attached to the vertices but if we have a parametrized tropical curve of genus $g \leqslant g_{\varDelta ,n }$ with some vertices of non-zero genus, the underlying graph has genus strictly less than *g* and so strictly less than $g_{\varDelta , n}$, which is impossible by Proposition 4.11 of [34] for *p* general enough. $\square$

#### Proposition 3

If $p \in U_{\varDelta ,n}$, then the set $T_{\varDelta , p}$ of rigid *n*-pointed genus $g_{\varDelta , n}$ parametrized tropical curves $h :\varGamma \rightarrow \mathbb {R}^2$ of type $\varDelta$ passing through *p* is finite.

#### Proof

This is Proposition 4.13 if Mikhalkin [34]: there are finitely many possible combinatorial types for a parametrized tropical curve as in Proposition 2, and for a fixed combinatorial type, the set of such tropical curves passing through *p* is a zero dimensional intersection of a linear subspace with an open convex polyhedron, so is a point. $\square$

#### Lemma 4

Let $h :\varGamma \rightarrow \mathbb {R}^2$ be a parametrized tropical curve in $T_{\varDelta , p}$. Then $\varGamma$ has $2g_{\varDelta , n}-2+|\varDelta |$ trivalent vertices.

#### Proof

By definition of $T_{\varDelta , p}$, the graph $\varGamma$ is of genus $g_{\varDelta ,n}$ and its vertices are either trivalent or bivalent. Replacing the two edges adjacent to each bivalent vertex by a unique edge, we obtain a trivalent graph ${\hat{\varGamma }}$ with the same genus and the same number of unbounded edges as $\varGamma$. Let $|V({\hat{\varGamma }})|$ be the number of vertices of ${\hat{\varGamma }}$ and let $|E_{f}({\hat{\varGamma }})|$ be the number of bounded edges of ${\hat{\varGamma }}$. A count of half-edges using that ${\hat{\varGamma }}$ is trivalent gives

$$\begin{aligned} 3 |V({\hat{\varGamma }})| = 2 |E_f({\hat{\varGamma }})| + |\varDelta | . \end{aligned}$$

By definition of the genus, we have

$$\begin{aligned} 1-g_{\varDelta ,n} =|V({\hat{\varGamma }})| - |E_f({\hat{\varGamma }})|. \end{aligned}$$

Eliminating $|E_f({\hat{\varGamma }})|$ from the two previous equalities gives the desired formula and so finishes the proof of Lemma 4. $\square$

For $h :\varGamma \rightarrow \mathbb {R}^2$ a parametrized tropical curve in $\mathbb {R}^2$ and *V* a trivalent vertex of adjacent edges $E_1$, $E_2$ and $E_3$, the multiplicity of *V* is the integer defined by

$$\begin{aligned} m(V) {:}{=}|\det (v_{V,E_1}, v_{V,E_2})| . \end{aligned}$$

Thanks to the balancing condition

$$\begin{aligned} v_{V,E_1}+v_{V,E_2}+v_{V,E_3}=0 , \end{aligned}$$

we also have

$$\begin{aligned} m(V)= |\det (v_{V,E_2}, v_{V,E_3})| =| \det (v_{V,E_3}, v_{V,E_1})|. \end{aligned}$$

For $(h :\varGamma \rightarrow \mathbb {R}^2) \in T_{\varDelta ,p}$, the multiplicity of *h* is defined by

$$\begin{aligned} m_h {:}{=}\prod _{V \in V^{(3)}(\varGamma )} m(V) , \end{aligned}$$

where the product is over the trivalent, i.e. unpointed, vertices of $\varGamma$.

We denote $N^{\varDelta ,p}_{\mathrm {trop}}$ the count with multiplicity of *n*-pointed genus $g_{\varDelta , n}$ parametrized tropical curves of type $\varDelta$ passing through *p*, i.e.

$$\begin{aligned} N^{\varDelta , p}_{\mathrm {trop}} {:}{=}\sum _{h \in T_{\varDelta , p}} m_h . \end{aligned}$$

This tropical count with multiplicity has a natural refinement, first suggested by Block and Göttsche [8]. We can replace the integer valued multiplicity $m_h$ of a parametrized tropical curve $h :\varGamma \rightarrow \mathbb {R}^2$ by the $\mathbb {N}[q^{\pm \frac{1}{2}}]$-valued multiplicity

$$\begin{aligned} m_h(q) {:}{=}\prod _{V \in V^{(3)}(\varGamma )} \frac{q^{\frac{m(V)}{2}} -q^{-\frac{m(V)}{2}}}{q^{\frac{1}{2}}-q^{-\frac{1}{2}}} = \prod _{V \in V^{(3)}(\varGamma )} \left( \sum _{j=0}^{m(V)-1} q^{-\frac{m(V)-1}{2}+j} \right) , \end{aligned}$$

where the product is taken over the trivalent vertices of $\varGamma$. The specialization $q=1$ recovers the usual multiplicity:

$$\begin{aligned} m_h(1)=m_h . \end{aligned}$$

Counting the parametrized tropical curves in $T_{\varDelta , p}$ as above but with *q*-multiplicities, we obtain a refined tropical count

$$\begin{aligned} N^{\varDelta ,p}_{\mathrm {trop}} (q) {:}{=}\sum _{h \in T_{\varDelta , p}} m_h (q) \in \mathbb {N}[q^{\pm \frac{1}{2}}] , \end{aligned}$$

which specializes to the tropical count $N^{\varDelta , p}_{\mathrm {trop}}$ at $q=1$:

$$\begin{aligned} N^{\varDelta , p}_{\mathrm {trop}} (1)=N^{\varDelta , p}_{\mathrm {trop}} . \end{aligned}$$

### Unrefined correspondence theorem

Let $\varDelta$ be a balanced collection of vectors in $\mathbb {Z}^2$, as in Sect. 2.1, and let *n* be a non-negative integer and $p \in U_{\varDelta ,n}$. Then we have some log Gromov–Witten count $N^{\varDelta , n}$ of *n*-pointed genus $g_{\varDelta ,n}$ curves of type $\varDelta$ passing through *n* points in the toric surface $X_\varDelta$ (see Sect. 2.2), and we have some count with multiplicity $N^{\varDelta ,n}_{\mathrm {trop}}$ of *n*-pointed genus $g_{\varDelta , n}$ tropical curves of type $\varDelta$ passing through *n* points $p=(p_1, \ldots , p_n)$ in $\mathbb {R}^2$ (see Sect. 2.3). The (unrefined) correspondence theorem then takes the simple form

$$\begin{aligned} N^{\varDelta ,n}=N^{\varDelta ,p}_{\mathrm {trop}}. \end{aligned}$$

The result proved by Mikhalkin [34] and generalized by Nishinou, Siebert [38] is an equality between the tropical count $N^{\varDelta ,n}_{\mathrm {trop}}$ and an enumerative count of algebraic curves. The fact that this enumerative count coincides with the log Gromov–Witten count $N^{\varDelta , n}$ is proved by Mandel and Ruddat in [30].

### Refined correspondence theorem

The Block-Göttsche refinement from $N^{\varDelta , p}$ to $N^{\varDelta , p}(q)$, reviewed in Sect. 2.3, is done at the tropical level so is combinatorial in nature and its geometric meaning is a priori unclear.

The main result of the present paper is a new non-tropical interpretation of Block-Göttsche invariants in terms of the higher genus log Gromov–Witten invariants with one lambda class inserted $N_{\varDelta , n}^g$ that we introduced in Sect. 2.2. In particular, this geometric interpretation is independent of any tropical limit and makes the tropical deformation invariance of Block-Göttsche invariants manifest.

More precisely, we prove a refined correspondence theorem, already stated as Theorem 1 in the Introduction.

#### Theorem 5

For every $\varDelta$ balanced collection of vectors in $\mathbb {Z}^2$, for every non-negative integer *n* such that $g_{\varDelta , n} \geqslant 0$, and for every $p \in U_{\varDelta , n}$, we have the equality

$$\begin{aligned} \sum _{g \geqslant g_{\varDelta ,n}} N_g^{\varDelta ,n} u^{2g-2+|\varDelta |} =N^{\varDelta ,p}_{\mathrm {trop}}(q) \left( (-i) (q^{\frac{1}{2}}-q^{-\frac{1}{2}}) \right) ^{2g_{\varDelta ,n}-2+|\varDelta |} \end{aligned}$$

of power series in *u* with rational coefficients, where

$$\begin{aligned} q=e^{iu} =\sum _{n \geqslant 0} \frac{(iu)^n}{n!} . \end{aligned}$$

**Remarks**

- The change of variables $q=e^{iu}$ makes the above correspondence quite non-trivial. In particular, in contrast to its unrefined version, it cannot be reduced to a finite to one enumerative correspondence. It is essential to have a virtual/non-enumerative count on the Gromov–Witten side: for *g* large enough, most of the contributions to $N_g^{\varDelta , n}$ come from maps with contracted components.
- The refined tropical count has the symmetry $N^{\varDelta , n}_{\mathrm {trop}}(q)=N^{\varDelta , n}_{\mathrm {trop}}(q^{-1})$ and so, after the change of variables $q=e^{iu}$, is a even power series in *u*. In particular, as
  $$\begin{aligned} (-i) (q^{\frac{1}{2}}-q^{-\frac{1}{2}}) \in u \mathbb {Q}[\![u^2]\!] , \end{aligned}$$
  the tropical side of Theorem 5 lies in
  $$\begin{aligned} u^{2g_{\varDelta ,n}-2+|\varDelta |} \mathbb {Q}[\![u^2]\!], \end{aligned}$$
  as does the Gromov–Witten side. Taking the leading order terms on both sides in the limit $u \rightarrow 0$, $q \rightarrow 1$, we recover the unrefined correspondence theorem $N^{\varDelta ,n} =N^{\varDelta , p}_{\mathrm {trop}}$.
- By Lemma 4, we know that $2g_{\varDelta ,n }-2+|\varDelta |$ is the number of trivalent vertices of a parametrized tropical curve in $T_{\varDelta , p}$. In particular, the tropical side of Theorem 5 can be obtained directly by considering only the numerators of the Block-Göttsche multiplicities, i.e. Theorem 5 can be rewritten
  $$\begin{aligned} \sum _{g\geqslant g_{\varDelta ,n}} N^{\varDelta ,n}_g u^{2g-2+|\varDelta |}= \sum _{h \in T_{\varDelta , p}} \prod _V (-i)\left( q^{\frac{m(V)}{2}} -q^{-\frac{m(V)}{2}}\right) , \end{aligned}$$
  where $$q=e^{iu}$$.

### Fixing points on the toric boundary

It is possible to generalize Theorem 5 by fixing the position of some of the intersection points with the toric boundary divisor. Let $\varDelta ^F$ be a subset of $\varDelta$ and let

$$\begin{aligned} \mathrm {ev}_{\varDelta ^F} :{\overline{M}}_{g,n,\varDelta } \rightarrow (\partial X_\varDelta )^{|\varDelta ^F|} \end{aligned}$$

be the evaluation map at the intersection points with the toric boundary divisor $\partial X_\varDelta$ indexed by the elements of $\varDelta ^F$.

The problem of counting *n*-pointed genus *g* curves of type $\varDelta$ passing through *n* given points of $X_\varDelta$ and with fixed position of the intersection points with $\partial X_\varDelta$ indexed by $\varDelta ^F$, has virtual dimension zero if the genus is equal to

$$\begin{aligned} g_{\varDelta ,n}^{\varDelta ^F} {:}{=}n+1- |\varDelta | + |\varDelta ^F| . \end{aligned}$$

For every $g \geqslant g_{\varDelta ,n}^{\varDelta ^F}$, we define the invariants

$$\begin{aligned} N^{\varDelta ,n}_{g,\varDelta ^F} {:}{=}\int _{[{\overline{M}}_{g,n,\varDelta }]^{virt}} (-1)^{g-g_{\varDelta ,n}^{\varDelta ^F}} \lambda _{g-g_{\varDelta ,n}^{\varDelta ^F}} \text {ev}_{\varDelta ^F}^{*} (r^{|\varDelta ^F|}) \prod _{j=1}^n \text {ev}_j^{*}(\mathrm {pt}) , \end{aligned}$$

where $r \in A^1(\partial X_\varDelta )$ is the class of a point on $\partial X_\varDelta$.

We can consider the corresponding tropical problem. Fix a generic configuration $x=(x_v)_{v \in \varDelta ^F}$ of points in $\mathbb {R}^2$ and say that a tropical curve of type $\varDelta$ is of type $(\varDelta , \varDelta ^F)$ if the unbounded edges in correspondence with $\varDelta ^F$ asymptotically coincide with the half-lines $x_v + \mathbb {R}_{\geqslant 0} v$, $v \in \varDelta ^F$.

We define a refined tropical count

$$\begin{aligned} N^{\varDelta , p, x}_{\mathrm {trop},\varDelta ^F}(q) \in \mathbb {N}[q^{\pm \frac{1}{2}}] , \end{aligned}$$

by counting with *q*-multiplicity the tropical curves of genus $g_{\varDelta ,n}^{\varDelta ^F}$ and of type $(\varDelta , \varDelta ^F)$ passing through a generic configuration $p=(p_1, \ldots , p_n)$ of *n* points in $\mathbb {R}^2$.

The following result is the generalization of Theorem 5 to the case of non-empty $\varDelta ^F$.

#### Theorem 6

For every $\varDelta$ balanced collection of vectors in $\mathbb {Z}^2$, for every $\varDelta ^F$ subset of $\varDelta$ and for every *n* non-negative integer such that $g_{\varDelta ,n}^{\varDelta ^F} \geqslant 0$, we have the equality

$$\begin{aligned} \sum _{g \geqslant g_{\varDelta ,n}^{\varDelta ^F}}&N^{\varDelta ,n}_{g,\varDelta ^F} u^{2g-2+|\varDelta |}\\&= \left( \prod _{v \in \varDelta ^F}\frac{1}{|v|} \right) N^{\varDelta ,p,x}_{\mathrm {trop}}(q) \left( (-i)(q^{\frac{1}{2}} -q^{-\frac{1}{2}}) \right) ^{2g_{\varDelta ,n}^{\varDelta ^F}-2+|\varDelta |} \end{aligned}$$

of power series in *u* with rational coefficients, where $q=e^{iu}$.

The proof of Theorem 6 is entirely parallel to the proof of Theorem 5 (Theorem 1 of the Introduction). The required modifications are discussed at the end of Sect. 8.4.

## Gluing and vanishing properties of lambda classes

In this Section, we review some well-known facts: a gluing result for lambda classes, Lemma 7, and then a vanishing result, Lemma 8.

### Lemma 7

Let *B* be a scheme over $\mathbb {C}$. Let $\varGamma$ be a graph, of genus $g_\varGamma$, and let $\pi _V :\mathcal {C}_V \rightarrow B$ be prestable curves over *B* indexed by the vertices *V* of $\varGamma$. For every edge *E* of $\varGamma$, connecting vertices $V_1$ and $V_2$, let $s_{E,1}$ and $s_{E,2}$ be smooth sections of $\pi _{V_1}$ and $\pi _{V_2}$ respectively. Let $\pi :\mathcal {C}\rightarrow B$ be the prestable curve over *B* obtained by gluing together the sections $s_{V_1,E}$ and $s_{V_2,E}$ corresponding to a same edge *E* of $\varGamma$. Then, we have an exact sequence

$$\begin{aligned} 0 \rightarrow \bigoplus _{V \in V(\varGamma )} (\pi _V)_* \omega _{\pi _V} \rightarrow \pi _* \omega _\pi \rightarrow \mathcal {O}^{\oplus g_\varGamma } \rightarrow 0, \end{aligned}$$

where $\omega _{\pi _V}$ and $\omega _\pi$ are the relative line bundles.

### Proof

Let $s_E :B \rightarrow \mathcal {C}$ be the gluing sections. Then we have an exact sequence

$$\begin{aligned} 0 \rightarrow \mathcal {O}_\mathcal {C}\rightarrow \bigoplus _{V \in V(\varGamma )} \mathcal {O}_{\mathcal {C}_V} \rightarrow \bigoplus _{E \in E(\varGamma )} \mathcal {O}_{s_E(B)} \rightarrow 0. \end{aligned}$$

Applying $R \pi _*$, we obtain an exact sequence

$$\begin{aligned} 0&\rightarrow \pi _* \mathcal {O}_{\mathcal {C}} \rightarrow \bigoplus _{V \in V(\varGamma )} \pi _* \mathcal {O}_{\mathcal {C}_V} \rightarrow \bigoplus _{E \in E(\varGamma )} \pi _* \mathcal {O}_{s_E(B)}\\&\rightarrow R^1 \pi _* \mathcal {O}_\mathcal {C}\rightarrow \bigoplus _{V \in V(\varGamma )} R^1 \pi _* \mathcal {O}_{\mathcal {C}_V} \rightarrow 0 . \end{aligned}$$

The kernel of

$$\begin{aligned} R^1 \pi _* \mathcal {O}_\mathcal {C}\rightarrow \bigoplus _{V \in V(\varGamma )} R^1 \pi _* \mathcal {O}_{\mathcal {C}_V} \end{aligned}$$

is a free sheaf of rank $|E(\varGamma )|-|V(\varGamma )|+1=g_\varGamma$. We obtain the desired exact sequence by Serre duality.

Equivalently, if we choose $g_\varGamma$ edges of $\varGamma$ whose complement is a tree, we can understand the morphism

$$\begin{aligned} \pi _* \omega _\pi \rightarrow \mathcal {O}^{\oplus g_\varGamma } \end{aligned}$$

as taking the residues at the corresponding $g_\varGamma$ sections. $\square$

### Lemma 8

Let *B* be a scheme over $\mathbb {C}$. Let $\pi :\mathcal {C}\rightarrow B$ be a prestable curve of arithmetic genus *g* over *B*. For every integer $g'$ such that $0 \leqslant g' \leqslant g$, let $B_{g'}$ be the closed subset of *B* of points *b* such that the dual graph of the curve $\pi ^{-1}(b)$ is of genus $\geqslant g'$. Then the lambda classes $\lambda _j \in H^{2j}(B,\mathbb {Q})$, defined by $\lambda _j = c_j (\pi _{*} \omega _\pi )$, satisfy

$$\begin{aligned} \lambda _j|_{B_{g'}}=0 \end{aligned}$$

in $H^{2j}(B_{g'},\mathbb {Q})$ for all $j>g-g'$.

### Proof

Let ${\tilde{B}}_{g'}$ be the finite cover of $B_{g'}$ given by the possible choices of $g'$ fully separating nodes, i.e. of nodes whose complement is of genus 0. Separating these $g'$ fully separating nodes gives a way to write the pullback of $\mathcal {C}$ to ${\tilde{B}}_{g'}$ as the gluing of curves according to a dual graph $\varGamma$ of genus $g'$. According to Lemma 7, the Hodge bundle of this family of curves has a trivial rank $g'$ quotient. As ${\tilde{B}}_{g'}$ is finite over $B_g'$, it is enough to guarantee the desired vanishing in rational cohomology. $\square$

## Toric degeneration and decomposition formula

In Sect. 4.1, we review the natural link between log geometry and tropical geometry given by tropicalization. In Sect. 4.2, we start the proof of Theorem 1 by considering the Nishinou–Siebert toric degeneration. In Sect. 4.3, we apply the decomposition formula of Abramovich, Chen, Gross, Siebert [3] to this toric degeneration to write the log Gromov–Witten invariants $N_g^{\varDelta ,n}$ in terms of log Gromov–Witten invariants $N_g^{\varDelta , h}$ indexed by parametrized tropical curves $h :\varGamma \rightarrow \mathbb {R}^2$. We use the vanishing result of Sect. 3 to restrict the tropical curves appearing.

### Tropicalization

Log geometry is naturally related to tropical geometry. Every log scheme *X* admits a tropicalization $\varSigma (X)$.

Recall that a log scheme is a scheme *X* endowed with a sheaf of monoids $\mathcal {M}_X$ and a morphism of sheaves of monoids^8^

$$\begin{aligned} \alpha _X :\mathcal {M}_X \rightarrow \mathcal {O}_X , \end{aligned}$$

where $\mathcal {O}_X$ is seen as a sheaf of multiplicative monoids, such that the restriction of $\alpha _X$ to $\alpha _X^{-1}(\mathcal {O}_X^*)$ is an isomorphism.

The ghost sheaf of a log scheme *X* is the sheaf of monoids

$$\begin{aligned} \overline{\mathcal {M}}_X {:}{=}\mathcal {M}_X/\alpha ^{-1}(\mathcal {O}_X^*) . \end{aligned}$$

For the kind of log schemes that we are considering, fine and saturated, the ghost sheaf is of combinatorial nature. In this case, one can think of the log geometry of *X* as a combination of the geometry of the underlying scheme *X* and of the combinatorics of the ghost sheaf $\overline{\mathcal {M}}_X$. Non-trivial interactions between these two aspects of log geometry are encoded in the sequence

$$\begin{aligned} \mathcal {O}_X^* \rightarrow \mathcal {M}_X \rightarrow \overline{\mathcal {M}}_X . \end{aligned}$$

A cone complex is an abstract gluing of convex rational cones along their faces. If *X* is a log scheme, the tropicalization $\varSigma (X)$ of *X* is the cone complex defined by gluing together the convex rational cones ${\text {Hom}}(\overline{\mathcal {M}}_{X,x}, \mathbb {R}_{\geqslant 0})$ for all $x \in X$ according to the natural specialization maps. Tropicalization is a functorial construction. For more details on tropicalization of log schemes, we refer to Appendix B of [24] and Section 2 of [3]. Tropicalization gives a pictorial way to describe the combinatorial part of log geometry contained in the ghost sheaf.

**Examples**

- Let *X* be a toric variety. We can view *X* as a log scheme for the toric divisorial log structure, i.e. the divisorial log stucture with respect to the toric boundary divisor $\partial X$. The sheaf $\mathcal {M}_X$ is the sheaf of functions non-vanishing outside $\partial X$ and $\alpha _X$ is the natural inclusion of $\mathcal {M}_X$ in $\mathcal {O}_X$. The tropicalization $\varSigma (X)$ of *X* is naturally isomorphic as cone complex to the fan of *X*.
- Let $\overline{\mathcal {M}}$ be a monoid whose only invertible element is 0. Let *X* be the log scheme of underlying scheme the point $\mathrm {pt}= {\text {Spec}}\,\, \mathbb {C}$, with $\mathcal {M}_X = \overline{\mathcal {M}} \oplus \mathbb {C}^*$ and
  $$\begin{aligned}&\alpha _X :\overline{\mathcal {M}} \oplus \mathbb {C}^* \rightarrow \mathbb {C}\\&(m, a) \mapsto a \delta _{m, 0} . \end{aligned}$$
  We denote this log scheme as $\mathrm {pt}_{\overline{\mathcal {M}}}$ and such a log scheme is called a log point. By construction, we have $\overline{\mathcal {M}}_{\mathrm {pt}_{\overline{\mathcal {M}}}} = \overline{\mathcal {M}}$ and so the tropicalization $\varSigma (\mathrm {pt}_{\overline{\mathcal {M}}})$ is the cone ${\text {Hom}}(\overline{\mathcal {M}}, \mathbb {R}_{\geqslant 0})$, i.e. the fan of the affine toric variety ${\text {Spec}}\,\mathbb {C}[\overline{\mathcal {M}}] .$
- The log point $\mathrm {pt}_{\mathbb {N}}$ obtained for $\overline{\mathcal {M}}=\mathbb {N}$ is called the standard log point. Its tropicalization is simply $\varSigma (\mathrm {pt}_{\mathbb {N}}) =\mathbb {R}_{\geqslant 0}$, the fan of the affine line $\mathbb {A}^1$.
- The log point $\mathrm {pt}_0$ obtained for $\overline{\mathcal {M}}=0$ is called the trivial log point. Its tropicalization $\varSigma (\mathrm {pt}_0)$ is reduced to a point.
- A stable log map to some relative log scheme $X \rightarrow S$ determines a commutative diagram in the category of log schemes, 
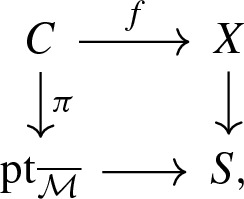
 where $\mathrm {pt}_{\overline{\mathcal {M}}}$ is a log point and $\pi$ is a log smooth proper integral curve. In particular, the scheme underlying *C* is a projective nodal curve with a natural set of smooth marked points. We can take the tropicalization of this diagram to obtain a commutative diagram of cone complexes 
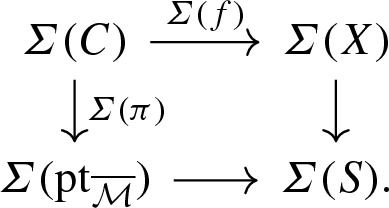
$\varSigma (C)$ is a family of graphs over the cone $\varSigma (\mathrm {pt}_{{\overline{M}}})= {\text {Hom}}(\overline{\mathcal {M}}, \mathbb {R}_{\geqslant 0})$: the fiber of $\varSigma (\pi )$ over a point in the interior of the cone is the dual graph of *C*. Fibers over faces of the cone are contractions of the dual graph. In particular, the fiber over the origin of the cone is obtained by fully contracting the dual graph of *C* to a graph with a unique vertex. If *X* is a toric variety with the toric divisorial log structure and *S* is the trivial log point, then $\varSigma (f)$ is a family of parametrized tropical curves in the fan of *X*. We refer to Section 2.5 of [3] for more details.

### Toric degeneration

Let $\varDelta$ be a balanced configuration of vectors, as in Sect. 2.1, and let *n* be a non-negative integer such that $g_{\varDelta , n} \geqslant 0$. We fix $p=(p_1, \ldots , p_n)$ a configuration of *n* points in $\mathbb {R}^2$ belonging to the open dense subset $U_{\varDelta , n}$ of $(\mathbb {R}^2)^n$ given by Proposition 2. Let $T_{\varDelta , p}$ be the set of *n*-pointed genus $g_{\varDelta , n}$ parametrized tropical curves in $\mathbb {R}^2$ of type $\varDelta$ passing through *p*. The set $T_{\varDelta , p}$ is finite by Proposition 3. Proposition 2 shows that the elements of $T_{\varDelta , p}$ are particularly nice parametrized tropical curves.

We can slightly modify *p* such that $p \in (\mathbb {Q}^2)^n \cap U_{\varDelta , n}$ without changing the combinatorial type of the elements of $T_{\varDelta , p}$ and so without changing the tropical counts $N^{\varDelta ,p}_{\mathrm {trop}}$ and $N^{\varDelta ,p}_{\mathrm {trop}}(q)$. In that case, for every parametrized tropical curve $h :\varGamma \rightarrow \mathbb {R}^2$ in $T_{\varDelta , p}$ and for every vertex *V* of $\varGamma$, we have $h(V) \in \mathbb {Q}^2$ and for every edge *E* of $\varGamma$, we have $\ell (E) \in \mathbb {Q}$. Indeed, the positions *h*(*V*) of vertices in $\mathbb {R}^2$ and the lengths $\ell (E)$ of edges are natural parameters on the moduli space of genus $g_{\varDelta , n}$ parametrized tropical curves of type $\varDelta$ and this moduli space is a rational polyhedron in the space of these parameters. The set $T_{\varDelta , p}$ is obtained as zero dimensional intersection of this rational polyhedron with the rational (because $p \in (\mathbb {Q}^2)^n$) linear space imposing to pass through *p*. It follows that the parameters *h*(*V*) and $\ell (E)$ are rational for elements of $T_{\varDelta , p}$.

We follow the toric degeneration approach introduced by Nishinou and Siebert [38] (see also Mandel and Ruddat [30]). According to [38] Proposition 3.9 and [30] Lemma 3.1, there exists a rational polyhedral decomposition $\mathcal {P}_{\varDelta ,p}$ of $\mathbb {R}^2$ such that

- The asymptotic fan of $\mathcal {P}_{\varDelta ,p}$ is the fan of $X_\varDelta$.
- For every parametrized tropical curve $h :\varGamma \rightarrow \mathbb {R}^2$ in $T_{\varDelta , p}$, the images *h*(*V*) of vertices *V* of $\varGamma$ are vertices of $\mathcal {P}_{\varDelta ,p}$ and the images *h*(*E*) of edges *E* of $\varGamma$ are contained in union of edges of $\mathcal {P}_{\varDelta ,p}$

Remark that the points $p_j$ in $\mathbb {R}^2$ are image of vertices of parametrized tropical curves in $T_{\varDelta , p}$ and so are vertices of $\mathcal {P}_{\varDelta ,p}$.

Given a parametrized tropical curve $h :\varGamma \rightarrow \mathbb {R}^2$ in $T_{\varDelta , p}$, we construct a new parametrized tropical curve ${\tilde{h}} :{\tilde{\varGamma }} \rightarrow \mathbb {R}^2$ by simply adding a genus zero bivalent unpointed vertex to $\varGamma$ at each point $h^{-1}(V)$ for *V* a vertex of $\mathcal {P}_{\varDelta ,p}$ which is not the image by *h* of a vertex of $\varGamma$. The image ${\tilde{h}}(E)$ of each edge *E* of ${\tilde{\varGamma }}$ is now exactly an edge of $\mathcal {P}_{\varDelta ,p}$. The graph ${\tilde{\varGamma }}$ has three types of vertices:

- Trivalent unpointed vertices, coming from $\varGamma$.
- Bivalent pointed vertices, coming from $\varGamma$.
- Bivalent unpointed vertices, not coming from $\varGamma$.

Doing a global rescaling of $\mathbb {R}^2$ if necessary, we can assume that $\mathcal {P}_{\varDelta ,p}$ is an integral polyhedral decomposition, i.e. that all the vertices of $\mathcal {P}_{\varDelta ,p}$ are in $\mathbb {Z}^2$, and that all the lengths $\ell (E)$ of edges *E* of parametrized tropical curves ${\tilde{h}} :{\tilde{\varGamma }} \rightarrow \mathbb {R}^2$, coming from $h :\varGamma \rightarrow \mathbb {R}^2$ in $T_{\varDelta , p}$, are integral.

Taking the cone over $\mathcal {P}_{\varDelta , p} \times \{1\}$ in $\mathbb {R}^2 \times \mathbb {R}$, we obtain the fan of a three dimensional toric variety $X_{\mathcal {P}_{\varDelta , p}}$ equipped with a morphism

$$\begin{aligned} \nu :X_{\mathcal {P}_{\varDelta , p}} \rightarrow \mathbb {A}^1 \end{aligned}$$

coming from the projection $\mathbb {R}^2 \times \mathbb {R}\rightarrow \mathbb {R}$ on the third $\mathbb {R}$ factor. We have $\nu ^{-1}(t) \simeq X_\varDelta$ for every $t \in \mathbb {A}^1 -\{0\}$. The special fiber $X_0 {:}{=}\nu ^{-1}(0)$ is a reducible surface whose irreducible components $X_V$ are toric surfaces in one to one correspondence with the vertices *V* of $\mathcal {P}_{\varDelta ,p}$,

$$\begin{aligned} X_0 = \bigcup _V X_V . \end{aligned}$$

In other words, $\nu :X_{\mathcal {P}_{\varDelta , p}} \rightarrow \mathbb {A}^1$ is a toric degeneration of $X_\varDelta$.

We consider the toric varieties $\mathbb {A}^1$, $X_{\mathcal {P}_{\varDelta , p}}$, $X_\varDelta$ and $X_V$ as log schemes with respect to the toric divisorial log structure. In particular, the toric morphism $\nu$ induces a log smooth morphism

$$\begin{aligned} \nu :X_{\mathcal {P}_{\varDelta , n}} \rightarrow \mathbb {A}^1. \end{aligned}$$

Restricting to the special fiber gives a structure of log scheme on $X_0$ and a log smooth morphism to the standard log point

$$\begin{aligned} \nu _0 :X_0 \rightarrow \mathrm {pt}_{\mathbb {N}}. \end{aligned}$$

From now on, we will denote $\underline{X}_0$ the scheme underlying the log scheme $X_0$. Beware that the toric divisorial log structure that we consider on $X_V$ is not the restriction of the log structure that we consider on $X_0$.

For every $j=1,\ldots ,n$, the ray $\mathbb {R}_{\geqslant 0} (p_j, 1)$ in $\mathbb {R}^2 \times \mathbb {R}$ defines a one-parameter subgroup $\mathbb {C}^{*}_{p_j}$ of $(\mathbb {C}^*)^3 \subset X_{\mathcal {P}_{\varDelta ,n}}$. We choose a point $P_j \in (\mathbb {C}^*)^2$ and we write $Z_{P_j}$ the affine line in $X_{\mathcal {P}_{\varDelta ,n}}$ defined as the closure of the orbit of $(P_j,1)$ under the action of $\mathbb {C}^*_{p_j}$. We have

$$\begin{aligned} Z_{P_j} \cap \nu ^{-1}(1) =Z_{P_j} \cap X_\varDelta = P_j , \end{aligned}$$

and

$$\begin{aligned} P_j^0 \,{:}{=}\,Z_{P_j} \cap \nu ^{-1}(0) \end{aligned}$$

is a point in the dense torus $(\mathbb {C}^*)^2$ contained in the toric component of $X_0$ corresponding to the vertex $p_j$ of $\mathcal {P}_{\varDelta ,p}$. In other words, $Z_{P_j}$ is a section of $\nu$ degenerating $P_j \in X_\varDelta$ to some $P_j^0 \in X_0$.

Recall from Sect. 2.2 that the log Gromov–Witten invariants $N_g^{\varDelta ,n}$ are defined using stable log maps of target $X_\varDelta$,

$$\begin{aligned} N^{\varDelta ,n}_g {:}{=}\int _{[{\overline{M}}_{g,n,\varDelta }]^{\mathrm {virt}}} (-1)^{g-g_{\varDelta ,n}} \lambda _{g-g_{\varDelta ,n}} \prod _{j=1}^n \text {ev}_j^{*}(\mathrm {pt}) , \end{aligned}$$

where ${\overline{M}}_{g,n,\varDelta }$ is the moduli space of *n*-pointed stable log maps to $X_\varDelta$ of genus *g* and of type $\varDelta$.

Let ${\overline{M}}_{g,n,\varDelta }(X_0 / \mathrm {pt}_{\mathbb {N}})$ be the moduli space of *n*-pointed stable log maps to $\pi _0 :X_0 \rightarrow \mathrm {pt}_{\mathbb {N}}$ of genus *g* and of type $\varDelta$. It is a proper Deligne-Mumford stack of virtual dimension

$$\begin{aligned} {\text {vdim}}\,{\overline{M}}_{g,n,\varDelta }(X_0 / \mathrm {pt}_{\mathbb {N}}) ={\text {vdim}}\,{\overline{M}}_{g,n,\varDelta }=g-g_{\varDelta ,n}+2n \end{aligned}$$

and it admits a virtual fundamental class

$$\begin{aligned}{}[{\overline{M}}_{g,n,\varDelta }(X_0/\mathrm {pt}_{\mathbb {N}})]^{\mathrm {virt}} \in A_{g-g_{\varDelta ,n}+2n} ({\overline{M}}_{g,n,\varDelta }(X_0/\mathrm {pt}_{\mathbb {N}}), \mathbb {Q}). \end{aligned}$$

Considering the evaluation morphism

$$\begin{aligned} \text {ev} :{\overline{M}}_{g,n,\varDelta }(X_0 / \mathrm {pt}_{\mathbb {N}}) \rightarrow \underline{X}_0^n \end{aligned}$$

and the inclusion

$$\begin{aligned} \iota _{P^0} :\left( P^0 {:}{=}\left( P_1^0, \ldots , P_n^0\right) \right) \hookrightarrow \underline{X}_0^n , \end{aligned}$$

we can define the moduli space^9^

$$\begin{aligned} {\overline{M}}_{g,n,\varDelta }(X_0 / \mathrm {pt}_{\mathbb {N}}, P^0) {:}{=}{\overline{M}}_{g,n,\varDelta }(X_0 / \mathrm {pt}_{\mathbb {N}}) \times _{\underline{X}_0^n} P^0 , \end{aligned}$$

of stable log maps passing through $P^0$, and by the Gysin refined homomorphism (see Section 6.2 of [16]), a virtual fundamental class

$$\begin{aligned}&[{\overline{M}}_{g,n,\varDelta }(X_0 / \mathrm {pt}_{\mathbb {N}}, P^0)]^{\mathrm {virt}} \,{:}{=}\, \iota _{P^0}^{!}[{\overline{M}}_{g,n,\varDelta }(X_0/ \mathrm {pt}_{\mathbb {N}})]^{\mathrm {virt}}\\&\quad \in A_{g-g_{\varDelta ,n}} ({\overline{M}}_{g,n,\varDelta }(X_0/\mathrm {pt}_{\mathbb {N}}, P^0), \mathbb {Q}). \end{aligned}$$

Remark^10^ that this definition is compatible with [3] because each $P^0_j$, seen as a log morphism $P^0_j :\mathrm {pt}_{\mathbb {N}} \rightarrow X_0$, is strict. This follows from the fact that we have chosen $P^0_j$ in the dense torus $(\mathbb {C}^{*})^2$ contained in the toric component of $X_0$ dual to the vertex $p_j$ of $\mathcal {P}_{\varDelta ,p}$. If it were not the case,^11^ then, following Section 6.3.2 of [3], the definition of ${\overline{M}}_{g,n,\varDelta }(X_0 / \mathrm {pt}_{\mathbb {N}}, P^0)$ should have been replaced by a fiber product in the category of fs log stacks and $[{\overline{M}}_{g,n,\varDelta }(X_0 / \mathrm {pt}_{\mathbb {N}}, P^0)]^{\mathrm {virt}}$ should have been defined by some perfect obstruction theory directly on ${\overline{M}}_{g,n,\varDelta }(X_0 / \mathrm {pt}_{\mathbb {N}}, P^0)$.

By deformation invariance of the virtual fundamental class on moduli spaces of stable log maps in log smooth families, we have

$$\begin{aligned} N^{\varDelta ,n}_g = \int _{[{\overline{M}}_{g,n,\varDelta }(X_0/\mathrm {pt}_{\mathbb {N}}, P^0)]^{\mathrm {virt}}} (-1)^{g-g_{\varDelta ,n}} \lambda _{g-g_{\varDelta ,n}} . \end{aligned}$$

### Decomposition formula

As the toric degeneration breaks the toric surface $X_\varDelta$ into many pieces, irreducible components of the special fiber $X_0$, one can similarly expect that it breaks the moduli space ${\overline{M}}_{g,n,\varDelta }$ of stable log maps to $X_\varDelta$ into many pieces, irreducible components of the moduli space ${\overline{M}}_{g,n,\varDelta }(X_0/\mathrm {pt}_{\mathbb {N}})$ of stable log maps to $X_0$. Tropicalization gives a way to understand the combinatorics of this breaking into pieces.

As we recalled in Sect. 4.1, a *n*-pointed stable log map to $X_0 / \mathrm {pt}_{\mathbb {N}}$ of type $\varDelta$ gives a commutative diagram of log schemes 
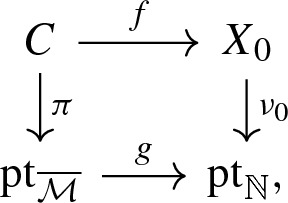
 which can be tropicalized in a commutative diagram of cone complexes 
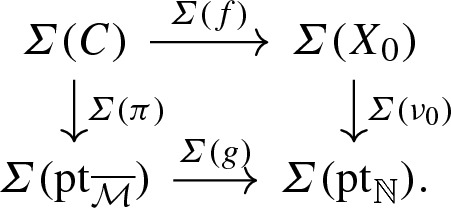
 We have $\varSigma (\mathrm {pt}_{\mathbb {N}}) \simeq \mathbb {R}_{\geqslant 0}$ and the fiber $\varSigma (\nu _0)^{-1}(1)$ is naturally identified with $\mathbb {R}^2$ equipped with the polyhedral decomposition $\mathcal {P}_{\varDelta ,p}$, whose asymptotic fan is the fan of $X_\varDelta$. So the above diagram gives a family over the polyhedron $\varSigma (g)^{-1}(1)$ of *n*-pointed parametrized tropical curves in $\mathbb {R}^2$ of type $\varDelta$

The moduli space ${\overline{M}}_{g,n,\varDelta }^{\mathrm {trop}}$ of *n*-pointed genus *g* parametrized tropical curves in $\mathbb {R}^2$ of type $\varDelta$ is a rational polyhedral complex. If ${\overline{M}}_{g,n,\varDelta }^{\mathrm {trop}}$ were the tropicalization of ${\overline{M}}_{g,n,\varDelta }(X_0/\mathrm {pt}_{\mathbb {N}})$ (seen as a log stack over $\mathrm {pt}_{\mathbb {N}}$), then ${\overline{M}}_{g,n,\varDelta }^{\mathrm {trop}}$ would be the dual intersection complex of ${\overline{M}}_{g,n,\varDelta }^{\mathrm {trop}}$. In particular, irreducible components of ${\overline{M}}_{g,n,\varDelta }(X_0/\mathrm {pt}_{\mathbb {N}})$ would be in one to one correspondence with the 0-dimensional faces of ${\overline{M}}_{g,n,\varDelta }^{\mathrm {trop}}$. As the polyhedral decomposition of ${\overline{M}}_{g,n,\varDelta }^{\mathrm {trop}}$ is induced by the combinatorial type of tropical curves, the 0-dimensional faces of ${\overline{M}}_{g,n,\varDelta }^{\mathrm {trop}}$ correspond to the rigid parametrized tropical curves, see Definition 4.3.1 of [3], i.e. to parametrized tropical curves which are not contained in a non-trivial family of parametrized tropical curves of the same combinatorial type.

According to the decomposition formula of Abramovich, Chen, Gross, Siebert [3], this heuristic description of the pieces of ${\overline{M}}_{g,n,\varDelta }(X_0/\mathrm {pt}_{\mathbb {N}})$ is correct at the virtual level: one can express $[{\overline{M}}_{g,n,\varDelta }(X_0/\mathrm {pt}_{\mathbb {N}}, P^0)]^{\mathrm {virt}}$ as a sum of contributions indexed by rigid tropical curves.

Let ${\tilde{h}} :{\tilde{\varGamma }} \rightarrow \mathbb {R}^2$ be a *n*-pointed genus *g* rigid parametrized tropical curve to $\mathbb {R}^2$ of type $\varDelta$ passing through *p*. For every *V* vertex of ${\tilde{\varGamma }}$, let $\varDelta _V$ be the balanced collection of vectors $v_{V,E}$ for all edges *E* adjacent to *V*. Using the notations of Sect. 2.1 that we used all along for $\varDelta$ but now for $\varDelta _V$, the toric surface $X_{\varDelta _V}$ is the irreducible component of $X_0$ corresponding to the vertex *h*(*V*) of the polyhedral decomposition $\mathcal {P}_{\varDelta , p}$.

A *n*-pointed genus *g* stable log map to $X^0$ of type $\varDelta$ passing through $P^0$ and marked by ${\tilde{h}}$ is the following data, see [3], Definition 4.4.1,^12^

- A *n*-pointed genus *g* stable log map $f :C/\mathrm {pt}_{\overline{\mathcal {M}}} \rightarrow X_0 /\mathrm {pt}_{\mathbb {N}}$ of type $\varDelta$ passing through $P^0$.
- For every vertex *V* of ${\tilde{\varGamma }}$, an ordinary stable map $f_V :C_V \rightarrow X_{\varDelta _V}$ of class $\beta _{\varDelta _V}$ with marked points $x_v$ for every $v \in \varDelta _V$, such that $f_V(x_v) \in D_v$, where $D_v$ is the prime toric divisor of $X_{\varDelta _V}$ dual to the ray $\mathbb {R}_{\geqslant 0} v$.

These data must satisfy the following compatibility conditions: the gluing of the curves $C_V$ along the points corresponding to the edges of ${\tilde{\varGamma }}$ is isomorphic to the curve underlying the log curve *C*, and the corresponding gluing of the maps $f_V$ is the map underlying the log map *f*.

By [3], the moduli space ${\overline{M}}_{g,n,\varDelta }^{{\tilde{h}},P^0}$ of *n*-pointed genus *g* stable log maps of type $\varDelta$ passing through $P^0$ and marked by ${\tilde{h}}$ is a proper Deligne-Mumford stack, equipped with a natural virtual fundamental class $[{\overline{M}}^{{\tilde{h}},P^0}_{g,n,\varDelta }]^{\mathrm {virt}}$. Forgetting the marking by ${\tilde{h}}$ gives a morphism

$$\begin{aligned} i_{{\tilde{h}}} :{\overline{M}}^{{\tilde{h}},P^0}_{g,n,\varDelta } \rightarrow {\overline{M}}_{g,n,\varDelta }\left( X_0/\mathrm {pt}_{\mathbb {N}}, P^0\right) . \end{aligned}$$

According to the decomposition formula, [3] Theorem 6.3.9, we have

$$\begin{aligned} \left[ {\overline{M}}_{g,n,\varDelta }(X_0/\mathrm {pt}_{\mathbb {N}}, P^0)\right] ^{\mathrm {virt}} = \sum _{{\tilde{h}}} \frac{n_{{\tilde{h}}}}{|\mathrm {Aut} ({\tilde{h}})|} (i_{{\tilde{h}}})_* \left[ {\overline{M}}^{{\tilde{h}},P^0}_{g,n,\varDelta }\right] ^{\mathrm {virt}} , \end{aligned}$$

where the sum is over the *n*-pointed genus *g* rigid parametrized tropical curves to $(\mathbb {R}^2, \mathcal {P}_{\varDelta ,p})$ of type $\varDelta$ passing through *p*, $n_{{\tilde{h}}}$ is the smallest positive integer such that the scaling of ${\tilde{h}}$ by $n_{{\tilde{h}}}$ has integral vertices and integral lengths, and $|\mathrm {Aut}({\tilde{h}})|$ is the order of the automorphism group of ${\tilde{h}}$.

Recall from Proposition 2 that a parametrized tropical curve $h :\varGamma \rightarrow \mathbb {R}^2$ in $T_{\varDelta , p}$ has a source graph $\varGamma$ of genus $g_{\varDelta ,n}$ and that all vertices *V* of $\varGamma$ are of genus zero: $g(V)=0$. In Sect. 4.2, we explained that the polyhedral decomposition $\mathcal {P}_{\varDelta ,p}$ defines a new parametrized tropical ${\tilde{h}} :{\tilde{\varGamma }} \rightarrow \mathbb {R}^2$, for each $h :\varGamma \rightarrow \mathbb {R}^2$ in $T_{\varDelta ,p}$, by addition of unmarked genus zero bivalent vertices. Given such parametrized tropical curve ${\tilde{h}} :{\tilde{\varGamma }} \rightarrow \mathbb {R}^2$, one can construct genus *g* parametrized tropical curves by changing only the genus of vertices *g*(*V*) so that

$$\begin{aligned} \sum _{V \in V(\varGamma )} g(V) = g-g_{\varDelta , n} . \end{aligned}$$

We denote $T_{\varDelta , p}^g$ the set of genus *g* parametrized tropical curves obtained in this way.

#### Lemma 9

Parametrized tropical curves ${\tilde{h}} :{\tilde{\varGamma }} \rightarrow \mathbb {R}^2$ in $T_{\varDelta , p}^g$ are rigid. Furthermore, for such ${\tilde{h}}$, we have $n_{{\tilde{h}}}=1$ and $|\mathrm {Aut} ({\tilde{h}})|=1$.

#### Proof

The rigidity of parametrized tropical curves in $T_{\varDelta ,p}^g$ follows from the rigidity of parametrized tropical curves in $T_{\varDelta ,p}$ because the genera attached to the vertices cannot change under a deformation preserving the combinatorial type, and added bivalent vertices to go from $\varGamma$ to ${\tilde{\varGamma }}$ are mapped to vertices of $\mathcal {P}_{\varDelta ,p}$ and so cannot move without changing the combinatorial type.

We have $n_{{\tilde{h}}}=1$ because in Sect. 4.2, we have chosen the polyhedral decomposition $\mathcal {P}_{\varDelta ,p}$ to be integral: vertices of ${\tilde{h}}$ map to integral points of $\mathbb {R}^2$ and edges *E* of ${\tilde{\varGamma }}$ have integral lengths $\ell (E)$. We have $|\mathrm {Aut} ({\tilde{h}})|=1$ because ${\tilde{h}}$ is an immersion. The genus of vertices never enters in the above arguments. $\square$

For every ${\tilde{h}} :{\tilde{\varGamma }} \rightarrow \mathbb {R}^2$ parametrized tropical curve in $T_{\varDelta ,p}^g$, we define

$$\begin{aligned} N^{\varDelta ,n}_{g,{\tilde{h}}} \,{:}{=}\, \int _{[{\overline{M}}_{g,n,\varDelta }^{{\tilde{h}},P^0}]^{\mathrm {virt}}} (-1)^{g-g_{\varDelta ,n}} \lambda _{g-g_{\varDelta ,n}} . \end{aligned}$$

#### Proposition 10

For every $\varDelta$, *n* and $g \geqslant g_{\varDelta , n}$, we have

$$\begin{aligned} N^{\varDelta ,n}_g = \sum _{{\tilde{h}} \in T_{\varDelta , p}^g} N^{\varDelta , n}_{g, {\tilde{h}}} . \end{aligned}$$

#### Proof

This follows from the decomposition formula and from the vanishing property of lambda classes.

If ${\tilde{h}}$ is a rigid parametrized tropical curve of genus $g >g_{\varDelta , n}$, then every point in ${\overline{M}}_{g,n,\varDelta }^{{\tilde{h}},P^0}$ is a stable log map whose tropicalization has genus $g>g_{\varDelta ,n}$. In particular, the dual intersection complex of the source curve has genus $g > g_{\varDelta , n}$. By Lemma 8, $\lambda _{g-g_{\varDelta ,n}}$ is zero on restriction to such family of curves. $\square$

**Example** The generic way to deform a parametrized tropical curve in $T^g_{\varDelta , p}$ is to open *g*(*V*) small cycles in place of a vertex of genus *g*(*V*). When the cycles coming from various vertices grow and meet, we can obtain curves with vertices of valence strictly greater than three which can be rigid. Proposition 10 guarantees that such rigid curves do not contribute in the decomposition formula after integration of the lambda class.

Below is an illustration of a genus one vertex opening in one cycle and growing until forming a 4-valent vertex.

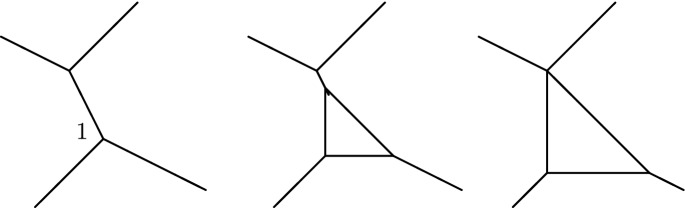



## Non-torically transverse stable log maps in $X_\varDelta$

Let $\varDelta$ be a balanced collections of vectors in $\mathbb {Z}^2$, as in Sect. 2.1. We consider the toric surface $X_\varDelta$ with the toric divisorial log structure. In this Section, we prove some general properties of stable log maps of type $\varDelta$ in $X_\varDelta$, using as tool the tropicalization procedure reviewed in Sect. 4.1.

We say that a stable log map $(f :C/ \mathrm {pt}_{\overline{\mathcal {M}}} \rightarrow X_\varDelta )$ to $X_\varDelta$ is torically transverse^13^ if its image does not contain any of the torus fixed points of $X_\varDelta$, i.e. if its image does not pass through the “corners” of the toric boundary divisor $\partial X_\varDelta$. The difficulty of log Gromov–Witten theory, with respect to relative Gromov–Witten theory for example, comes from the stable log maps which are not torically transverse: the “corners” of $\partial X_\varDelta$ are the points where $\partial X_\varDelta$ is not smooth and so are exactly the points where the log structure of $X_\varDelta$ is locally more complicated that the divisorial log structure along a smooth divisor.

The following Proposition is a structure result for stable log maps of type $\varDelta$ which are not torically transverse. Combined with vanishing properties of lambda classes reviewed in Sect. 3, this will give us in Sect. 7 a way to completely discard stable log maps which are not torically transverse.

### Proposition 11

Let $f :C/\mathrm {pt}_{\overline{\mathcal {M}}} \rightarrow X_\varDelta$ be a stable log map to $X_\varDelta$ of type $\varDelta$. Let $\varSigma (f) :\varSigma (C)/\varSigma (\mathrm {pt}_{\mathbb {N}}) \rightarrow \varSigma (X_\varDelta )$ be the family of tropical curves obtained as tropicalization of *f*. Assume that *f* is not torically transverse and that the unbounded edges of the fibers of $\varSigma (f)$ are mapped to rays of the fan of $X_\varDelta$. Then the dual graph of *C* has positive genus, i.e. *C* contains at least one non-separating node.

### Proof

Recall that $\varSigma (f)$ is a family over the cone $\varSigma (\mathrm {pt}_{\mathbb {N}})={\text {Hom}}(\overline{\mathcal {M}}, \mathbb {R}_{\geqslant 0})$ of parametrized tropical curves in $\mathbb {R}^2$. We assume that the unbounded edges of these parametrized tropical curves are mapped to rays of the fan of $X_\varDelta$.

We fix a point in the interior of the cone ${\text {Hom}}(\overline{\mathcal {M}}, \mathbb {R}_{\geqslant 0})$ and we consider the corresponding parametrized tropical curve $h :\varGamma \rightarrow \mathbb {R}^2$ in $\mathbb {R}^2$. Combinatorially, $\varGamma$ is the dual graph of *C*.

### Lemma 12

There exists a vertex *V* of $\varGamma$ mapping away from the origin in $\mathbb {R}^2$ and a non-contracted edge *E* adjacent to *V* such that *h*(*E*) is not included in a ray of the fan of $X_\varDelta$.

### Proof

We are assuming that *f* is not torically transverse. This means that at least one component of *C* maps dominantly to a component of the toric boundary divisor $\partial X_\varDelta$ or that at least one component of *C* is contracted to a torus fixed point of $X_\varDelta$.

If one component of *C* is contracted to a torus fixed point of $X_\varDelta$, then we are done because the corresponding vertex *V* of $\varGamma$ is mapped away from the origin and from the rays of the fan of $X_\varDelta$, and any non-contracted edge of $\varGamma$ adjacent to *V* is not mapped to a ray of the fan of $X_\varDelta$. Remark that there exists such non-contracted edge because if not, as $\varGamma$ is connected, all the vertices of $\varGamma$ would be mapped to *h*(*V*) and so the curve *C* would be entirely contracted to a torus fixed point, contradicting $\beta _\varDelta \ne 0$.

So we can assume that no component of *C* is contracted to a torus fixed point, i.e. that all the vertices of $\varGamma$ are mapped either to the origin or to a point on a ray of the fan of $X_\varDelta$, and that at least one component of *C* maps dominantly to a component of $\partial X_\varDelta$. We argue by contradiction by assuming further that every edge of $\varGamma$ is either contracted to a point or mapped inside a ray of the fan of $X_\varDelta$.

Let $\varGamma _0$ be the subgraph of $\varGamma$ formed by vertices mapping to the origin and edges between them. For every ray $\rho$ of the fan of $X_\varDelta$, let $\varDelta _\rho$ be the set of $v \in \varDelta$ such that $\mathbb {R}_{\geqslant 0} v=\rho$, and let $\varGamma _\rho$ be the subgraph of $\varGamma$ formed by vertices of $\varGamma$ mapping to the ray $\rho$ away from the origin and the edges between them.

By our assumption, there is no edge in $\varGamma$ connecting $\varGamma _\rho$ and $\varGamma _{\rho '}$ for two different rays $\rho$ and $\rho '$. For every ray $\rho$, let $E(\varGamma _0, \varGamma _\rho )$ the set of edges of $\varGamma$ connecting a vertex $V_0(E)$ of $\varGamma _0$ and a vertex $V_\rho (E)$ of $\varGamma _\rho$. It follows from the balancing condition that, for every ray $\rho$, we have

$$\begin{aligned} \sum _{E \in E(\varGamma _0, \varGamma _\rho )} v_{V_0(E),E} = \sum _{v \in \varDelta _\rho } v . \end{aligned}$$

Let $C_0$ be the curve obtained by taking the components of *C* intersecting properly the toric boundary divisor $\partial X_\varDelta$. The dual graph of $C_0$ is $\varGamma _0$ and the total intersection number of $C_0$ with the toric divisor $D_\rho$ is

$$\begin{aligned} \sum _{E \in E(\varGamma _0, \varGamma _\rho )} |v_{V_0(E),E}|, \end{aligned}$$

where $|v_{V_0(E),E}|$ is the divisibility of $v_{V_0(E),E}$ in $\mathbb {Z}^2$, i.e. the multiplicity of the corresponding intersection point of $C_0$ and $D_\rho$.

From the previous equality, we obtain that the intersection numbers of $C_0$ with the components of $\partial X_\varDelta$ are equal to the intersection numbers of *C* with the components of $\partial X_\varDelta$ so $[f(C_0)]=\beta _\varDelta$. It follows that all the components of *C* not in $C_0$ are contracted, which contradicts the fact that at least one component of *C* maps dominantly to a component of $\partial X_\varDelta$. $\square$

We continue the proof of Proposition 11. By Lemma 12, there exists a vertex *V* of $\varGamma$ mapping away from the origin in $\mathbb {R}^2$ and a non-contracted edge *E* adjacent to *V* such that *h*(*E*) is not included in a ray of the fan of $X_\varDelta$. We will use (*V*, *E*) as initial data for a recursive construction of a non-trivial cycle in $\varGamma$.

There exists a unique two-dimensional cone of the fan of $X_\varDelta$, containing $h(V) \in \mathbb {R}^2-\{0\}$ and delimited by rays $\rho _1$ and $\rho _2$, such that the rays $\rho _1$, $\mathbb {R}_{\geqslant 0} h(V)$ and $\rho _2$ are ordered in the clockwise way and such that $h(V) \in \rho _1$ if *h*(*V*) is on a ray. Let $v_1$ and $v_2$ be vectors in $\mathbb {R}^2-\{0\}$ such that $\rho _1 =\mathbb {R}_{\geqslant 0}v_1$ and $\rho _2=\mathbb {R}_{\geqslant 0}v_2$. The vectors $v_1$ and $v_2$ form a basis of $\mathbb {R}^2$ and for every $v \in \mathbb {R}^2$, we write $(v,v_1)$ and $(v,v_2)$ for the coordinates of *v* in this basis, i.e. the real numbers such that

$$\begin{aligned} v=(v,v_1)v_1+(v,v_2)v_2 . \end{aligned}$$

By construction, we have $(h(V), v_1) > 0$ and $(h(V), v_2) \geqslant 0$. As $v_{V,E} \ne 0$, we have $(v_{V,E}, v_1) \ne 0$ or $(v_{V,E}, v_2) \ne 0$.

If $(v_{V,F}, v_2)=0$ for every edge *F* adjacent to *V*, then $(v_{V,E}, v_1) \ne 0$ and $(h(V), v_2)>0$. In particular, *E* is not an unbounded edge. By the balancing condition, up to replacing *E* by another edge adjacent to *V*, one can assume that $(v_{V,E}, v_1)>0$. Then, the edge *E* is adjacent to another vertex $V'$ with $(h(V'), v_1)>(h(V),v_1)$ and $(h(V'),v_2) =(h(V),v_2)$. By the balancing condition, there exists an edge $E'$ adjacent to $V'$ such that $(v_{V',E'}, v_1)>0.$ If $(v_{V,F'}, v_2)=0$ for every edge $F'$ adjacent to $V'$, then in particular we have $(v_{V,E'}, v_2)=0$ and so $E'$ is adjacent to another vertex $V''$ with $(h(V''), v_1)>(h(V'), v_1)$ and $(h(V''), v_2)=(h(V'), v_2)$, and we can iterate the argument. Because $\varGamma$ has finitely many vertices, this process has to stop: there exists a vertex ${\tilde{V}}$ in the cone generated by $\rho _1$ and $\rho _2$ and an edge $\tilde{E}$ adjacent to ${\tilde{V}}$ such that $(v_{{\tilde{V}},\tilde{E}}, v_2) \ne 0$.

The upshot of the previous paragraph is that, up to changing *V* and *E*, one can assume that $(v_{V,E}, v_2) \ne 0$. By the balancing condition, up to replacing *E* by another edge adjacent to *V*, one can assume that $(v_{V,E}, v_2)>0$. The edge *E* is adjacent to another vertex $V'$ with $(h(V'),v_2)>(h(V), v_2)$. By the balancing condition, one can find an edge $E'$ adjacent to $V'$ such that $(v_{V',E'}, v_2)>0$. If $h(V')$ is in the interior of the cone generated by $\rho _1$ and $\rho _2$, then $E'$ is not an unbounded edge and so is adjacent to another vertex $V''$ with $(h(V''),v_2)> (h(V'),v_2)$. Repeating this construction, we obtain a sequence of vertices of image in the cone generated by $\rho _1$ and $\rho _2$. Because $\varGamma$ has finitely many vertices, this process has to terminate: there exists a vertex ${\tilde{V}}$ of $\varGamma$ such that $h({\tilde{V}}) \in \rho _2$ and connected to *V* by a path of edges mapping to the interior of the cone delimited by $\rho _1$ and $\rho _2$.

Repeating the argument starting from ${\tilde{V}}$, and so on, we construct a path of edges in $\varGamma$ whose projection in $\mathbb {R}^2$ intersects successive rays in the clockwise order. Because the combinatorial type of $\varGamma$ is finite, this path has to close eventually and so $\varGamma$ contains a non-trivial closed cycle, i.e. $\varGamma$ has positive genus. $\square$

**Remark** It follows from Proposition 11 that the ad hoc genus zero invariants defined in terms of relative Gromov–Witten invariants of some open geometry used by Gross, Pandharipande, Siebert in [23] (Section 4.4), and Gross, Hacking, Keel in [22] (Section 3.1), coincide with log Gromov–Witten invariants.^14^ In fact, our proof of Proposition 11 can be seen as a tropical analogue of the main properness argument of [23] (Proposition 4.2) which guarantees that the ad hoc invariants are well-defined.

## Statement of the gluing formula

We continue the proof of Theorem 1 started in Sect. 4. In Sect. 6, we state a gluing formula, Corollary 16, expressing the invariants $N_{g,{\tilde{h}}}^{\varDelta ,n}$ attached to a parametrized tropical curve ${\tilde{h}} :{\tilde{\varGamma }} \rightarrow \mathbb {R}^2$ in terms of invariants $N^{1,2}_{g,V}$ attached to the vertices *V* of $\varGamma$. This gluing formula is proved in Sect. 7, using the structure result of Sect. 5 and the vanishing result of Sect. 3 to reduce the argument to the locus of torically transverse stable log maps.

### Preliminaries

We fix ${\tilde{h}} :{\tilde{\varGamma }} \rightarrow \mathbb {R}^2$ a parametrized tropical curve in $T_{\varDelta , p}^g$. The purpose of the gluing formula is to write the log Gromov–Witten invariant

$$\begin{aligned} N^{\varDelta ,n}_{g,{\tilde{h}}} = \int _{[{\overline{M}}_{g,n,\varDelta }^{{\tilde{h}},P^0}]^{\mathrm {virt}}} (-1)^{g-g_{\varDelta ,n}} \lambda _{g-g_{\varDelta ,n}} , \end{aligned}$$

introduced in Sect. 4.3, in terms of log Gromov–Witten invariants of the toric surfaces $X_{\varDelta _V}$ attached to the vertices *V* of ${\tilde{\varGamma }}$. Recall from Sect. 4.2 that ${\tilde{\varGamma }}$ has three types of vertices:

- Trivalent unpointed vertices, coming from $\varGamma$.
- Bivalent pointed vertices, coming from $\varGamma$.
- Bivalent unpointed vertices, not coming from $\varGamma$.

According to Lemma 4.20 of Mikhalkin [34], the connected components of the complement of the bivalent pointed vertices of ${\tilde{\varGamma }}$ are trees with exactly one unbounded edge.

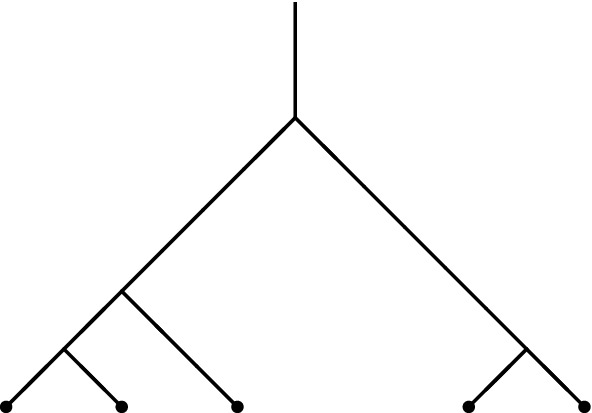



In particular, we can fix an orientation of edges of ${\tilde{\varGamma }}$ consistently from the bivalent pointed vertices to the unbounded edges. Every trivalent vertex of ${\tilde{\varGamma }}$ has two ingoing and one outgoing edges with respect to this orientation. Every bivalent pointed vertex has two outgoing edges with respect to this orientation. Every bivalent unpointed vertex has one ingoing and one outgoing edges with respect to this orientation.

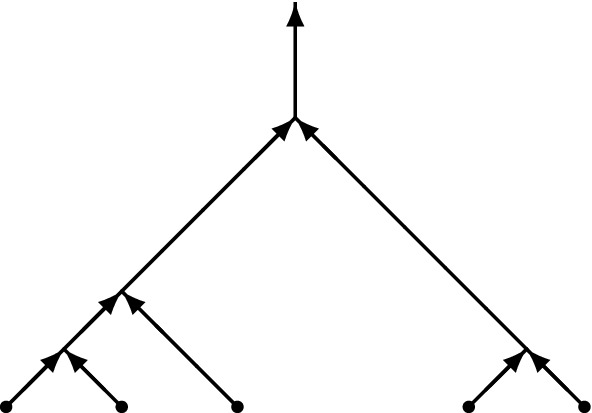



### Contribution of trivalent vertices

Let *V* be a trivalent vertex of ${\tilde{\varGamma }}$. Let ${\overline{M}}_{g,\varDelta _V}$ be the moduli space of stable log maps to $X_{\varDelta _V}$ of genus *g* and of type $\varDelta _V$. It has virtual dimension

$$\begin{aligned} {\text {vdim}}\,{\overline{M}}_{g,\varDelta _V}=g+2 , \end{aligned}$$

and admits a virtual fundamental class

$$\begin{aligned}{}[{\overline{M}}_{g,\varDelta _V}]^{\mathrm {virt}} \in A_{g+2}({\overline{M}}_{g,\varDelta _V}, \mathbb {Q}). \end{aligned}$$

Let $E_V^{\mathrm {in},1}$ and $E_V^{\mathrm {in},2}$ be the two ingoing edges adjacent to *V*, and let $E_V^{\mathrm {out}}$ be the outgoing edge adjacent to *V*. Let $D_{E_V^{\mathrm {in},1}}$, $D_{E_V^{\mathrm {in},2}}$ and $D_{E_V^{\mathrm {out}}}$ be the corresponding toric divisors of $X_{\varDelta _V}$. We have evaluation morphisms

$$\begin{aligned} \left( \text {ev}_V^{E_V^{\mathrm {in},1}}, \text {ev}_V^{E_V^{\mathrm {in},2}}, \text {ev}_V^{E_V^{\mathrm {out}}}\right) :{\overline{M}}_{g,\varDelta _V} \rightarrow D_{E_V^{\mathrm {in},1}} \times D_{E_V^{\mathrm {in},2}} \times D_{E_V^{\mathrm {out}}} . \end{aligned}$$

We define

$$\begin{aligned} N_{g,V}^{1, 2} \,{:}{=}\, \int _{[{\overline{M}}_{g,\varDelta _V}]^{\mathrm {virt}}}(-1)^g \lambda _g \left( \text {ev}_V^{E_V^{\mathrm {in},1}}\right) ^* \left( \mathrm {pt}_{E_V^{\mathrm {in},1}}\right) \left( \text {ev}_V^{E_V^{\mathrm {in},2}}\right) ^* \left( \mathrm {pt}_{E_V^{\mathrm {in},2}}\right) , \end{aligned}$$

where $\mathrm {pt}_{E_V^{\mathrm {in},1}} \in A^1 (D_{E_V^{\mathrm {in},1}})$ and $\mathrm {pt}_{E_V^{\mathrm {in},2}} \in A^1(D_{E_V^{\mathrm {in},2}})$ are classes of a point on $D_{E_V^{\mathrm {in},1}}$ and $D_{E_V^{\mathrm {in},2}}$ respectively.

### Contribution of bivalent pointed vertices

Let *V* be a bivalent pointed vertex of ${\tilde{\varGamma }}$. Let ${\overline{M}}_{g,\varDelta _V}$ be the moduli space of 1-pointed^15^ stable log maps to $X_{\varDelta _V}$ of genus *g* and of type $\varDelta _V$. It has virtual dimension

$$\begin{aligned} {\text {vdim}}\,{\overline{M}}_{g,\varDelta _V}=g+2 , \end{aligned}$$

and admits a virtual fundamental class

$$\begin{aligned}{}[{\overline{M}}_{g,\varDelta _V}]^{\mathrm {virt}} \in A_{g+2}({\overline{M}}_{g,\varDelta _V}, \mathbb {Q}). \end{aligned}$$

We have the evaluation morphism at the extra marked point,

$$\begin{aligned} {\text {ev}}:{\overline{M}}_{g,\varDelta _V} \rightarrow X_{\varDelta _V} , \end{aligned}$$

and we define

$$\begin{aligned} N_{g,V}^{1,2} \,{:}{=}\, \int _{[{\overline{M}}_{g,\varDelta _V}]^{\mathrm {virt}}}(-1)^g \lambda _g \text {ev}^* (\mathrm {pt}) , \end{aligned}$$

where $\mathrm {pt} \in A^2 (X_{\varDelta _V})$ is the class of a point on $X_{\varDelta _V}$.

### Contribution of bivalent unpointed vertices

Let *V* be a bivalent unpointed vertex of ${\tilde{\varGamma }}$. Let ${\overline{M}}_{g,\varDelta _V}$ be the moduli space of stable log maps to $X_{\varDelta _V}$ of genus *g* and of type $\varDelta _V$. It has virtual dimension

$$\begin{aligned} {\text {vdim}}\,{\overline{M}}_{g,\varDelta _V}=g+1 , \end{aligned}$$

and admits a virtual fundamental class

$$\begin{aligned}{}[{\overline{M}}_{g,\varDelta _V}]^{\mathrm {virt}} \in A_{g+1}({\overline{M}}_{g,\varDelta _V}, \mathbb {Q}). \end{aligned}$$

Let $E_V^{\mathrm {in}}$ be the ingoing edge adjacent to *V* and $E_V^{\mathrm {out}}$ the outgoing edge adjacent to *V*. Let $D_{E_V^{\mathrm {in}}}$ and $D_{E_V^{\mathrm {out}}}$ be the corresponding toric divisors of $X_{\varDelta _V}$. We have evaluation morphisms

$$\begin{aligned} \left( \mathrm {ev}_V^{E_V^{\mathrm {in}}}, \mathrm {ev}_V^{E_V^{\mathrm {out}}} \right) :{\overline{M}}_{g,\varDelta _V} \rightarrow D_{E_V^{\mathrm {in}}}\times D_{E_V^{\mathrm {out}}} . \end{aligned}$$

We define

$$\begin{aligned} N_{g,V}^{1,2} \,{:}{=}\, \int _{[{\overline{M}}_{g,\varDelta _V}]^{\mathrm {virt}}}(-1)^g \lambda _g \left( \mathrm {ev}_V^{E_V^{\mathrm {in}}}\right) ^* \left( \mathrm {pt}_{E_V^{\mathrm {in}}}\right) , \end{aligned}$$

where $\mathrm {pt}_{E_V^{\mathrm {in}}} \in A^1 (D_{E_V^{\mathrm {in},1}})$ is the class of a point on $D_{E_V^{\text {in}}}$.

### Statement of the gluing formula

The following gluing formula expresses the log Gromov–Witten invariant $N^{\varDelta , n}_{g, {\tilde{h}}}$ attached to a parametrized tropical curve ${\tilde{h}} :{\tilde{\varGamma }} \rightarrow \mathbb {R}^2$ in terms of the log Gromov–Witten invariants $N^{1,2}_{g,V}$ attached to the vertices *V* of ${\tilde{\varGamma }}$ and of the weights *w*(*E*) of the edges of ${\tilde{\varGamma }}$.

#### Proposition 13

For every ${\tilde{h}} :{\tilde{\varGamma }} \rightarrow \mathbb {R}^2$ parametrized tropical curve in $T_{\varDelta , p}^g$, we have

$$\begin{aligned} N_{g, {\tilde{h}}}^{\varDelta , n} = \left( \prod _{V \in V({\tilde{\varGamma }})} N_{g(V), V}^{1, 2} \right) \left( \prod _{E \in E_f({\tilde{\varGamma }})} w(E) \right) , \end{aligned}$$

where the first product is over the vertices of ${\tilde{\varGamma }}$ and the second product is over the bounded edges of ${\tilde{\varGamma }}$.

The proof of Proposition 13 is given in Sect. 7.

In the following Lemmas, we compute the contributions $N^{1,2}_{g(V), V}$ of the bivalent vertices.

#### Lemma 14

Let *V* be a bivalent pointed vertex of ${\tilde{\varGamma }}$. Then we have

$$\begin{aligned} N^{1,2}_{g,V}=0 \end{aligned}$$

for every $g >0$, and

$$\begin{aligned} N^{1,2}_{0,V}=1 \end{aligned}$$

for $$g=0$$.

#### Proof

Let *w* be the weight of the two edges of ${\tilde{\varGamma }}$ adjacent to *V*. We can take $X_{\varDelta _V}=\mathbb {P}^1 \times \mathbb {P}^1$ and $\beta _{\varDelta _V} = w ([\mathbb {P}^1] \times [\mathrm {pt}])$. We have the evaluation map at the extra marked point

$$\begin{aligned} \text {ev} :{\overline{M}}_{g, \varDelta _V} \rightarrow \mathbb {P}^1 \times \mathbb {P}^1 . \end{aligned}$$

We fix a point $p=(p_1,p_2) \in \mathbb {C}^* \times \mathbb {C}^* \subset \mathbb {P}^1 \times \mathbb {P}^1$ and we denote $\iota _p :p \hookrightarrow \mathbb {P}^1 \times \mathbb {P}^1$ and $\iota _{p_1} :p \hookrightarrow \mathbb {P}^{1} \times \{p_2\} \simeq \mathbb {P}^1$ the inclusion morphisms.

Let ${\overline{M}}_{g,1}(\mathbb {P}^1/\{0\} \cup \{ \infty \}, w; w, w)$ be the moduli space of genus *g* 1-pointed stable maps to $\mathbb {P}^1$, of degree *w*, relative to the divisor $\{0\} \cup \{ \infty \}$, with intersection multiplicities *w* both along $\{0\}$ and $\{\infty \}$. We have an evaluation morphism at the extra marked point

$$\begin{aligned} {\text {ev}}_1 :{\overline{M}}_{g,1}(\mathbb {P}^1/\{0\} \cup \{ \infty \}, w; w, w) \rightarrow \mathbb {P}^1 , \end{aligned}$$

Because an element $(f :C \rightarrow \mathbb {P}^1 \times \mathbb {P}^1)$ of ${\text {ev}}^{-1}(p)$ factors through $\mathbb {P}^1 \times \{p_2\} \simeq \mathbb {P}^1$, we have a natural identification of moduli spaces ${\text {ev}}^{-1}(p)={\text {ev}}_1^{-1}(p)$, but the natural virtual fundamental classes are different. The class $\iota _p^! [{\overline{M}}_{g, \varDelta _V}]^{\mathrm {virt}}$, defined by the refined Gysin homomorphism (see Section 6.2 of [16]), has degree *g* whereas the class $\iota _{p_1}^![{\overline{M}}_{g,1}(\mathbb {P}^1/\{0\} \cup \{ \infty \}, w ; w, w)]^{\mathrm {virt}}$ is of degree

$$\begin{aligned} 2g-2+2w-(w-1)-(w-1)+(1-1)=2g. \end{aligned}$$

The two obstruction theories differ by the bundle whose fiber at

$$\begin{aligned} f :C \rightarrow \mathbb {P}^1 \end{aligned}$$

is $H^1(C, f^*N_{f(C)|\mathbb {P}^1 \times \mathbb {P}^1})$. Because $\beta _{\varDelta _V}^2=0$, the normal bundle $N_{f(C)|\mathbb {P}^1 \times \mathbb {P}^1}$ is trivial of rank one, so the pullback $f^*N_{f(C)|\mathbb {P}^1 \times \mathbb {P}^1}$ is trivial of rank one and the two obstruction theories differ by the dual of the Hodge bundle. Therefore, we have

$$\begin{aligned} \iota _p^! \left[ {\overline{M}}_{g, \varDelta _V}\right] ^{\mathrm {virt}}= c_g(\mathbb {E}^*) \cap \iota _{p_1}^!\left[ {\overline{M}}_{g,1}(\mathbb {P}^1/\{0\} \cup \{ \infty \}, w; w, w)\right] ^{\mathrm {virt}}, \end{aligned}$$

and so

$$\begin{aligned} N^1_{g,V}=\int _{\iota _p^! [{\overline{M}}_{g, \varDelta _V}]^{\mathrm {virt}}} (-1)^g \lambda _g =\int _{\iota _{p_1}^![{\overline{M}}_{g,1}(\mathbb {P}^1/\{0\} \cup \{ \infty \}, w; w, w)]^{\mathrm {virt}}} \lambda _g^2 . \end{aligned}$$

But $\lambda _g^2=0$ for $g>0$, as follows from Mumford’s relation [36]

$$\begin{aligned} c(\mathbb {E})c(\mathbb {E}^*)=1, \end{aligned}$$

and so $N^1_{g,V}=0$ if $g >0$.

If $g=0$, we have $\lambda _0^2=1$, the moduli space is a point, given by the degree *w* map $\mathbb {P}^1 \rightarrow \mathbb {P}^1$ fully ramified over 0 and $\infty$, with trivial automorphism group (there is no non-trivial automorphism of $\mathbb {P}^1$ fixing 0, $\infty$ and the extra marked point), and so

$$\begin{aligned} N^{1,2}_{0,V}=1. \end{aligned}$$

$$\square $$

#### Lemma 15

Let *V* be a bivalent unpointed vertex of ${\tilde{\varGamma }}$ and $w(E_V)$ the common weight of the two edges adjacent to *V*. Then we have

$$\begin{aligned} N^{1,2}_{g,V}=0 \end{aligned}$$

for every $g >0$, and

$$\begin{aligned} N^{1,2}_{0,V}=\frac{1}{w(E_V)} \end{aligned}$$

for $$g=0$$.

#### Proof

The argument is parallel to the one used to prove Lemma 14. The only difference is that the vertex is no longer pointed and the invariant $N_{g,V}^{1,2}$ is defined using the evaluation map at one of the tangency point. The vanishing for $g>0$ still follows from $\lambda _g^2=0$. For $g=0$, the moduli space is a point, given by the degree $w(E_V)$ map $\mathbb {P}^1 \rightarrow \mathbb {P}^1$ fully ramified over 0 and $\infty$, but now with an automorphism group $\mathbb {Z}/w(E_V)$ (the extra marked point in Lemma 14 is no longer there to kill all non-trivial automorphisms). It follows that $N^{1,2}_{0,V}=\frac{1}{w(E_V)}$. $\square$

#### Corollary 16

Let ${\tilde{h}} :{\tilde{\varGamma }} \rightarrow \mathbb {R}^2$ be a parametrized tropical curve in $T_{\varDelta , p}^g$.

- If there exists one bivalent vertex *V* of ${\tilde{\varGamma }}$ with $g(V) \ne 0$, then
  $$\begin{aligned} N^{\varDelta , n}_{g,{\tilde{h}}}=0. \end{aligned}$$
- If $g(V)=0$ for all the bivalent vertices *V* of ${\tilde{\varGamma }}$, then
  $$\begin{aligned} N_{g, {\tilde{h}}}^{\varDelta , n} = \left( \prod _{V \in V^{(3)}({\tilde{\varGamma }})} N_{g(V), V}^{1, 2} \right) \left( \prod _{E \in E_f(\varGamma )} w(E) \right) , \end{aligned}$$
  where the first product is over the trivalent vertices of $\varGamma$ (or ${\tilde{\varGamma }}$), and the second product is over the bounded edges of $\varGamma$ (not ${\tilde{\varGamma }}$).

#### Proof

If ${\tilde{\varGamma }}$ has a bivalent vertex *V* with $g(V)>0$, then, according to Lemmas 14 and 15, we have $N_{g(V),V}^{1,2}=0$ and so $N_{g,{\tilde{h}}}^{\varDelta ,n}=0$ by Proposition 13.

If $g(V)=0$ for all the bivalent vertices *V* of ${\tilde{\varGamma }}$, then, according to Lemma 14, we have $N_{g(V),V}^{1,2}=1$ for all the bivalent pointed vertices *V* of ${\tilde{\varGamma }}$ and according to Lemma 15, we have $N_{g(V),V}^{1,2}=\frac{1}{w(E_V)}$ for all the bivalent unpointed vertices *V* of ${\tilde{\varGamma }}$ . It follows that Proposition 13 can be rewritten

$$\begin{aligned} N_{g,{\tilde{h}}}^{\varDelta ,n} = \left( \prod _{V \in V^{(3)}({\tilde{\varGamma }})} N_{g(V),V}^{1,2} \right) \left( \prod _{V \in V^{(2up)}({\tilde{\varGamma }})} \frac{1}{w(E_V)} \right) \left( \prod _{E \in E_f({\tilde{\varGamma }})} w(E) \right) , \end{aligned}$$

where the first product is over the trivalent vertices of ${\tilde{\varGamma }}$ (which can be naturally identified with the trivalent vertices of $\varGamma$) and the second product is over the bivalent unpointed vertices of ${\tilde{\varGamma }}$. Recalling from Sect. 4.2 that the edges of ${\tilde{\varGamma }}$ are obtained as subdivision of the edges of $\varGamma$ by adding the bivalent unpointed vertices, we have

$$\begin{aligned} \left( \prod _{V \in V^{(2up)}({\tilde{\varGamma }})} \frac{1}{w(E_V)} \right) \left( \prod _{E \in E_f({\tilde{\varGamma }})} w(E) \right) =\prod _{E \in E_f(\varGamma )} w(E) . \end{aligned}$$

$$\square $$

## Proof of the gluing formula

This Section is devoted to the proof of Proposition 13. Part of it is inspired the proof by Chen [13] of the degeneration formula for expanded stable log maps, and the proof by Kim, Lho and Ruddat [27] of the degeneration formula for stable log maps in degenerations along a smooth divisor. In Sect. 7.1, we define a cut morphism. Restricted to some open substack of torically transverse stable maps, we show in Sect. 7.2 that the cut morphism is étale, and in Sect. 7.3, that the cut morphism is compatible with the natural obstruction theories of the pieces. Using in addition Proposition 11 and the results of Sect. 3, we prove a gluing formula in Sect. 7.4. To finish the proof of Proposition 13, we explain in Sect. 7.5 how to organize the glued pieces.

### Cutting

Let ${\tilde{h}} :{\tilde{\varGamma }} \rightarrow \mathbb {R}^2$ be a parametrized tropical curve in $T_{\varDelta , p}^g$. We denote $V^{(2p)}({\tilde{\varGamma }})$ the set of bivalent pointed vertices of ${\tilde{\varGamma }}$ and $V^{(2up)}({\tilde{\varGamma }})$ the set of bivalent unpointed vertices of ${\tilde{\varGamma }}$.

Evaluations ${\text {ev}}_V^E :{\overline{M}}_{g(V), \varDelta _V} \rightarrow D_E$ at the tangency points dual to the bounded edges of ${\tilde{\varGamma }}$ give a morphism

$$\begin{aligned} {\text {ev}}^{(e)} :\prod _{V \in V({\tilde{\varGamma }})}{\overline{M}}_{g(V), \varDelta _V} \rightarrow \prod _{E \in E_f({\tilde{\varGamma }})} (D_E)^2 , \end{aligned}$$

where $D_E$ is the divisor of $X_0$ dual to an edge *E* of ${\tilde{\varGamma }}$.

Evaluations ${\text {ev}}^{(p)}_V :{\overline{M}}_{g(V), \varDelta _V} \rightarrow X_{\varDelta _V}$ at the extra marked points corresponding to the bivalent pointed vertices give a morphism

$$\begin{aligned} {\text {ev}}^{(p)} :\prod _{V \in V({\tilde{\varGamma }})}{\overline{M}}_{g(V), \varDelta _V} \rightarrow \prod _{V \in V^{(2p)}({\tilde{\varGamma }})} X_{\varDelta _V}. \end{aligned}$$

Let

$$\begin{aligned} \delta :\prod _{E \in E_f({\tilde{\varGamma }})} D_E \rightarrow \prod _{E \in E_f({\tilde{\varGamma }})} (D_E)^2 \end{aligned}$$

be the diagonal morphism. Let

$$\begin{aligned} \iota _{P^0} :\left( P^0 = (P_V^0)_{V \in V^{(2p)}({\tilde{\varGamma }})} \right) \hookrightarrow \prod _{V \in V^{(2p)}({\tilde{\varGamma }})} X_{\varDelta _V} , \end{aligned}$$

be the inclusion morphism of $P^0$.

Using the fiber product diagram in the category of stacks 
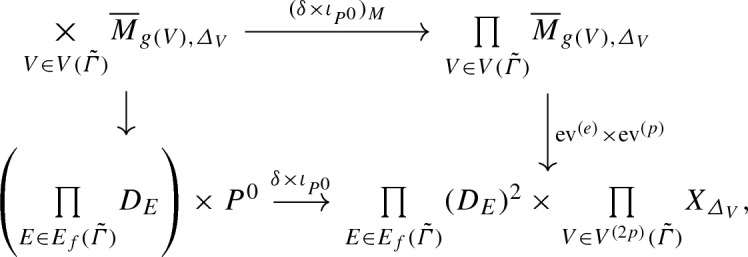
 we define the substack  of $\prod _{V \in V({\tilde{\varGamma }})}{\overline{M}}_{g(V), \varDelta _V}$ consisting of curves whose marked points keeping track of the tangency conditions match over the divisors $D_E$ and whose extra marked points associated to the bivalent pointed vertices map to $P^0$.

#### Lemma 17

Let 
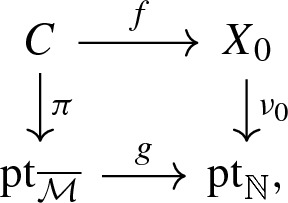
 be a *n*-pointed genus *g* stable log map of type $\varDelta$ passing through $P^0$ and marked by ${\tilde{h}} :{\tilde{\varGamma }} \rightarrow \mathbb {R}^2$, i.e. a point of ${\overline{M}}^{{\tilde{h}},P^0}_{g,n,\varDelta }$. Let 
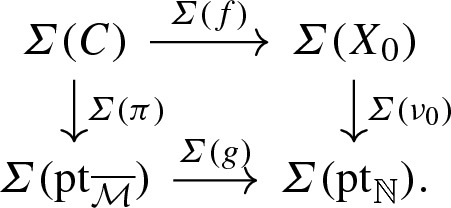
 be its tropicalization. For every $b \in \varSigma (g)^{-1}(1)$, let

$$\begin{aligned} \varSigma (f)_b :\varSigma (C)_b \rightarrow \varSigma (\nu _0)^{-1} (1) \simeq \mathbb {R}^2 \end{aligned}$$

be the fiber of $\varSigma (f)$ over *b*. Let *E* be an edge of $\varGamma$ and let $E_{f,b}$ be the edge of $\varSigma (C)_b$ marked by *E*. Then $\varSigma (f)_b(E_{f,b}) \subset {\tilde{h}}(E)$.

#### Proof

We recalled in Sect. 6 that the connected components of the complement of the bivalent pointed vertices of ${\tilde{\varGamma }}$ are trees with exactly one unbounded edge. We prove Lemma 17 by induction, starting with the edges connected to the bivalent pointed vertices and then we go through each tree following the orientation introduced in Sect. 6.

Let *E* be an edge of ${\tilde{\varGamma }}$ adjacent to a bivalent pointed vertex *V* of ${\tilde{\varGamma }}$. Let $P^0_V \in X_{\varDelta _V}$ be the corresponding marked point. As *f* is marked by ${\tilde{h}}$, we have an ordinary stable map $f_V :C_V \rightarrow X_{\varDelta _V}$, a marked point $x_E$ in $C_V$ such that $f(x_E) \in D_E$ and $f_V(C_V)$ contains $P^0_V$. We can assume that $X_{\varDelta _V}=\mathbb {P}^1 \times \mathbb {P}^1$, $D_E=\{0\} \times \mathbb {P}^1$, $\beta _{\varDelta _V}=w(E)([\mathbb {P}^1] \times [\mathrm {pt}])$, and $P^0_V = (P^0_{V,1}, P^0_{V,2}) \in \mathbb {C}^{*} \times \mathbb {C}^{*} \subset \mathbb {P}^1 \times \mathbb {P}^1$. Then $f_V$ factors through $\mathbb {P}^1 \times \{P^0_{V,2}\}$ and $x_E =(0, P^0_{V,2})$. It follows that $\varSigma (f)_b(E_{f,b}) \subset {\tilde{h}}(E)$.

Let *E* be the outgoing edge of a trivalent vertex of ${\tilde{\varGamma }}$, of ingoing edges $E^1$ and $E^2$. By the induction hypothesis, we know that $\varSigma (f)_b(E^1_{f,b}) \subset {\tilde{h}}(E^1)$ and $\varSigma (f)_b(E^2_{f,b}) \subset {\tilde{h}}(E^2)$. We conclude that $\varSigma (f)_b(E_{f,b}) \subset {\tilde{h}}(E)$ by an application of the balancing condition, as in Proposition 30 (tropical Menelaus theorem) of Mikhalkin [35]. $\square$

For a stable log map 
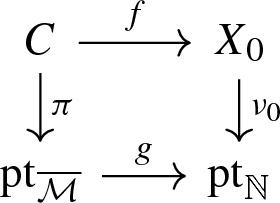
 marked by ${\tilde{h}}$, we have nodes of *C* in correspondence with the bounded edges of ${\tilde{\varGamma }}$. Cutting *C* along these nodes, we obtain a morphismLet us give a precise definition of the cut morphism.^16^ By definition of the marking, for every vertex *V* of ${\tilde{\varGamma }}$, we have an ordinary stable map $f_V :C_V \rightarrow X_{\varDelta _V}$, such that the underlying stable map to *f* is obtained by gluing together the maps $f_V$ along nodes corresponding to the edges of ${\tilde{\varGamma }}$.

We have to give $C_V$ the structure of a log curve, and enhance $f_V$ to a log morphism. In particular, we need to construct a monoid $\overline{\mathcal {M}}_V$.

We fix a point *b* in the interior of $\varSigma (g)^{-1}(1)$. Let $\varSigma (f)_b :\varSigma (C)_b \rightarrow \mathbb {R}^2$ be the corresponding parametrized tropical curve. Let $\varSigma (C)_{V,b}$ be the subgraph of $\varSigma (C)_b$ obtained by taking the vertices of $\varSigma (C)_b$ dual to irreducible components of $C_V$, the edges between them, and considering the edges to other vertices of $\varSigma (C)_b$ as unbounded edges. Let $\varSigma (f)_{V,b}$ be the restriction of $\varSigma (f)_b$ to $\varSigma (C)_{V,b}$. It follows from Lemma 17 that one can view $\varSigma (f)_{V,b}$ as a parametrized tropical curve of type $\varDelta _V$ to the fan of $X_{\varDelta _V}$.

We define $\overline{\mathcal {M}}_V$ as being the monoid whose dual is the monoid of integral points of the moduli space of deformations of $\varSigma (f)_{V,b}$ preserving its combinatorial type.^17^ Let $i_{C_V} :C_V \rightarrow C$ and $i_{X_{\varDelta _V}} :X_{\varDelta _V} \rightarrow X_0$ be the inclusion morphisms of ordinary (not log) schemes. The parametrized tropical curves $\varSigma (f)_V$ encode a sheaf of monoids $\overline{\mathcal {M}}_{C_V}$ and a map $f_V^{-1} \overline{\mathcal {M}}_{X_{\varDelta _V}} \rightarrow \overline{\mathcal {M}}_{C_V}$. We define a log structure on $C_V$ by

$$\begin{aligned} \mathcal {M}_{C_V} =\overline{\mathcal {M}}_{C_V} \times _{i_{C_V}^{-1} \overline{\mathcal {M}}_C} i_{C_V}^{-1} \mathcal {M}_C . \end{aligned}$$

The natural diagram 
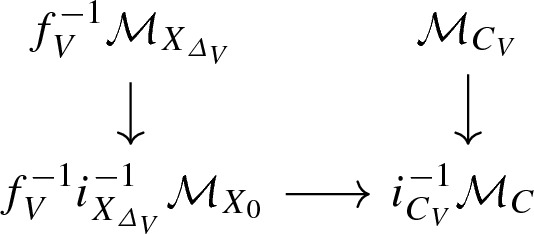
 can be uniquely completed, by restriction, with a map

$$\begin{aligned} f_V^{-1} \mathcal {M}_{X_{\varDelta _V}} \rightarrow \mathcal {M}_{C_V} \end{aligned}$$

compatible with $f_V^{-1}\overline{\mathcal {M}}_{X_{\varDelta _V}} \rightarrow \overline{\mathcal {M}}_{C_V}$. This defines a log enhancement of $f_V$ and finishes the construction of the cut morphism.

**Remark** If one considers a general log smooth degeneration and if one applies the decomposition formula, it is in general impossible to write the contribution of a tropical curves in terms of log Gromov–Witten invariants attached to the vertices. This is already clear at the tropical level. The theory of punctured invariants developed by Abramovich, Chen, Gross, Siebert in [4] is the correct extension of log Gromov–Witten theory which is needed in order to be able to write down a general gluing formula. In our present case, the Nishinou–Siebert toric degeneration is extremely special because it has been constructed knowing a priori the relevant tropical curves. It follows from Lemma 17 that we always cut edges contained in an edge of the polyhedral decomposition, and so we don’t have to consider punctured invariants.

### Counting log structures

We say that a map to $X_0$ is torically transverse if its image does not contain any of the torus fixed points of the toric components $X_{\varDelta _V}$. In other words, its corestriction to each toric surface $X_{\varDelta _V}$ is torically transverse in the sense of Sect. 5.

Let ${\overline{M}}_{g, n, \varDelta }^{{\tilde{h}},P^0, \circ }$ be the open locus of ${\overline{M}}_{g, n, \varDelta }^{{\tilde{h}},P^0}$ formed by the torically transverse stable log maps to $X_0$, and for every vertex *V* of ${\tilde{\varGamma }}$, let ${\overline{M}}_{g(V), \varDelta _V}^\circ$ be the open locus of ${\overline{M}}_{g(V), \varDelta _V}$ formed by the torically transverse stable log maps to $X_{\varDelta _V}$. The morphism cut restricts to a morphism

#### Proposition 18

The morphismis étale of degree

$$\begin{aligned} \prod _{E \in E_f({\tilde{\varGamma }})} w(E) , \end{aligned}$$

where the product is over the bounded edges of ${\tilde{\varGamma }}$.

#### Proof

Let . We have to glue the stable log maps $f_V$ together. Because we are assuming that the maps $f_V$ are torically transverse, the image in $X_0$ by $f_V$ of the curves $C_V$ is away from the torus fixed points of the components $X_{\varDelta _V}$. The gluing operation corresponding to the bounded edge *E* of ${\tilde{\varGamma }}$ happens entirely along the torus $\mathbb {C}^{*}$ contained in the divisor $D_E$.

It follows that it is enough to study the following local model. Denote $\ell \,{:}{=}\, \ell (E) w(E)$, where $\ell (E)$ is the length of *E* and *w*(*E*) the weight of *E*. Let $X_E$ be the toric variety ${\text {Spec}}\,\mathbb {C}[x,y,u^{\pm }, t]/(xy=t^\ell )$, equipped with a morphism $\nu _E :X_E \rightarrow \mathbb {C}$ given by the coordinate *t*. Using the natural toric divisorial log structures on $X_E$ and $\mathbb {C}$, we define by restriction a log structure on the special fiber $X_{0,E} \,{:}{=}\, \nu _E^{-1}(0)$ and a log smooth morphism to the standard log point $\nu _{0,E} :X_{0,E} \rightarrow \mathrm {pt}_{\mathbb {N}}$. The scheme underlying $X_{0,E}$ has two irreducible components, $X_{1,E} \,{:}{=}\, \mathbb {C}_x \times \mathbb {C}^{*}_u$ and $X_{2,E} \,{:}{=}\, \mathbb {C}_y \times \mathbb {C}^{*}_u$, glued along the smooth divisor $D_E^\circ \,{:}{=}\, \mathbb {C}^{*}_u$. We endow $X_{1,E}$ and $X_{2,E}$ with their toric divisorial log structures.

Let $f_1 :C_1/\mathrm {pt}_{\overline{\mathcal {M}}_1} \rightarrow X_{1,E}$ be the restriction to $X_{1,E}$ of a torically transverse stable log map to some toric compactification of $X_{1,E}$, with one point $p_1$ of tangency order *w*(*E*) along $D_E$, and let $f_2 :C_2/\mathrm {pt}_{\overline{\mathcal {M}}_2} \rightarrow X_{2,E}$ be the restriction to $X_{2,E}$ of a torically transverse stable log map to some toric compactification of $X_{2,E}$, with one point $p_2$ of tangency order *w*(*E*) along $D_E$. We assume that $f(p_1)=f(p_2)$ and so we can glue the underlying maps $\underline{f}_1 :\underline{C}_1 \rightarrow \underline{X}_{1,E}$ and $\underline{f}_2 :\underline{C}_2 \rightarrow \underline{X}_{2,E}$ to obtain a map $\underline{f} :\underline{C} \rightarrow \underline{X}_{0,E}$ where $\underline{C}$ is the curve obtained from $\underline{C}_1$ and $\underline{C}_2$ by identification of $p_1$ and $p_2$. We denote *p* the corresponding node of $\underline{C}$. We have to show that there are *w*(*E*) ways to lift this map to a log map in a way compatible with the log maps $f_1$ and $f_2$ and with the basic condition. If $C_1$ and $C_2$ had no component contracted to $f(p) \in D_E^\circ$, this would follow from Proposition 7.1 of Nishinou, Siebert [38]. But we allow contracted components, so we have to present a variant of the proof of Proposition 7.1 of [38].

We first give a tropical description of the relevant objects. The tropicalization of $X_{0,E}$ is the cone $\varSigma (X_{0,E}) ={\text {Hom}}(\overline{\mathcal {M}}_{X_{0,E},f(p)}, \mathbb {R}_{\geqslant 0})$. It is the fan of $X_{E}$, a two-dimensional cone generated by rays $\rho _1$ and $\rho _2$ dual to the divisors $X_{1,E}$ and $X_{2,E}$. The toric description $X_E={\text {Spec}}\,\mathbb {C}[x,y,u^{\pm },t]/(xy=t^\ell )$ defines a natural chart for the log structure of $X_{0,E}$. Denote $s_x, s_y, s_t$ the corresponding elements of $\mathcal {M}_{X_{0,E}, f(p)}$ and $\overline{s}_x, \overline{s}_y, \overline{s}_t$ their projections in $\overline{\mathcal {M}}_{X_{0,E}, f(p)}$. We have $s_x s_y= s_t^\ell$. Seeing elements of $\overline{\mathcal {M}}_{X_{0,E}, f(p)}$ as functions on $\varSigma (X_{0,E})$, we have $\rho _1=\overline{s}_y^{-1}(0)$, $\rho _2=\overline{s}_x^{-1}(0)$ and $\overline{s}_t :\varSigma (X_{0,E}) \rightarrow \mathbb {R}_{\geqslant 0}$ is the tropicalization of the projection $X_{0,E} \rightarrow \mathrm {pt}_{\mathbb {N}}$. Level sets $\overline{s}_t^{-1}(c)$ are line segments $[P_1,P_2]$ in $\varSigma (X_{0,E})$, connecting a point $P_1$ of $\rho _1$ to a point $P_2$ of $\rho _2$, of length $\ell c$.

Denote $\underline{C}_{1,E}$ and $\underline{C}_{2,E}$ the irreducible components of $\underline{C}_1$ and $\underline{C}_{2}$ containing $p_1$ and $p_2$ respectively. We can see them as the two irreducible components of $\underline{C}$ meeting at the node *p*. Fix $j=1$ or $j=2$. The tropicalization of $C_j/\mathrm {pt}_{\overline{\mathcal {M}}_j}$ is a family $\varSigma (C_j)$ of tropical curves $\varSigma (C_j)_b$ parametrized by $b \in \varSigma (\mathrm {pt}_{\overline{\mathcal {M}}_j}) ={\text {Hom}}(\overline{\mathcal {M}}_j,\mathbb {R}_{\geqslant 0})$. Let $V_{j,E}$ be the vertex of these tropical curves dual to the irreducible component $\underline{C}_{j,E}$. The image $\varSigma (f_j)(V_{j,E})$ of $V_{j,E}$ by the tropicalization $\varSigma (f_j)$ of $f_j$ is a point in the tropicalization $\varSigma (X_{j,E})=\mathbb {R}_{\geqslant 0}$. This induces a map ${\text {Hom}}(\overline{\mathcal {M}}_j,\mathbb {R}_{\geqslant 0}) \rightarrow \mathbb {R}_{\geqslant 0}$ defined by an element $v_j \in \overline{\mathcal {M}}_j$. The component $\underline{C}_{j,E}$ is contracted by $f_j$ onto $f_j(p_j)$ if and only if $v_j \ne 0$. In other words, $v_j$ is the measure according to the log structures of “how” $\underline{C}_{j,E}$ is contracted by $f_j$. The marked point $p_j$ on $C_{j,E}$ defines an unbounded edge $E_j$, of weight *w*(*E*), whose image by $\varSigma (f_j)$ is the unbounded interval $[\varSigma (f_j)(V_{j,E}),+\infty ) \subset \varSigma (X_{j,E})=\mathbb {R}_{\geqslant 0}$.

We explain now the gluing at the tropical level. Let $j=1$ or $j=2$. Let $[0,\ell _j] \subset \varSigma (X_{j,E})=\mathbb {R}_{\geqslant 0}$ be an interval. If *c* is a large enough positive real number, we denote $\varphi _c^j :[0,\ell _j] \hookrightarrow \overline{s}_t^{-1}(c)=[P_1,P_2]$ the linear inclusion such that $\varphi _c^j(0)=P_j$ and $\varphi _c^j ([0,\ell _j])$ is a subinterval of $[P_1,P_2]$ of length $\ell _j$. Let $b_j \in \varSigma (\mathrm {pt}_{\overline{\mathcal {M}}_j})$. There exists $\ell _j$ large enough such that all images by $\varSigma (f_j)$ of vertices of $\varSigma (f_j)_{b_j}$ are contained in $[0,\ell _j] \subset \varSigma (X_{j,E})=\mathbb {R}_{\geqslant 0}$.

For *c* large enough, the line segments $\varphi _c^1([0,\ell _1])$ and $\varphi _c^2([0,\ell _2])$ are disjoint. We have

$$\begin{aligned}{}[P_1,P_2]= & {} \left[ P_1,\varphi _c^1(\varSigma (f_1)(V_1))) \right. \\&\cup \left[ \varphi _c^1(\varSigma (f_1)(V_1)), \varphi _c^2(\varSigma (f_2)(V_2))\right] \\&\left. \cup (\varphi _c^2(\varSigma (f_2)(V_2)),P_2\right] . \end{aligned}$$

We construct a new tropical curve $\varSigma _{b_1,b_2,c}$ by removing the unbounded edges $E_1$ and $E_2$ of $\varSigma (f_1)_{b_1}$ and $\varSigma (f_2)_{b_2}$, and gluing the remaining curves by an edge *F* connecting $V_{1,E}$ and $V_{2,E}$, of weight *w*(*E*), and length $\frac{1}{w(E)}$ times the length of the line segment $[\varphi ^1_c(\varSigma (f_1)(V_1)) ,\varphi ^2_c(\varSigma (f_2)(V_2))]$. We construct a tropical map $\varSigma _{b_1,b_2,c} \rightarrow \varSigma (X_{0,E})$ using $\varSigma (f_1)_{b_1}$, $\varSigma (f_2)_{b_2}$ and mapping the edge *F* to $[\varphi ^1_c(\varSigma (f_1)(V_1)), \varphi ^2_c(\varSigma (f_2)(V_2))]$. We define $\overline{\mathcal {M}}$ as being the monoid whose dual is the monoid of integral points of the moduli space of deformations of these tropical maps.

We have $$\overline{\mathcal {M}} =\overline{\mathcal {M}}_1 \oplus \overline{\mathcal {M}}_2 \oplus \mathbb {N}$$. The element $$(0,0,1) \in \overline{\mathcal {M}}$$ defines the function on the moduli space of tropical curves $$\varSigma (\mathrm {pt}_{\overline{\mathcal {M}}}) ={\text {Hom}}(\overline{\mathcal {M}}, \mathbb {R}_{\geqslant 0})$$ given by the length of the gluing edge *F*. The function given by $$\frac{1}{\ell }$$ times the length of the line segment $$[P_1,P_2]$$ defines an element $$\overline{s}_t^{\overline{\mathcal {M}}} \in \overline{\mathcal {M}}$$. The morphism of monoids $$\mathbb {N}\rightarrow \overline{\mathcal {M}}$$, $$1 \mapsto \overline{s}_t^{\overline{\mathcal {M}}}$$, induces a map $$g :\mathrm {pt}_{\overline{\mathcal {M}}} \rightarrow \mathrm {pt}_{\mathbb {N}}$$. The decomposition of $$ [P_1,P_2]$$ into the three intervals $$[P_1,\varphi _c^1(\varSigma (f_1)(V_1)))$$, $$ [\varphi _c^1(\varSigma (f_1)(V_1)), \varphi _c^2(\varSigma (f_2)(V_2))]$$ and $$(\varphi _c^2(\varSigma (f_2)(V_2)),P_2]$$, implies the relation

$$\begin{aligned} \ell \, \overline{s}_t^{\overline{\mathcal {M}}} = (v_1,0,0) + (0,0,w(E))+(0,v_2,0) \end{aligned}$$

in $$\overline{\mathcal {M}} =\overline{\mathcal {M}}_1 \oplus \overline{\mathcal {M}}_2 \oplus \mathbb {N}$$. 
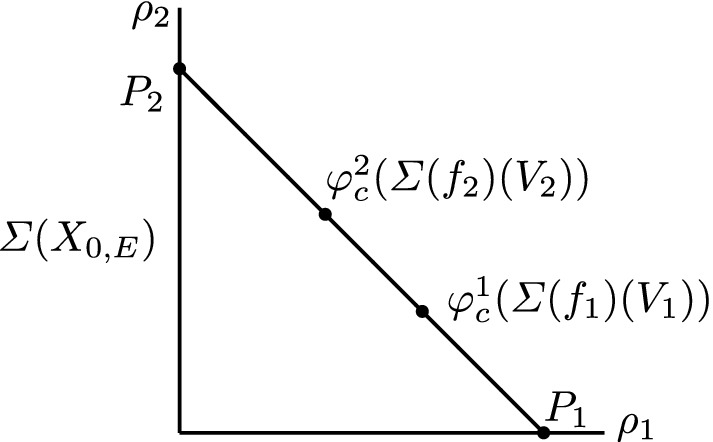


From the tropical description of the gluing and from the fact that we want to obtain a basic log map, we find that there is a unique structure of log smooth curve $C/\mathrm {pt}_{\overline{\mathcal {M}}}$ compatible with the structures of log smooth curves on $C_1$ and $C_2$. As *p* is a node of *C*, we have for the ghost sheaf of *C* at *p*: $\overline{\mathcal {M}}_{C,p}=\overline{\mathcal {M}} \oplus _{\mathbb {N}} \mathbb {N}^2$, with $\mathbb {N}\rightarrow \mathbb {N}^2$, $1 \mapsto (1,1)$, and $\mathbb {N}\rightarrow \overline{\mathcal {M}}=\overline{\mathcal {M}}_1 \oplus \overline{\mathcal {M}}_2 \oplus \mathbb {N}$, $1 \mapsto \rho _p=(0,0,1)$.

It remains to lift $\underline{f} :\underline{C} \rightarrow \underline{X}_{0,E}$ to a log map $f :C \rightarrow X_{0,E}$ such that the diagram 
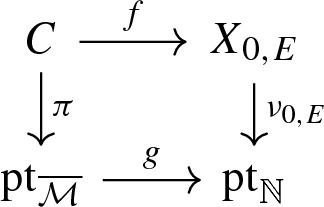
 commutes. The restriction of *f* to $C_j/\mathrm {pt}_{\overline{\mathcal {M}}_j}$ has to coincide with $f_j$, for $j=1$ and $j=2$. It follows from the explicit description of $\overline{\mathcal {M}}$ and $\overline{\mathcal {M}}_C$ that such *f* exists and is unique away from the node *p*.

It follows from the tropical description of the gluing that at the ghost sheaves level, *f* at *p* is given by

$$\begin{aligned} \overline{f}^{\flat } :\overline{\mathcal {M}}_{X_{0,E},f(p)} \rightarrow \overline{\mathcal {M}}_{C,p} =\overline{\mathcal {M}} \oplus _{\mathbb {N}} \mathbb {N}^2 =(\overline{\mathcal {M}}_1 \oplus \overline{\mathcal {M}}_2 \oplus \mathbb {N}) \oplus _{\mathbb {N}} \mathbb {N}^2 \end{aligned}$$

$$\begin{aligned} \overline{s}_x&\mapsto ((v_1,0,0), (w(E),0)) \\ \overline{s}_y&\mapsto ((0,v_2,0), (0,w(E))) \\ \overline{s}_t&\mapsto \overline{\pi }^{\flat }(\overline{s}_t^{\overline{\mathcal {M}}}) =(\overline{s}_t^{\overline{\mathcal {M}}},(0,0)). \end{aligned}$$

The relation $\ell \, \overline{s}_t^{\overline{\mathcal {M}}} = (v_1,v_2,w(E))$ in $\overline{\mathcal {M}} =\overline{\mathcal {M}}_1 \oplus \overline{\mathcal {M}}_2 \oplus \mathbb {N}$ implies that

$$\begin{aligned} \overline{f}^{\flat }(\overline{s}_x) +\overline{f}^{\flat }(\overline{s}_y)&=((v_1,v_2,0),(w(E),w(E))) =((v_1,v_2,w(E)),(0,0)) \\&= \overline{f}^{\flat } (\ell \overline{s}_t^{\overline{\mathcal {M}}}) , \end{aligned}$$

and so that this map is indeed well-defined.

The log maps $f_1 :C_1/\mathrm {pt}_{\overline{\mathcal {M}}_1} \rightarrow X_{1,E}$ and $f_2 :C_2/\mathrm {pt}_{\overline{\mathcal {M}}_2} \rightarrow X_{2,E}$ define morphisms

$$\begin{aligned} f_1^{\flat } :\mathcal {M}_{X_{1,E},f(p_1)} \rightarrow \mathcal {M}_{C_1,p_1} ,\ \end{aligned}$$

and

$$\begin{aligned} f_2^{\flat } :\mathcal {M}_{X_{2,E},f(p_2)} \rightarrow \mathcal {M}_{C_2,p_2} . \end{aligned}$$

For $j=1$ or $j=2$, let $\overline{\mathcal {M}}_j \oplus \mathbb {N}\rightarrow \mathcal {O}_{C_j,p_j}$ be a chart of the log structure of $C_j$ at $p_j$. This realizes $\mathcal {M}_{C_j,p_j}$ as a quotient of $(\overline{\mathcal {M}}_j \oplus \mathbb {N}) \oplus \mathcal {O}_{C,p}^{*}$. Denote $s_{j,m} \in \mathcal {M}_{C_j,p_j}$ the image of (*m*, 1) for $m \in \overline{\mathcal {M}}_j \oplus \mathbb {N}$.

We fix a coordinate *u* on $C_1$ near $p_1$ such that

$$\begin{aligned} f_1^{\flat }(s_x)=s_{1,(v_1,0)} u^{w(E)} \end{aligned}$$

and a coordinate *v* on $C_2$ near $p_2$ such that

$$\begin{aligned} f_2^{\flat }(s_y)=s_{2,(v_2,0)} v^{w(E)}. \end{aligned}$$

We are trying to define some $f^{\flat } :\mathcal {M}_{X_{0,E},f(p)} \rightarrow \mathcal {M}_{C,p}$, lift of $\overline{f}^{\flat }$, compatible with $f_1^{\flat }$ and $f_2^{\flat }$. For every $\zeta$ a *w*(*E*)-th root of unity, the mapdefines a chart for the log structure of *C* at *p*. This realizes $\mathcal {M}_{C,p}$ as a quotient of $(\overline{\mathcal {M}} \oplus _{\mathbb {N}} \mathbb {N}^2) \oplus \mathcal {O}_{C,p}^{*}$. Denote $s_m^{\zeta } \in \mathcal {M}_{C,p}$ the image of (*m*, 1) for $m \in \overline{\mathcal {M}} \oplus _{\mathbb {N}} \mathbb {N}^2$. Remark that $s^\zeta _{((v_1,0,0),(0,0))}$, $s^\zeta _{((0,v_2,0),(0,0))}$ and $s^\zeta _{((0,0,0),(1,1))}$ are independent of $\zeta$ and we denote them simply as $s_{((v_1,0,0),(0,0))}$, $s_{((0,v_2,0),(0,0))}$ and $s_{((0,0,0),(1,1))}$.

Then

$$\begin{aligned} f^{\flat , \zeta } :\mathcal {M}_{X_{0,E},f(p)} \rightarrow \mathcal {M}_{C,p} \end{aligned}$$

$$\begin{aligned} s_x&\mapsto s^\zeta _{((v_1,0,0),(w(E),0))} \\ s_y&\mapsto s^\zeta _{((0,v_2,0),(0,w(E)))}\\ s_t&\mapsto \pi ^{\flat }((\overline{s}_t^{\overline{\mathcal {M}}},1)) \end{aligned}$$

is a lift of $\overline{f}^{\flat }$, compatible with $f_1^{\flat }$ and $f_2^{\flat }$.

Assume that $f^{\flat , \zeta } \simeq f^{\flat ,\zeta '}$ for $\zeta$ and $\zeta '$ two *w*(*E*)-th roots of unity. It follows from the compatibility with $f_1^{\flat }$ and $f_2^{\flat }$ that there exists $\varphi _1 \in \mathcal {O}_{C,p}^{*}$ and $\varphi _2 \in \mathcal {O}_{C,p}^{*}$ such that $s^{\zeta '}_{((0,0,0),(1,0))} =\varphi _1 s^{\zeta }_{((0,0,0),(0,1))}$ and $s^{\zeta '}_{((0,0,0),(0,1))} =\varphi _2 s^{\zeta }_{((0,0,0),(0,1))}$. It follows from the definition of the charts that $\varphi _1=\zeta ' \zeta ^{-1}$ in $\mathcal {O}_{C_1,p_1}$ and $\varphi _2=1$ in $\mathcal {O}_{C_2,p_2}$. Compatibility with $\mathrm {pt}_{\overline{\mathcal {M}}} \rightarrow \mathrm {pt}_{\mathbb {N}}$ implies that $\varphi _1 \varphi _2=1$. This implies that $\varphi _1=\varphi _2=1$ and $\zeta =\zeta '$.

It remains to show that any $f^{\flat }$, lift of $\overline{f}^{\flat }$ compatible with $f_1^{\flat }$ and $f_2^{\flat }$, is of the form $f^{\flat , \zeta }$ for some $\zeta$ a *w*(*E*)-th root of unity. For such $f^{\flat }$, there exists unique $s'_{(1,0)} \in \mathcal {M}_{C,p}$ and $s'_{(0,1)} \in \mathcal {M}_{C,p}$ such that $\alpha _C(s'_{(1,0)})=u$, $\alpha _C(s'_{(0,1)})=v$, and $f^{\flat }(s_x)=s_{((v_1,0,0),(0,0))} (s'_{(1,0)})^{w(E)}$ and $f^{\flat }(s_y)=s_{((0,v_2,0),(0,0))} (s'_{(0,1)})^{w(E)}$. From $s_x s_y =s_t^\ell$, we get $(s'_{(1,0)}s'_{(0,1)})^{w(E)} =s_{((0,0,0),(1,1))}^{w(E)}$ and so $s'_{(1,0)}s'_{(0,1)}=\zeta ^{-1} s_{((0,0,0),(1,1))}$ for some $\zeta$ a *w*(*E*)-th root of unity. It is now easy to check that $s'_{(1,0)} =\zeta ^{-1} s^{\zeta }_{((0,0,0),(1,0))}$, $s'_{(0,1)} = s^{\zeta }_{((0,0,0),(0,1))}$ and $f^\flat = f^{\flat , \zeta }$. $\square$

**Remarks**

- When $v_1=v_2=0$, i.e. when the components $C_{1,E}$ and $C_{2,E}$ are not contracted, the above proof reduces to the proof of Proposition 7.1 of [38] (see also the proof of Proposition 4.23 of [21]). In general, log geometry remembers enough information about the contracted components, such as $v_1$ and $v_2$, to make possible a parallel argument.
- The gluing of stable log maps along a smooth divisor is discussed in Section 6 of [27], proving the degeneration formula along a smooth divisor. In the above proof, we only have to glue along one edge connecting two vertices. In Section 6 of [27], further work is required to deal with pair of vertices connected by several edges.

### Comparing obstruction theories

As in the previous Sect. 7.2, let ${\overline{M}}_{g, n, \varDelta }^{{\tilde{h}}, P^0,\circ }$ be the open locus of ${\overline{M}}_{g, n, \varDelta }^{{\tilde{h}},P^0}$ formed by the torically transverse stable log maps to $X_0$, and for every vertex *V* of ${\tilde{\varGamma }}$, let ${\overline{M}}_{g(V), \varDelta _V}^\circ$ be the open locus of ${\overline{M}}_{g(V), \varDelta _V}$ formed by the torically transverse stable log maps to $X_{\varDelta _V}$. The morphism cut restricts to a morphismThe goal of the present Section is to use the morphism $\mathrm {cut}^\circ$ to compare the virtual classes $[{\overline{M}}_{g, n, \varDelta }^{{\tilde{h}},P^0,\circ }]^{\mathrm {virt}}$ and $[{\overline{M}}^\circ _{g(V), \varDelta _V}]^{\mathrm {virt}}$, which are obtained by restricting the virtual classes $[{\overline{M}}_{g, n, \varDelta }^{{\tilde{h}},P^0}]^{\mathrm {virt}}$ and $[{\overline{M}}_{g(V), \varDelta _V}]^{\mathrm {virt}}$ to the open loci of torically transverse stable log maps.

Recall that $X_0=\nu ^{-1}(0)$, where $\nu :X_{\mathcal {P}_{\varDelta ,n}} \rightarrow \mathbb {A}^1$. Following Section 4.1 of [3], we define $\mathcal {X}_0 \,{:}{=}\, \mathcal {A}_X \times _{\mathcal {A}_{\mathbb {A}^1}} \{0\}$, where $\mathcal {A}_X$ and $\mathcal {A}_{\mathbb {A}^1}$ are Artin fans, see Section 2.2 of [3]. It is an algebraic log stack over $\mathrm {pt}_{\mathbb {N}}$. There is a natural morphism $X_0 \rightarrow \mathcal {X}_0$.

Following Section 4.5 of [3], let $\mathfrak {M}_{g,n,\varDelta }^{{\tilde{h}}}$ be the stack of *n*-pointed genus *g* prestable basic log maps to $\mathcal {X}_0/\mathrm {pt}_{\mathbb {N}}$ marked by ${\tilde{h}}$ and of type $\varDelta$. There is a natural morphism of stacks ${\overline{M}}^{{\tilde{h}},P^0}_{g,n,\varDelta } \rightarrow \mathfrak {M}_{g,n,\varDelta }^{{\tilde{h}}}.$ Let $\pi :\mathcal {C}\rightarrow {\overline{M}}_{g,n,\varDelta }^{{\tilde{h}},P^0}$ be the universal curve and let $f :\mathcal {C}\rightarrow X_0/ \mathrm {pt}_{\mathbb {N}}$ be the universal stable log map. According to Proposition 4.7.1 and Section 6.3.2 of [3], the virtual fundamental class $[{\overline{M}}_{g,n,\varDelta }^{{\tilde{h}},P^0}]^{\mathrm {virt}}$ is defined by $\mathbf {E}$, the cone of the morphism $({\text {ev}}^{(p)})^{*} L_{\iota _{P^0}}[-1] \rightarrow (R\pi _{*} f^{*} T_{X_0| \mathcal {X}_0})^\vee$, seen as a perfect obstruction theory relative to $\mathfrak {M}_{g,n,\varDelta }^{{\tilde{h}}}$. Here, $T_{X_0|\mathcal {X}_0}$ is the relative log tangent bundle, and $L_{\iota _{P^0}}=\oplus _{V \in V^{(2p)}({\tilde{\varGamma }})} (T_{X_{\varDelta _V}}|_{P^0_V})^\vee [1]$ is the cotangent complex of $\iota _{P^0}$. As $\mathcal {X}_0$ is log étale over $\mathrm {pt}_\mathbb {N}$, we have $T_{X_0|\mathcal {X}_0} =T_{X_0|\mathrm {pt}_{\mathbb {N}}}$. We denote $\mathbf {E}^\circ$ the restriction of $\mathbf {E}$ to the open locus ${\overline{M}}_{g,n,\varDelta }^{{\tilde{h}},P^0, \circ }$ of torically transverse stable log maps.

For every vertex *V* of ${\tilde{\varGamma }}$, let $\pi _V :\mathcal {C}_V \rightarrow {\overline{M}}_{g(V), \varDelta _V}$ be the universal curve and let $f_V :\mathcal {C}_V \rightarrow X_{\varDelta _V}$ be the universal stable log map. Let $\mathcal {A}_{X_{\varDelta _V}}$ be the Artin fan of $X_{\varDelta _V}$ and let $\mathfrak {M}_{g(V),\varDelta _V}$ be the stack of prestable basic log maps to $\mathcal {A}_{X_{\varDelta _V}}$, of genus *g*(*V*) and of type $\varDelta _V$. There is a natural morphism of stacks ${\overline{M}}_{g(V),\varDelta _V} \rightarrow \mathfrak {M}_{g(V),\varDelta _V}$. According to Section 6.1 of [6], the virtual fundamental class $[{\overline{M}}_{g(V), \varDelta _V}]^{\mathrm {virt}}$ is defined by $(R (\pi _V)_{*} f_V^{*} T_{X_{\varDelta _V}})^\vee$, seen as a perfect obstruction theory relative to $\mathfrak {M}_{g(V),\varDelta _V}$. Here, $T_{X_{\varDelta _V}}$ is the log tangent bundle.

Recall that  is defined by the fiber product diagram 
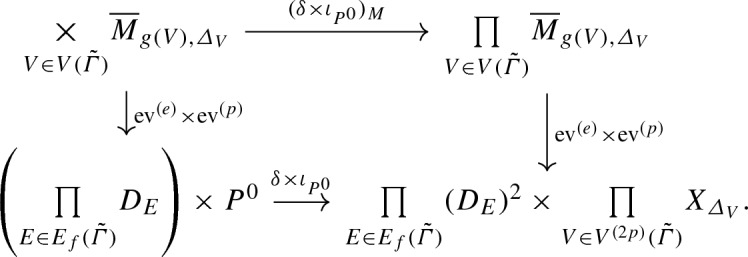


We compare the deformation theory of the individual stable log maps $f_V$ and the deformation theory of the stable log maps $f_V$ constrained to match at the gluing nodes. Let $\mathbf {F}$ be the cone of the natural morphism

$$\begin{aligned} (\text {ev}^{(e)} \times \text {ev}^{(p)})^{*} L_{\delta \times \iota _{P^0}} [-1] \rightarrow (\delta \times \iota _{P^0})_M^{*} \left( \underset{V \in V({\tilde{\varGamma }})}{{{{\boxtimes }}}} (R(\pi _V)_{*} f_V^{*} T_{X_{\varDelta _V}})^\vee \right) , \end{aligned}$$

where $L_{\delta \times \iota _{P^0}}$ is the cotangent complex of the morphism $\delta \times \iota _{P^0}$. It defines a perfect obstruction theory on  relative to $\underset{V \in V({\tilde{\varGamma }})}{\prod } \mathfrak {M}_{g(V), \varDelta _V}$, whose corresponding virtual fundamental class is, using Proposition 5.10 of [7],

$$\begin{aligned} (\delta \times \iota _{P^0})^! \prod _{V \in V({\tilde{\varGamma }})} [{\overline{M}}_{g(V),\varDelta _V}]^{\mathrm {virt}}, \end{aligned}$$

where $(\delta \times \iota _{P^0})^!$ is the refined Gysin homomorphism (see Section 6.2 of [16]). We denote $\mathbf {F}^\circ$ the restriction of $\mathbf {F}$ to the open locus  of torically transverse stable log maps.

The cut operation naturally extends to prestable log maps to $\mathcal {X}_0/\mathrm {pt}_{\mathbb {N}}$ marked by ${\tilde{h}}$, and so we have a commutative diagram 
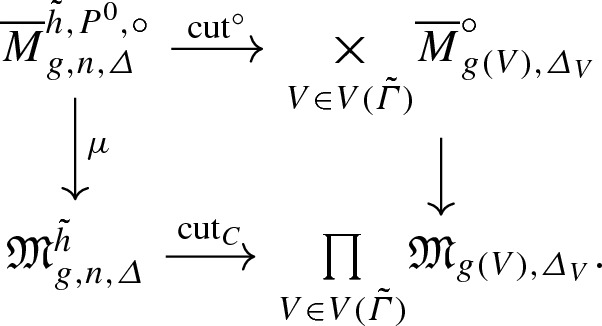
 By Proposition 18, the morphism $\mathrm {cut}^\circ$ is étale and so $(\mathrm {cut}^\circ )^{*} \mathbf {F}^\circ$ defines a perfect obstruction theory on ${\overline{M}}_{g,n,\varDelta }^{{\tilde{h}},P^0,\circ }$ relative to $\underset{V \in V({\tilde{\varGamma }})}{\prod } \mathfrak {M}_{g(V), \varDelta _V}$.

The maps ${\overline{M}}_{g,n,\varDelta }^{{\tilde{h}},P^0,\circ } \xrightarrow {\mu } \mathfrak {M}_{g,n,\varDelta }^{{\tilde{h}}} (\mathcal {X}_0 /\mathrm {pt}_{\mathbb {N}}) \xrightarrow {\mathrm {cut}_C} \underset{V \in V({\tilde{\varGamma }})}{\prod } \mathfrak {M}_{g(V), \varDelta _V}$ define an exact triangle of cotangent complexes 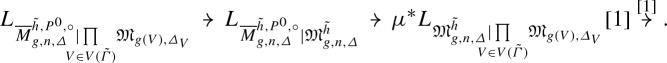 Adding the perfect obstruction theories $(\mathrm {cut}^\circ )^{*} \mathbf {F}^\circ$ and $\mathbf {E}^\circ$, we get a diagram 
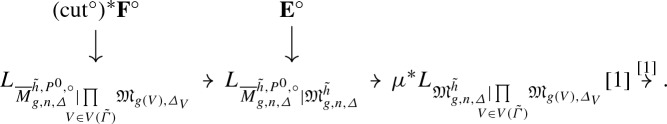


#### Proposition 19

The above diagram can be completed into a morphism of exact triangles 
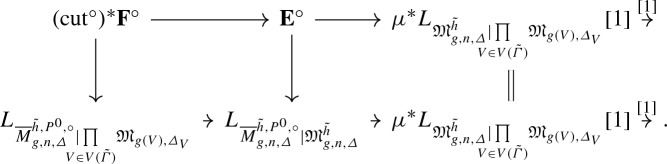


#### Proof

Denote $X_0^\circ$, $X_{\varDelta _V}^\circ$, $D_E^\circ$ the objects obtained from $X_0$, $X_{\varDelta _V}$, $D_E$ by removing the torus fixed points of the toric surfaces $X_{\varDelta _V}$. Denote $\iota _{X_{\varDelta _V}^\circ }$ the inclusion morphism of $X_{\varDelta _V}^\circ$ in $X_0^\circ$.

If *E* is a bounded edge of ${\tilde{\varGamma }}$, we denote $V_E^1$ and $V_E^2$ the two vertices of *E*. Let $\mathcal {F}$ be the sheaf on the universal curve $\mathcal {C}|_{{\overline{M}}_{g,n,\varDelta }^{{\tilde{h}},P^0,\circ }}$ defined as the kernel of

$$\begin{aligned}&\bigoplus _{V \in V({\tilde{\varGamma }})} f^{*}(\iota _{X_{\varDelta _V}^\circ })_* T_{X_{\varDelta _V}^\circ } \rightarrow \bigoplus _{E \in E_f({\tilde{\varGamma }})} (\iota _{E})_* ({\text {ev}}^E)^* T_{D_E^\circ }\\&(s_V)_V \mapsto (s_{V_E^1}|_{D_E^\circ } -s_{V_E^2}|_{D_E^\circ })_E , \end{aligned}$$

where ${\text {ev}}^E$ is the evaluation at the node $p_E$ dual to *E*, and $\iota _E$ the section of $\mathcal {C}$ given by $p_E$. It follows from the exact triangle obtained by applying $R \pi _*$ to the short exact sequence defining $\mathcal {F}$ and from $L_{\delta }=\oplus _{E \in E_f({\tilde{\varGamma }})}T_{D_E}^\vee [1]$ that $(\mathrm {cut}^\circ )^* \mathbf {F}^\circ$ is given by the cone of the morphism $({\text {ev}}^{(p)})^{*}L_{\iota _{P^0}}[-1] \rightarrow (R\pi _{*}\mathcal {F})^\vee$. So in order to compare $\mathbf {E}^\circ$ and $(\mathrm {cut}^\circ )^{*}\mathbf {F}^\circ$, we have to compare $f^{*} T_{X_0^\circ |\mathrm {pt}_\mathbb {N}}$ and $\mathcal {F}$. The sheaf $f^* T_{X_0^\circ |\mathrm {pt}_{\mathbb {N}}}$ can be written as the kernel of

$$\begin{aligned}&f^* \bigoplus _{V \in V({\tilde{\varGamma }})} (\iota _{X_{\varDelta _V}^\circ })_* (\iota _{X_{\varDelta _V}^\circ })^* T_{X_0^\circ |\mathrm {pt}_{\mathbb {N}}} \rightarrow \bigoplus _{E \in E_f({\tilde{\varGamma }})} (\iota _{E})_* ({\text {ev}}^E)^* T_{X_0^\circ |\mathrm {pt}_{\mathbb {N}}} \\&(s_V)_V \mapsto (s_{V_E^1}|_{D_E^\circ } -s_{V_E^2}|_{D_E^\circ })_E. \end{aligned}$$

Remark that because $X_0$ is the special fiber of a toric degeneration, all the log tangent bundles $T_{X_0}$, $T_{X_{\varDelta _V}}$, $T_{D_E}$ are free sheaves (see e.g. Section 7 of [38]). In particular, the restrictions $(\iota _{X_{\varDelta _V}^\circ })^* T_{X_0^\circ |\mathrm {pt}_{\mathbb {N}}} \rightarrow T_{X_{\varDelta _V}^\circ }$ are isomorphisms, the restriction

$$\begin{aligned} \bigoplus _{E \in E_f({\tilde{\varGamma }})} ({\text {ev}}^E)^* T_{X_0^\circ |\mathrm {pt}_{\mathbb {N}}} \rightarrow \bigoplus _{E \in E_f({\tilde{\varGamma }})} ({\text {ev}}^E)^{*} T_{D_E^\circ } \end{aligned}$$

has kernel $\bigoplus _{E \in E_f({\tilde{\varGamma }})} ({\text {ev}}^E)^{*} \mathcal {O}_{D_E^\circ }$ and so there is an induced exact sequence

$$\begin{aligned} 0 \rightarrow f^* T_{X_0^\circ |\mathrm {pt}_{\mathbb {N}}} \rightarrow \mathcal {F}\rightarrow \bigoplus _{E \in E_f({\tilde{\varGamma }})} (\iota _E)_* ({\text {ev}}^E)^* \mathcal {O}_{D_E^\circ } \rightarrow 0, \end{aligned}$$

which induces an exact triangle on ${\overline{M}}_{g, n, \varDelta }^{{\tilde{h}},P^0 \circ }$:

$$\begin{aligned} (\mathrm {cut}^\circ )^* \mathbf {F}^\circ \rightarrow \mathbf {E}^\circ \rightarrow \bigoplus _{E \in E_f({\tilde{\varGamma }})} ({\text {ev}}^E)^* \mathcal {O}_{D_E^\circ }[1] \xrightarrow {[1]} . \end{aligned}$$

It remains to check the compatibility of this exact triangle with the exact triangle of cotangent complexes. We have

$$\begin{aligned} \mu ^{*} L_{\mathfrak {M}_{g,n,\varDelta }^{{\tilde{h}}}| \underset{V \in V({\tilde{\varGamma }})}{\prod } \mathfrak {M}_{g(V), \varDelta _V}} =\bigoplus _{E\in E_f({\tilde{\varGamma }})} (\iota _E)^* \mathcal {O}_{p_E}. \end{aligned}$$

Indeed, restricted to the locus of torically transverse stable log maps, $\mathrm {cut}_C$ is smooth, and, given a torically transverse stable log map to $\mathcal {X}_0/\mathrm {pt}_\mathbb {N}$, a basis of first order infinitesimal deformations fixing its image by $\mathrm {cut}_C$ in $\prod _{V \in V({\tilde{\varGamma }})} \mathfrak {M}_{g(V), \varDelta _V}$ is indexed by the cutting nodes. The dual of the natural map

$$\begin{aligned} \bigoplus _{E \in E_f({\tilde{\varGamma }})} ({\text {ev}}^E)^* \mathcal {O}_{D_E^\circ } \rightarrow \mu ^{*} L_{\mathfrak {M}_{g,n,\varDelta }^{{\tilde{h}}}| \underset{V \in V({\tilde{\varGamma }})}{\prod } \mathfrak {M}_{g(V), \varDelta _V}} =\bigoplus _{E\in E_f({\tilde{\varGamma }})} (\iota _E)^* \mathcal {O}_{p_E} \end{aligned}$$

sends the canonical first order infinitesimal deformation indexed by the cutting node $p_E$ to the canonical summand $\mathcal {O}_{D_E^\circ }$ in the normal bundle to the diagonal $\prod _{E \in E_f({\tilde{\varGamma }})} D_E^\circ$ in $\prod _{E \in E_f({\tilde{\varGamma }})} (D_E^\circ )^2$, and so is an isomorphism. This guarantees the compatibility with the exact triangle of cotangent complexes. $\square$

**Remark** Restricted to the open locus of torically transverse stable maps, the discussion is essentially reduced to a collection of gluings along the smooth divisors $D_E^\circ$. A comparison of the obstruction theories in the context of the degeneration formula along a smooth divisor is given with full details in Section 7 of [27].

#### Proposition 20

We have

$$\begin{aligned}&(\mathrm {cut}^\circ )_* \left( [{\overline{M}}_{g, n, \varDelta }^{{\tilde{h}},P^0,\circ }]^{\mathrm {virt}} \right) \\&\quad = \left( \prod _{E \in E_f({\tilde{\varGamma }})} w(E) \right) \left( (\delta \times \iota _{P^0})_M^! \prod _{V \in V({\tilde{\varGamma }})}[{\overline{M}}_{g(V), \varDelta _V}^\circ ]^{\mathrm {virt}} \right) . \end{aligned}$$

#### Proof

It follows from Proposition 19 and from Theorem 4.8 of [29] that the relative obstruction theories $\mathbf {E}^\circ$ and $(\mathrm {cut}^\circ )^* \mathbf {F}^\circ$ define the same virtual fundamental class on ${\overline{M}}_{g, n, \varDelta }^{{\tilde{h}},P^0,\circ }$. By Proposition 18, $\mathrm {cut}^\circ$ is étale, and so, by Proposition 7.2 of [7], the virtual fundamental class defined by $(\mathrm {cut}^\circ )^* \mathbf {F}^\circ$ is the image by $(\mathrm {cut}^\circ )^*$ of the virtual fundamental class defined by $\mathbf {F}^\circ$. It follows that

$$\begin{aligned}{}[{\overline{M}}_{g, n, \varDelta }^{{\tilde{h}},P^0,\circ }]^{\mathrm {virt}}= (\text {cut}^\circ )^* (\delta \times \iota _{P^0})_M^! \prod _{V \in V({\tilde{\varGamma }})}[{\overline{M}}_{g(V), \varDelta _V}^\circ ]^{\mathrm {virt}} . \end{aligned}$$

According to Proposition 18, the morphism $\mathrm {cut}^\circ$ is étale of degree

$$\begin{aligned} \prod _{E \in E_f({\tilde{\varGamma }})} w(E), \end{aligned}$$

and so the result follows from the projection formula. $\square$

### Gluing

Recall that we have the morphismFor every $V \in V({\tilde{\varGamma }})$, we have a projection morphism

$$\begin{aligned} \mathrm {pr}_V :\underset{V' \in V({\tilde{\varGamma }})}{\prod } {\overline{M}}_{g(V'), \varDelta _{V'}} \rightarrow {\overline{M}}_{g(V), \varDelta _V}. \end{aligned}$$

On each moduli space ${\overline{M}}_{g(V), \varDelta _V}$, we have the top lambda class $(-1)^{g(V)} \lambda _{g(V)}$.

#### Proposition 21

We have

$$\begin{aligned} N_{g,{\tilde{h}}}^{\varDelta ,n}=\int _{(\delta \times \iota _{P^0})^! \underset{V \in V({\tilde{\varGamma }})}{\prod } [{\overline{M}}_{g(V), \varDelta _V}]^{\mathrm {virt}}} (\delta \times \iota _{P^0})_M^{*} \prod _{V \in V({\tilde{\varGamma }})} \mathrm {pr}_V^{*} \left( (-1)^{g(V)} \lambda _{g(V)} \right) . \end{aligned}$$

#### Proof

By definition (see Sect. 4.3), we have

$$\begin{aligned} N_{g,{\tilde{h}}}^{\varDelta ,n} = \int _{[{\overline{M}}_{g,n,\varDelta }^{{\tilde{h}},P^0}]^{\mathrm {virt}}} (-1)^{g-g_{\varDelta ,n}} \lambda _{g-g_{\varDelta ,n}} . \end{aligned}$$

Using the gluing properties of lambda classes given by Lemma 7, we obtain that

$$\begin{aligned} (-1)^{g-g_{\varDelta , n}} \lambda _{g-g_{\varDelta , n}}= (\text {cut})^* (\delta \times \iota _{P^0})_M^{*} \prod _{V \in V({\tilde{\varGamma }})} \mathrm {pr}_V^{*} \left( (-1)^{g(V)} \lambda _{g(V)} \right) . \end{aligned}$$

It follows from the projection formula that

$$\begin{aligned} N_{g,{\tilde{h}}}^{\varDelta ,n} = \int _{(\text {cut})_*[{\overline{M}}_{g,n,\varDelta }^{{\tilde{h}},P^0}]^{\mathrm {virt}}} (\delta \times \iota _{P^0})_M^{*} \prod _{V \in V({\tilde{\varGamma }})} \mathrm {pr}_V^{*} \left( (-1)^{g(V)} \lambda _{g(V)} \right) . \end{aligned}$$

According to Proposition 20, the cycles

$$\begin{aligned} (\text {cut})_* \left( [{\overline{M}}_{g, n, \varDelta }^{{\tilde{h}},P^0}]^{\mathrm {virt}} \right) \end{aligned}$$

and

$$\begin{aligned} \left( \prod _{E \in E_f({\tilde{\varGamma }})} w(E) \right) \left( (\delta \times \iota _{P^0})^! \prod _{V \in V({\tilde{\varGamma }})}[{\overline{M}}_{g(V), \varDelta _V}]^{\mathrm {virt}} \right) \end{aligned}$$

have the same restriction to the open substackofIt follows, by Proposition 1.8 of [16], that their difference is rationally equivalent to a cycle supported on the closed substackIf we have

$$\begin{aligned} (f_V :C_V \rightarrow X_{\varDelta _V})_{V \in V({\tilde{\varGamma }})} \in Z, \end{aligned}$$

then at least one stable log map $f_V :C_V \rightarrow X_{\varDelta _V}$ is not torically transverse. By Lemma 17, the unbounded edges of the tropicalization of $f_V$ are contained in the rays of the fan of $X_{\varDelta _V}$. It follows that we can apply Proposition 11 to obtain that at least one of the source curves $C_V$ contains a non-trivial cycle of components. By the vanishing result of Lemma 8, this implies that

$$\begin{aligned} \int _Z (\delta \times \iota _{P^0})_M^{*} \prod _{V \in V({\tilde{\varGamma }})} \mathrm {pr}_V^{*} \left( (-1)^{g(V)} \lambda _{g(V)} \right) =0 . \end{aligned}$$

It follows that

$$\begin{aligned}&\int _{(\text {cut})_*[{\overline{M}}_{g,n,\varDelta }^{{\tilde{h}},P^0}]^{\mathrm {virt}}} (\delta \times \iota _{P^0})_M^{*} \prod _{V \in V({\tilde{\varGamma }})} \mathrm {pr}_V^{*} \left( (-1)^{g(V)} \lambda _{g(V)} \right) \\&\quad =\int _{(\delta \times \iota _{P^0})^! \underset{V \in V({\tilde{\varGamma }})}{\prod } [{\overline{M}}_{g(V), \varDelta _V}]^{\mathrm {virt}}} (\delta \times \iota _{P^0})_M^{*} \prod _{V \in V({\tilde{\varGamma }})} \mathrm {pr}_V^{*} \left( (-1)^{g(V)} \lambda _{g(V)} \right) . \end{aligned}$$

This finishes the proof of Proposition 21. $\square$

### Identifying the pieces

#### Proposition 22

We have

$$\begin{aligned}&\int _{(\delta \times \iota _{P^0})^! \underset{V \in V({\tilde{\varGamma }})}{\prod } [{\overline{M}}_{g(V), \varDelta _V}]^{\mathrm {virt}}} (\delta \times \iota _{P^0})_M^{*} \prod _{V \in V({\tilde{\varGamma }})} \mathrm {pr}_V^{*} \left( (-1)^{g(V)} \lambda _{g(V)} \right) \\&\quad =\prod _{V \in V({\tilde{\varGamma }})} N_{g(V),V}^{1,2}. \end{aligned}$$

#### Proof

Using the definitions of $\delta$ and $\iota _{P^0}$, we have

$$\begin{aligned}&\int _{(\delta \times \iota _{P^0})^! \underset{V \in V({\tilde{\varGamma }})}{\prod } [{\overline{M}}_{g(V), \varDelta _V}]^{\mathrm {virt}}} (\delta \times \iota _{P^0})_M^{*} \prod _{V \in V({\tilde{\varGamma }})} \mathrm {pr}_V^{*} \left( (-1)^{g(V)} \lambda _{g(V)} \right) \\&\quad = \int _{ \underset{V \in V({\tilde{\varGamma }})}{\prod } [{\overline{M}}_{g(V), \varDelta _V}]^{\mathrm {virt}}} ({\text {ev}}^{(p)})^{*}([P^0]) ({\text {ev}}^{(e)})^{*}([\delta ]) \prod _{V \in V({\tilde{\varGamma }})} \mathrm {pr}_V^{*} \left( (-1)^{g(V)} \lambda _{g(V)} \right) , \end{aligned}$$

where

$$\begin{aligned}{}[P^0] = \prod _{V \in V^{(2p)}({\tilde{\varGamma }})} P^0_V \in A^{*}\left( \prod _{V \in V^{(2p)}({\tilde{\varGamma }})} X_{\varDelta _V} \right) \end{aligned}$$

is the class of $P^0$ and

$$\begin{aligned}{}[\delta ] \in A^{*}\left( \prod _{E \in E_f({\tilde{\varGamma }})} (D_E)^2\right) \end{aligned}$$

is the class of the diagonal $\prod _{E \in E_f({\tilde{\varGamma }})} D_E$. As each $D_E$ is a projective line, we have

$$\begin{aligned}{}[\delta ] = \prod _{E \in E_f({\tilde{\varGamma }})} (\mathrm {pt}_E \times 1 +1 \times \mathrm {pt}_E), \end{aligned}$$

where $\mathrm {pt}_E \in A^1(D_E)$ is the class of a point.

We fix an orientation of edges of ${\tilde{\varGamma }}$ as described in Sect. 6. In particular, every trivalent vertex has two ingoing and one outgoing adjacent edges, every bivalent pointed vertex has two outgoing adjacent edges, every bivalent unpointed vertex has one ingoing and one outgoing edges. For every bounded edge *E* of ${\tilde{\varGamma }}$, we denote $V_E^s$ the source vertex of *E* and $V_E^t$ the target vertex of *E*, as defined by the orientation. Furthermore, the connected components of the complement of the bivalent pointed vertices of ${\tilde{\varGamma }}$ are trees with exactly one unbounded edge.

We argue that the effect of the insertion $({\text {ev}}^{(p)})^{*}([P^0]) ({\text {ev}}^{(e)})^{*}([\delta ])$ can be computed in terms of the combinatorics of ingoing and outgoing edges of ${\tilde{\varGamma }}$.^18^ More precisely, we claim that the only term in

$$\begin{aligned} ({\text {ev}}^{(e)})^{*}([\delta ]) = \prod _{E \in E_f({\tilde{\varGamma }})} \left( ({\text {ev}}_{V_E^s}^E)^{*} (\mathrm {pt}_E)+({\text {ev}}_{V_E^t}^E)^{*} (\mathrm {pt}_E) \right) , \end{aligned}$$

giving a non-zero contribution after multiplication by

$$\begin{aligned} \left( \prod _{V \in V^{(2p)}({\tilde{\varGamma }})} ({\text {ev}}_V^{(p)})^{*}(P^0_V) \right) \left( \prod _{V \in V({\tilde{\varGamma }})} \mathrm {pr}_{V}^{*} \left( (-1)^{g(V)} \lambda _{g(V)} \right) \right) \end{aligned}$$

and integration over $\prod _{V \in V({\tilde{\varGamma }})} [{\overline{M}}_{g(V), \varDelta _V}]^{\mathrm {virt}}$ is $\prod _{E \in E_f({\tilde{\varGamma }})} ({\text {ev}}_{V_E^t}^E)^{*} (\mathrm {pt}_E)$.

We prove this claim by induction, starting at the bivalent pointed vertices, where things are constrained by the marked points $P^0$, and propagating these constraints following the flow on ${\tilde{\varGamma }}$ defined by the orientation of edges.

Let *V* be a bivalent pointed vertex, *E* an edge adjacent to *V* and $V'$ the other vertex of *E*. The edge *E* is outgoing for *V* and ingoing for $V'$, so $V'=V_E^t$. We have in $({\text {ev}}^{(p)})^{*}([P^0]) ({\text {ev}}^{(e)})^{*}([\delta ])$ a corresponding factor

$$\begin{aligned} ({\text {ev}}_{V}^{(p)})^{*}(P_V^0) \left( ({\text {ev}}_{V}^E)^{*}(\mathrm {pt}_E) +({\text {ev}}_{V'}^E)^{*}(\mathrm {pt}_E)\right) . \end{aligned}$$

But $({\text {ev}}_{V}^{(p)})^{*}(P_V^0)({\text {ev}}_{V}^E)^{*}(\mathrm {pt}_E)(-1)^{g(V)} \lambda _{g(V)}=0$ for dimension reasons (its insertion over ${\overline{M}}_{g(V), \varDelta _V}$ defines an enumerative problem of virtual dimension $-1$) and so only the factor $({\text {ev}}_{V}^{(p)})^{*}(P_V^0) ({\text {ev}}_{V'}^E)^{*}(\mathrm {pt}_E)$ survives, which proves the initial step of the induction.

Let *E* be an outgoing edge of a trivalent vertex *V*, of ingoing edges $E^1$ and $E^2$. Let $V_E^t$ be the target vertex of *E*. By the induction hypothesis, every possibly non-vanishing term contains the insertion of $({\text {ev}}_V^{E^1})^{*}(\mathrm {pt}_{E^1}) ({\text {ev}}_V^{E^2})^{*}(\mathrm {pt}_{E^2})$. But $({\text {ev}}_V^{E^1})^{*}(\mathrm {pt}_{E^1}) ({\text {ev}}_V^{E^2})^{*}(\mathrm {pt}_{E^2}) ({\text {ev}}_V^{E})^{*}(\mathrm {pt}_E) (-1)^{g(V)} \lambda _{g(V)}=0$ for dimension reasons (its insertion over ${\overline{M}}_{g(V), \varDelta _V}$ defines an enumerative problem of virtual dimension $-1$) and so only the factor $({\text {ev}}_V^{E^1})^{*}(\mathrm {pt}_E^1) ({\text {ev}}_V^{E^2})^{*}(\mathrm {pt}_E^2) ({\text {ev}}_{V_E^t}^{E})^{*}(\mathrm {pt}_E)$ survives.

Let *E* be an outgoing edge of a bivalent unpointed vertex *V*, of ingoing edges $E^1$. Let $V_E^t$ the target vertex of *E*. By the induction hypothesis, every possibly non-vanishing term contains the insertion of $({\text {ev}}_V^{E^1})^{*}(\mathrm {pt}_{E^1})$. But $({\text {ev}}_V^{E^1})^{*}(\mathrm {pt}_{E^1}) ({\text {ev}}_V^{E})^{*}(\mathrm {pt}_E) (-1)^{g(V)} \lambda _{g(V)}=0$ for dimension reasons (its insertion over ${\overline{M}}_{g(V), \varDelta _V}$ defines an enumerative problem of virtual dimension $-1$) and so only the factor $({\text {ev}}_V^{E^1})^{*}(\mathrm {pt}_{E^1}) ({\text {ev}}_{V_E^t}^{E})^{*}(\mathrm {pt}_E)$ survives. This finishes the proof by induction of the claim.

Using the notations introduced in Sect. 6, we can rewrite

$$\begin{aligned} \prod _{E \in E_f\left( {\tilde{\varGamma }}\right) } \left( {\text {ev}}_{V_E^t}^E\right) ^{*} (\mathrm {pt}_E) \end{aligned}$$

as

$$\begin{aligned} \left( \prod _{V \in V^{(3)}({\tilde{\varGamma }})} ({\text {ev}}_V^{E_V^{\mathrm {in},1}})^{*} (\mathrm {pt}_{E_V^{\mathrm {in},1}}) ({\text {ev}}_V^{E_V^{\mathrm {in},2}})^{*} (\mathrm {pt}_{E_V^{\mathrm {in},2}}) \right) \left( \prod _{V \in V^{(2up)}({\tilde{\varGamma }})} ({\text {ev}}_V^{E_V^{\mathrm {in}}})^{*} (\mathrm {pt}_{E_V^{\mathrm {in}}}) \right) \end{aligned}$$

and so we proved

$$\begin{aligned}&\int _{(\delta \times \iota _{P^0})^! \underset{V \in V({\tilde{\varGamma }})}{\prod } [{\overline{M}}_{g(V), \varDelta _V}]^{\mathrm {virt}}} (\delta \times \iota _{P^0})_M^{*} \prod _{V \in V({\tilde{\varGamma }})} \mathrm {pr}_V^{*} \left( (-1)^{g(V)} \lambda _{g(V)} \right) \\&\quad = \left( \prod _{V \in V^{(3)}({\tilde{\varGamma }})} N^{1,2}_{g(V),V} \right) \left( \prod _{V \in V^{(2p)}({\tilde{\varGamma }})} N^{1,2}_{g(V),V} \right) \left( \prod _{V \in V^{(2up)}({\tilde{\varGamma }})} N^{1,2}_{g(V),V} \right) . \end{aligned}$$

This finishes the proof of Proposition 22. $\square$

### End of the proof of the gluing formula

The gluing identity given by Proposition 13 follows from the combination of Proposition 21 and Proposition 22.

## Vertex contribution

In this Section, we evaluate the invariants $N_{g,V}^{1,2}$ attached to the vertices *V* of $\varGamma$ and appearing in the gluing formula of Corollary 16. The first step, carried out in Sect. 8.1 is to rewrite these invariants in terms of more symmetric invariants $N_{g,V}$ depending only on the multiplicity of the vertex *V*. In Sect. 8.2, we use the consistency of the gluing formula to deduce non-trivial relations between these invariants and to reduce the question to the computation of the invariants attached to vertices of multiplicity one and two. Invariants attached to vertices of multiplicity one and two are explicitly computed in Sect. 8.3 and this finishes the proof of Theorem 1. Modifications needed to prove Theorem 6 are discussed at the end of Sect. 8.4.

### Reduction to a function of the multiplicity

The gluing formula of the previous Section, Corollary 16, expresses the log Gromov–Witten invariant $N^{\varDelta , n}_{g,h}$ attached to a parametrized tropical curve $h :\varGamma \rightarrow \mathbb {R}^2$ as a product of log Gromov–Witten $N^{1,2}_{g(V),V}$ attached to the trivalent vertices *V* of $\varGamma$, and of the weights *w*(*E*) of the edges *E* of $\varGamma$. The definition of $N^{1,2}_{g(V),V}$ given in Sect. 6 depends on a specific choice of orientation on the edges of $\varGamma$. In particular, the definition of $N^{1,2}_{g(V),V}$ does not treat the three edges adjacent to *V* in a symmetric way.

Let $E_V^{\text {in},1}$ and $E_V^{\text {in},2}$ be the two ingoing edges adjacent to *V*, and let $E_V^{\text {out}}$ be the outgoing edge adjacent to *V*. Let $D_{E_V^{\text {in},1}}$, $D_{E_V^{\text {in},2}}$ and $D_{E_V^{\text {out}}}$ be the corresponding toric divisors of $X_{\varDelta _V}$. We have evaluation morphisms

$$\begin{aligned} \text {ev} = (\text {ev}_1, \text {ev}_2, \text {ev}_{\text {out}}) :{\overline{M}}_{g,\varDelta _V} \rightarrow D_{E_V^{\text {in},1}} \times D_{E_V^{\text {in},2}} \times D_{E_V^{\text {out}}} . \end{aligned}$$

In Sect. 6, we defined

$$\begin{aligned} N_{g,V}^{1, 2} = \int _{[{\overline{M}}_{g,\varDelta _V}]^{\mathrm {virt}}}(-1)^g \lambda _g \text {ev}_1^* (\mathrm {pt}_1) \text {ev}_2^* (\mathrm {pt}_2) , \end{aligned}$$

where $\mathrm {pt}_1 \in A^1 (D_{E_V^{\text {in},1}})$ and $\mathrm {pt}_2 \in A^1(D_{E_V^{\text {in},2}})$ are classes of a point on $D_{E_V^{\text {in},1}}$ and $D_{E_V^{\text {in},2}}$ respectively.

But one could similarly define

$$\begin{aligned} N_{g,V}^{2 , \text {out}} \,{:}{=}\, \int _{[{\overline{M}}_{g,\varDelta _V}]^{\mathrm {virt}}}(-1)^g \lambda _g \text {ev}_2^* (\mathrm {pt}_2) \text {ev}_{\text {out}}^* (\mathrm {pt}_{\text {out}}) , \end{aligned}$$

and

$$\begin{aligned} N_{g,V}^{\text {out}, 1} \,{:}{=}\, \int _{[{\overline{M}}_{g,\varDelta _V}]^{\mathrm {virt}}}(-1)^g \lambda _g \text {ev}_{\text {out}}^* (\mathrm {pt}_{\text {out}}) \text {ev}_1^* (\mathrm {pt}_1) , \end{aligned}$$

where $\mathrm {pt}_{\text {out}} \in A^* (D_{E_V^{\text {out}}})$ is the class of a point on $E_V^{\text {out}}$. The following Lemma gives a relation between these various invariants.

#### Lemma 23

We have

$$\begin{aligned} N_{g,V}^{1, 2} w(E_V^{\mathrm {in},1}) w(E_V^{\mathrm {in},2}) = N_{g,V}^{2 , \mathrm {out}} w(E_V^{\mathrm {in},2})w(E_V^{\mathrm {out}}) = N_{g,V}^{\mathrm {out}, 1} w(E_V^{\mathrm {out}}) w(E_V^{\mathrm {in},1}) \end{aligned}$$

and we denote by $N_{g,V}$ this number.

#### Proof

Let $\varGamma _V$ be the trivalent tropical curve given by *V* and its three edges $E_V^{\text {in},1}$, $E_V^{\text {in},2}$ and $E_V^{\text {out}}$. Let $\varGamma _{V'}$ be the trivalent tropical curve with a unique vertex $V'$ and edges $E_{V'}^{\text {in},1}$, $E_{V'}^{\text {in},2}$ and $E_{V'}^{\text {out}}$, such that

$$\begin{aligned} w(E_V^{\text {in},1})=w(E_{V'}^{\text {in},1}) , w(E_V^{\text {in},2})=w(E_{V'}^{\text {in},2}), w(E_V^{\text {out}})=w(E_{V'}^{\text {out}}), \end{aligned}$$

and

$$\begin{aligned} v_{V,E_V^{\text {in},1}}=-v_{V',E_{V'}^{\text {in},1}} , v_{V, E_V^{\text {in},2}}=-v_{V',E_{V'}^{\text {in},2}}, v_{V, E_V^{\text {out}}}=-v_{V', E_{V'}^{\text {out}}}. \end{aligned}$$

Let $\varGamma _{V,V'}$ be the tropical curve obtained by gluing $E_V^{\text {out}}$ and $E_{V'}^{\text {out}}$ together.

Taking

$$\begin{aligned} \varDelta = \left\{ v_{V,E_V^{\text {in},1}}, -v_{V',E_{V'}^{\text {in},1}}, v_{V, E_V^{\text {in},2}},-v_{V',E_{V'}^{\text {in},2}} \right\} \end{aligned}$$

and $n=3$, we have $g_{\varDelta , n}=0$ and $T_{\varDelta , p}$ consists of a unique tropical curve $\varGamma _{V,V'}^p$, obtained from $\varGamma _{V,V'}$ by adding three bivalent vertices corresponding to the three point $p_1$, $p_2$ and $p_3$ in $\mathbb {R}^2$.

Choosing differently $p=(p_1, p_2, p_3)$, the tropical curve $\varGamma _{V,V'}^p$ can look like 
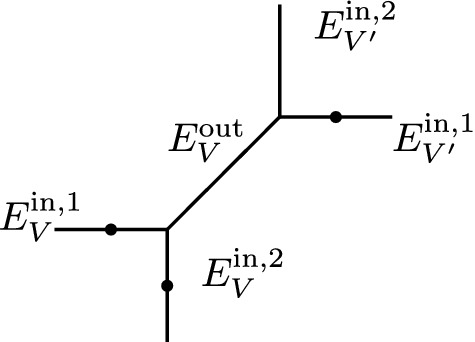


or like 
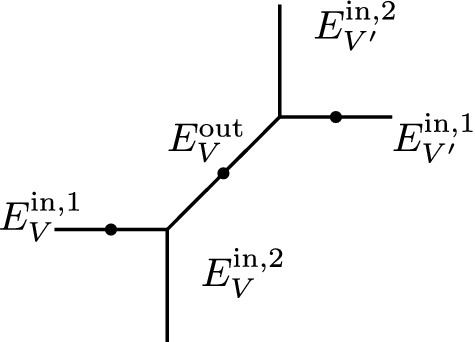


But the log Gromov–Witten invariants $N^{\varDelta , 3}_g$ are independent of the choice of *p* and so can be computed for any choice of *p*. For each of the two above choices of *p*, the gluing formula of Corollary 16 gives an expression for $N^{\varDelta , 3}_g$. These two expressions have to be equal. Writing

$$\begin{aligned} F(u) = \sum _{g \geqslant 0} N_g u^{2g+1} \, \end{aligned}$$

we obtain^19^

$$\begin{aligned}&F^{1,2}_V(u) F^{1, \text {out}}_{V'}(u) w(E_{V}^{\text {in},1}) w(E_{V}^{\text {in},2}) w(E_{V}^{\text {out}}) w(E_{V'}^{\text {in,1}})\\&\quad = F^{1, \text {out}}_{V}(u) F^{1, \text {out}}_{V'}(u) w(E_{V}^{\text {in},1}) w(E_V^{\text {out}}) w(E_V^{\text {out}}) w(E_{V'}^{\text {in},1}), \end{aligned}$$

and so after simplification

$$\begin{aligned} F^{1,2}_V(u) F^{1, \text {out}}_{V'}(u) w(E_{V}^{\text {in},2}) = F^{1, \text {out}}_{V}(u) F^{1, \text {out}}_{V'}(u)w(E_V^{\text {out}}). \end{aligned}$$

By $GL_2(\mathbb {Z})$ invariance, we have $F^{1,2}_V(u)=F^{1,2}_{V'}(u)$ and $F^{1, \text {out}}_{V}(u)=F^{1, \text {out}}_{V'}(u)$. By the unrefined correspondence theorem, we know that $F^{1, \text {out}}_{V}(u) \ne 0$, so we obtain

$$\begin{aligned} F^{1,2}_V(u) w(E_{V}^{\text {in},2}) = F^{1, \text {out}}_{V}(u) w(E_V^{\text {out}}), \end{aligned}$$

which finishes the proof of Lemma 23. $\square$

We define the contribution $F_V(u) \in \mathbb {Q}[\![u]\!]$ of a trivalent vertex *V* of $\varGamma$ as being the power series

$$\begin{aligned} F_V(u)= \sum _{g \geqslant 0} N_{g,V} u^{2g+1}. \end{aligned}$$

#### Proposition 24

For every $\varDelta$ and *n* such that $g_{\varDelta , n} \geqslant 0$, and for every $p \in U_{\varDelta , n}$, we have

$$\begin{aligned} \sum _{g \geqslant g_{\varDelta ,n}} N_g^{\varDelta , n} u^{2g-2+|\varDelta |} = \sum _{(h :\varGamma \rightarrow \mathbb {R}^2) \in T_{\varDelta , p}} \prod _{V \in V^{(3)}(\varGamma )} F_V(u) \, \end{aligned}$$

where the product is over the trivalent vertices of $\varGamma$.

#### Proof

This follows from the decomposition formula, Proposition 10, from the gluing formula, Corollary 16, and from Lemma 23. Indeed, every bounded edge of $\varGamma$ is an ingoing edge for exactly one trivalent vertex of $\varGamma$ and every trivalent vertex of $\varGamma$ has exactly two ingoing edges. Combining the invariant $N^{1,2}_{g(V),V}$ of a trivalent vertex *V* with the weights of its two ingoing edges, one can rewrite the double product of Corollary 16 as a single product in terms of the invariants defined by Lemma 23. $\square$

#### Proposition 25

The contribution $F_V(u)$ of a vertex *V* only depends on the multiplicity *m*(*V*) of *V*.

In particular, for every *m* positive integer, one can define the contribution $F_m(u) \in \mathbb {Q}[\![u]\!]$ as the contribution $F_V(u)$ of a vertex *V* of multiplicity *m*.

#### Proof

We follow closely Brett Parker, [42] (Section 3).

For $v_1, v_2 \in \mathbb {Z}^2-\{0\}$, let us denote by $F_{v_1, v_2}(u)$ the contribution $F_V(u)$ of a vertex *V* of adjacent edges $E_1$, $E_2$ and $E_3$ such that $v_{V,E_1}=v_1$ and $v_{V,E_2}=v_2$. The contribution $F_{v_1, v_2}(u)$ depends on $(v_1, v_2)$ only up to linear action of $GL_2(\mathbb {Z})$ on $\mathbb {Z}^2$. In particular, we can change the sign of $v_1$ and/or $v_2$ without changing $F_{v_1, v_2}(u)$.

By the balancing condition, we have $v_{V,E_3}=-v_{V,E_1}-v_{V,E_2}$ and so

$$\begin{aligned} F_{v_1, v_2}(u) = F_{-v_1, v_2}(u) =F_{v_1-v_2, v_2}(u). \end{aligned}$$

By $GL_2(\mathbb {Z})$ invariance, we can assume $v_1=(|v_1|,0)$ and $v_2=(v_{2x},*)$ with $v_{2x} \geqslant 0$. If $|v_1|$ divides $v_{2x}$, $v_{2x}=a|v_1|$, then replacing $v_2$ by $v_2-av_1$, which does not change $F_{v_1,v_2}$, we can assume that $v_1=(|v_1|,0)$ and $v_2=(0, *)$. If not, we do the Euclidean division of $v_{2x}$ by $|v_1|$, $v_{2x}=a|v_1|+b$, $0 \leqslant b<|v_1|$, and we replace $v_2$ by $v_2-av_1$ to obtain $v_2=(b,*)$. Exchanging the roles of $v_1$ and $v_2$, we can assume by $GL_2(\mathbb {Z})$ invariance that $v_1=(|v_1|,0)$, for some $|v_1| \leqslant b$ and $v_2=(v_{2x},*)$ for some $v_{2x}\geqslant 0$, and we repeat the above procedure. By the Euclidean algorithm, this process terminates and at the end we have $v_1=(|v_1|,0)$ and $v_2=(0,|v_2|)$. In particular, for every $v_1, v_2 \in \mathbb {Z}^2-\{0\}$, the contribution $F_{v_1,v_2}$ only depends on $\text {gcd}(|v_1|, |v_2|)$ and on the multiplicity $|\det (v_1, v_2)|$.

By the previous paragraph, we can assume that $v_1=(|v_1|,0)$ and $v_2=(0,|v_2|)$.

Taking

$$\begin{aligned} \varDelta = \{ (|v_1|,0), (0,|v_2|), (0,1), (-|v_1|, -|v_2|-1) \}, \end{aligned}$$

and $n=3$, we have $g_{\varDelta , n} =0$ and $T_{\varDelta , p}$ contains a unique tropical curve $\varGamma ^p$.

Choosing differently $p=(p_1, p_2, p_3)$, the tropical curve $\varGamma ^p$ can look like 
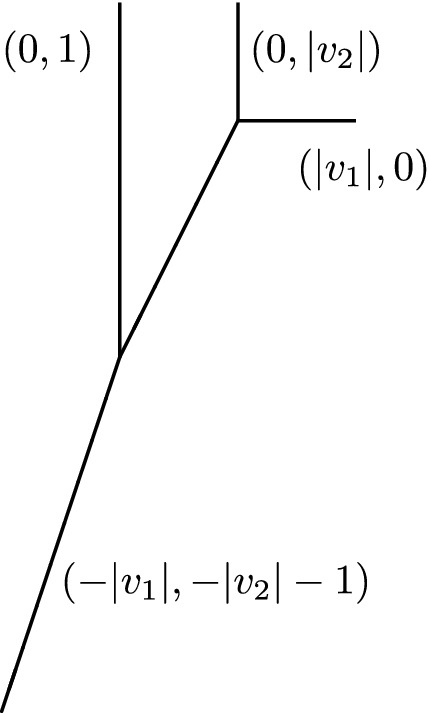


or like 
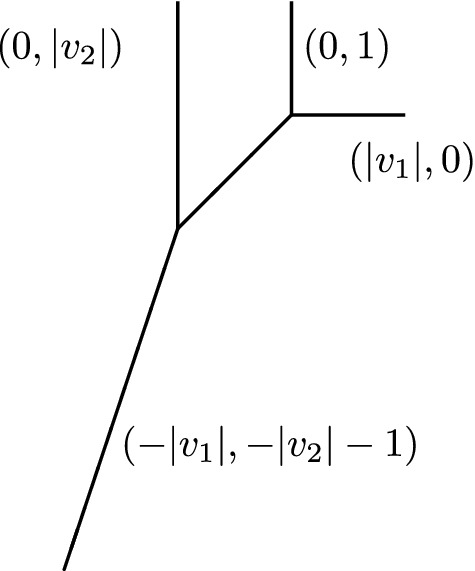


But the log Gromov–Witten invariants $N^{\varDelta , 3}_g$ are independent of the choice of *p* and so can be computed for any choice of *p*. For each of the two above choices of *p*, the gluing formula of Proposition 24 gives an expression for $N^{\varDelta , 3}_g$. These two expressions have to be equal and we obtain

$$\begin{aligned}&F_{(|v_1|,0), (0,|v_2|)}(u) F_{(0,1), (-|v_1|,-|v_2|-1)}(u)\\&\quad =F_{(|v_1|,0),(0,1)}(u) F_{(0,|v_2|),(-|v_1|, -|v_2|-1)}(u) . \end{aligned}$$

For both pairs of vectors $(|v_1|,0), (0,1)$ and $(0,1), (-|v_1|,-|v_2|-1)$, the gcd of the divisibilities is equal to one and the absolute value of the determinant is equal to $|v_1|$, so we have

$$\begin{aligned} F_{(0,1), (-|v_1|,-|v_2|-1)}(u) = F_{(|v_1|,0),(0,1)}(u). \end{aligned}$$

As this quantity is non-zero by the unrefined correspondence theorem, we can simplify it from the previous equality to obtain

$$\begin{aligned} F_{(|v_1|,0),(0, |v_2|)}(u)= F_{(0,|v_2|),(-|v_1|, -|v_2|-1)}(u) . \end{aligned}$$

As

$$\begin{aligned} \text {gcd}(|(0,|v_2|)|, |(-|v_1|, -|v_2|-1)|)=1, \end{aligned}$$

we obtain the desired result. $\square$

### Reduction to vertices of multiplicity 1 and 2

We start reviewing the key step in the argument of Itenberg and Mikhalkin [25] proving the tropical deformation invariance of Block-Göttsche invariants. We consider a tropical curve with a 4-valent vertex *V*. Let *Q* be the quadrilateral dual to *V*. We assume that *Q* has no pair of parallel sides. In that case, there exists a unique parallelogram *P* having two sides in common with *Q* and being contained in *Q*. Let *A*,*B*,*C* and *D* denote the four vertices of *Q*, such that *A*,*B* and *D* are vertices of *P*. Let *E* be the fourth vertex of *P*, contained in the interior of *Q*. There are three combinatorially distinct ways to deform this tropical curve into a simple one, corresponding to the three ways to decompose *Q* into triangles or parallelograms:

1. We can decompose *Q* into the triangles *ABD* and *BCD*.
2. We can decompose *Q* into the triangles *ABC* and *ACD*.
3. We can decompose *Q* into the triangles *BCE*, *DEC* and the parallelogram *P*.

Case (1):

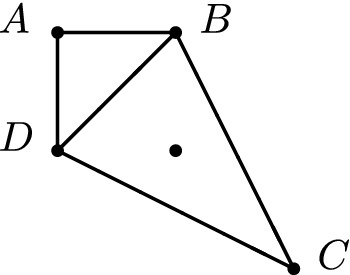



Case (2):

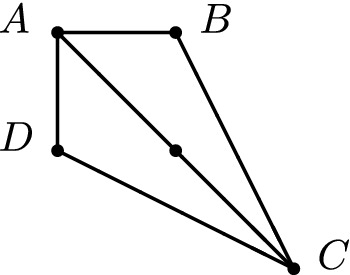



Case (3):

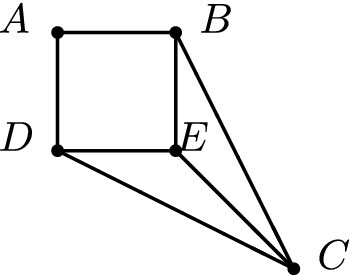



The deformation invariance result then follows from the identity

$$\begin{aligned}&(q^{|ACD|}-q^{-|ACD|}) (q^{|ABC|}-q^{-|ABC|})\\&\quad =(q^{|BCD|}-q^{-|BCD|}) (q^{|ABD|}-q^{-|ABD|})\\&\qquad +(q^{|BCE|}-q^{-|BCE|}) (q^{|DEC|}-q^{-|DEC|}), \end{aligned}$$

where $|-|$ denotes the area. This identity can be proved by elementary geometry considerations.

The following result goes in the opposite direction and shows that the constraints imposed by tropical deformation invariance are quite strong. The generating series of log Gromov–Witten invariants $F_m(u)$ will satisfy these constraints. Indeed, they are defined independently of any tropical limit, so applications of the gluing formula to different degenerations have to give the same result.

#### Proposition 26

Let $F :\mathbb {Z}_{>0} \rightarrow R$ be a function of positive integers valued in a commutative ring *R*, such that, for any quadrilateral *Q* as above, we have^20^

$$\begin{aligned} F(2|BCD|)F(2|ABD|)= & {} F(2|ACD|)F(2|ABC|)\\&+ F(2|BCE|)F(2|DEC|). \end{aligned}$$

Then for every integer $n \geqslant 2$, we have

$$\begin{aligned} F(n)^2=F(2n-1)F(1)+F(n-1)^2 \end{aligned}$$

and for every integer $n \geqslant 3$, we have

$$\begin{aligned} F(n)^2=F(2n-2)F(2)+F(n-2)^2. \end{aligned}$$

In particular, if *F*(1) and *F*(2) are invertible in *R*, then the function *F* is completely determined by its values *F*(1) and *F*(2).

#### Proof

The first equality is obtained by taking *Q* to be the quadrilateral of vertices $(-1,0)$, $(-1,1)$, (0, 1), $(n-1, -(n-1))$.

Picture of *Q* for $n=2$: 
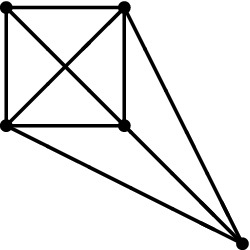


The second equality is obtained by taking *Q* to be the quadrilateral of vertices $(-1,0)$, $(-1,1)$, (1, 0), $(n-1,-(n-1))$.

Picture of *Q* for $n=3$: 
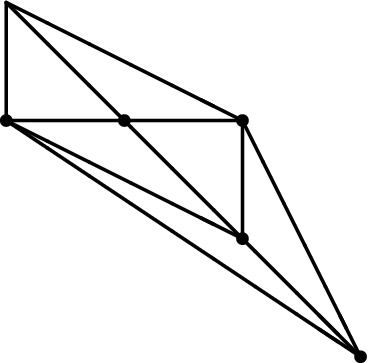
$$\square $$

### Contribution of vertices of multiplicity 1 and 2

#### Vertex of multiplicity one

We now evaluate the contribution $F_1(u)$ of a vertex of multiplicity 1 by direct computation.

We consider $\varDelta = \{(-1,0), (0,-1), (1,1)\}$. The corresponding toric surface $X_\varDelta$ is simply $\mathbb {P}^{2}$, of fan

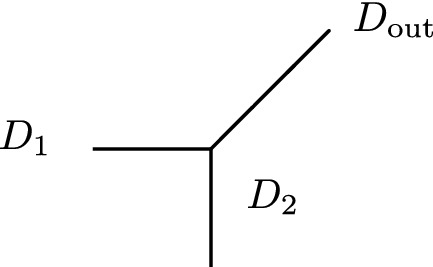



and of dual polygon

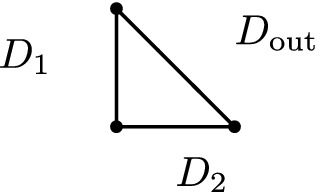



Let $D_1$, $D_2$ and $D_{\text {out}}$ be the toric boundary divisors of $\mathbb {P}^2$. The class $\beta _{\varDelta }$ is simply the class of a curve of degree one, i.e. of a line, on $\mathbb {P}^2$. Let ${\overline{M}}_{g,\varDelta }$ be the moduli space of genus *g* stable log maps of type $\varDelta$. We have evaluation maps

$$\begin{aligned} (\text {ev}_1, \text {ev}_2) :{\overline{M}}_{g,\varDelta } \rightarrow D_1 \times D_2 , \end{aligned}$$

and in Sect. 6, we defined

$$\begin{aligned} N_{g,\varDelta }^{1, 2} = \int _{[{\overline{M}}_{g,\varDelta _V}]^{\mathrm {virt}}}(-1)^g \lambda _g \text {ev}_1^* (\mathrm {pt}_1) \text {ev}_2^* (\mathrm {pt}_2) , \end{aligned}$$

where $\mathrm {pt}_1 \in A^* (D_1)$ and $\mathrm {pt}_2 \in A^*(D_2)$ are classes of a point on $D_1$ and $D_2$ respectively.

By definition (see Sect. 8.1), we have

$$\begin{aligned} F_1(u)= \sum _{g \geqslant 0} N_{g,\varDelta }^{1, 2} u^{2g+1}. \end{aligned}$$

##### Proposition 27

The contribution of a vertex of multiplicity one is given by

$$\begin{aligned} F_1(u) = 2\sin \left( \frac{u}{2} \right) = -i\left( q^{\frac{1}{2}}-q^{-\frac{1}{2}}\right) \end{aligned}$$

where $$q=e^{iu}$$.

##### Proof

Let $P_1$ and $P_2$ be points on $D_1$ and $D_2$ respectively, away from the torus fixed points. Let *S* be the surface obtained by blowing-up $\mathbb {P}^2$ at $P_1$ and $P_2$. Denote by *D* the strict transform of the class of a line in $\mathbb {P}^2$ and by $E_1$, $E_2$ the exceptional divisors. Denote $\partial S$ the strict transform of the toric boundary $\partial \mathbb {P}^2$ of $\mathbb {P}^2$. We endow *S* with the divisorial log structure with respect to $\partial S$. Let ${\overline{M}}_g(S)$ be the moduli space of genus *g* stable log maps to *S* of class $D-E_1-E_2$ with tangency condition to intersect $\partial S$ in one point with multiplicity one. It has virtual dimension *g* and we define

$$\begin{aligned} N_g^S \,{:}{=}\, \int _{[{\overline{M}}_g(S)]^{\mathrm {virt}}} (-1)^g \lambda _g . \end{aligned}$$

The strict transform *C* of the line *L* in $\mathbb {P}^2$ passing through $P_1$ and $P_2$ is the unique genus zero curve satisfying these conditions and has normal bundle $N_{C|S}=\mathcal {O}_{\mathbb {P}^1}(-1)$ in *S*. All the higher genus maps factor through *C*, and as *C* is away from the preimage of the torus fixed points of $\mathbb {P}^2$, log invariants coincide with relative invariants [5]. More precisely, we can consider the moduli space ${\overline{M}}_g(\mathbb {P}^1/\infty , 1,1)$ genus *g* stable maps to $\mathbb {P}^1$, of degree one, and relative to a point $\infty \in \mathbb {P}^1$. If $\pi :\mathcal {C}\rightarrow {\overline{M}}_g(\mathbb {P}^1/\infty , 1,1)$ is the universal curve and $f :\mathcal {C}\rightarrow \mathbb {P}^1 \simeq C$ is the universal map, the difference in obstruction theories between stable maps to *S* and stable maps to $\mathbb {P}^1$ comes from $R^1 \pi _* f^* N_{C|S}=R^1 \pi _* f^* \mathcal {O}_{\mathbb {P}^1}(-1)$. So we obtain

$$\begin{aligned} N_g^S= \int _{[{\overline{M}}_g(\mathbb {P}^1/\infty , 1,1)]^{\mathrm {virt}}}(-1)^g \lambda _g \, e \left( R^1 \pi _* f^{*} \mathcal {O}_{\mathbb {P}^1} (-1) \right) , \end{aligned}$$

where $e(-)$ is the Euler class. Rewriting

$$\begin{aligned} (-1)^g \lambda _g = e(R^1 \pi _* \mathcal {O}_{\mathcal {C}}) =e(R^1 \pi _* f^* \mathcal {O}_{\mathbb {P}^1}), \end{aligned}$$

we get

$$\begin{aligned} N_g^S= \int _{[{\overline{M}}_g(\mathbb {P}^1/\infty , 1,1)]^{\mathrm {virt}}} e \left( R^1 \pi _* f^* (\mathcal {O}_{\mathbb {P}^1} \oplus \mathcal {O}_{\mathbb {P}^1} (-1)) \right) . \end{aligned}$$

These integrals have been computed by Bryan and Pandharipande[12], (see the proof of the Theorem 5.1), and the result is

$$\begin{aligned} \sum _{g \geqslant 0} N_g^S u^{2g-1} = \frac{1}{2 \sin \left( \frac{u}{2} \right) } . \end{aligned}$$

As in [23], we will work with the non-compact varieties $(\mathbb {P}^2)^\circ$, $D_1^\circ$, $D_2^\circ$, $S^\circ$ obtained by removing the torus fixed points of $\mathbb {P}^2$ and their preimages in *S*.

Denote $\mathbb {P}_1^\circ$ the projectivized normal bundle to $D_1^\circ$ in $(\mathbb {P}^2)^\circ$, coming with two natural sections $(D_1^\circ )_0$ and $(D_1^\circ )_\infty$. Denote ${\tilde{\mathbb {P}}}_1^\circ$ the blow-up of $\mathbb {P}_1^\circ$ at the point $P_1 \in (D_1^\circ )_\infty$, $\tilde{E}_1$ the corresponding exceptional divisor and $C_1$ the strict transform of the fiber of $\mathbb {P}_1^\circ$ passing through $P_1$. In particular, $\tilde{E}_1$ and $C_1$ are both projective lines with degree $-1$ normal bundle in $({\tilde{\mathbb {P}}}_1)^\circ$. Furthermore, $\tilde{E}_1$ and $C_1$ intersect in one point. Similarly, denote $\mathbb {P}_2^\circ$ the projectivized normal bundle to $D_2^\circ$ in $(\mathbb {P}^2)^\circ$, coming with two natural sections $(D_2^\circ )_0$ and $(D_2^\circ )_\infty$. Denote ${\tilde{\mathbb {P}}}_2^\circ$ the blow-up of $\mathbb {P}_2^\circ$ at the point $P_2 \in (D_2^\circ )_\infty$, $\tilde{E}_2$ the corresponding exceptional divisor and $C_2$ the strict transform of the fiber of $\mathbb {P}_2^\circ$ passing through $P_2$. In particular, $\tilde{E}_2$ and $C_2$ are both projective lines with degree $-1$ normal bundle in $({\tilde{\mathbb {P}}}_2)^\circ$. Furthermore, $\tilde{E}_2$ and $C_2$ intersect in one point.

We degenerate $S^\circ$ as in Section 5.3 of [23]. We first degenerate $(\mathbb {P}^2)^\circ$ to the normal cone of $D_1^\circ \cup D_2^\circ$, i.e. we blow-up $(D_1^\circ \cup D_2^\circ ) \times \{0\}$ in $(\mathbb {P}^2)^{\circ } \times \mathbb {C}$. The fiber over $0 \in \mathbb {C}$ has three irreducible components: $(\mathbb {P}^2)^\circ$, $\mathbb {P}_1^\circ$, $\mathbb {P}_2^\circ$, with $\mathbb {P}_1^\circ$ and $\mathbb {P}_2^\circ$ glued along $(D_1^\circ )_0$ and $(D_2^\circ )_0$ to $D_1^\circ$ and $D_2^\circ$ in $(\mathbb {P}^2)^\circ$. We then blow-up the strict transforms of the sections $P_1 \times \mathbb {C}$ and $P_2 \times \mathbb {C}$. The fiber of the resulting family away from $0 \in \mathbb {C}$ is isomorphic to $S^\circ$. The fiber over zero has three irreducible components: $(\mathbb {P}^2)^{\circ }$, ${\tilde{\mathbb {P}}}_1^{\circ }$, ${\tilde{\mathbb {P}}}_2^{\circ }$.

We would like to apply a degeneration formula to this family in order to compute $N_g^S$. As discussed above, all the maps in ${\overline{M}}_g(S)$ factor through *C* and so $N_g^S$ can be seen as a relative Gromov–Witten invariant of the non-compact surface $S^\circ$, relatively to the strict transforms of $D_1^\circ$ and $D_2^\circ$.

The key point is that for homological degree reasons, the degenerating relative stable maps do not leave the non-compact geometries we are considering. More precisely, any limiting relative stable map has to factor through $C_1 \cup L \cup C_2$, with degree one over each of the components $C_1$, *L* and $C_2$. So, even if the target geometry is non-compact, all the relevant moduli spaces of relative stable maps are compact. It follows that we can apply the ordinary degeneration formula in relative Gromov–Witten theory [28].

We obtain

$$\begin{aligned} \sum _{g \geqslant 0} N_g^S u^{2g-1} = \left( \sum _{g \geqslant 0} N_{g, \varDelta }^{1, 2} u^{2g+1} \right) \left( \sum _{g \geqslant 0} N_g^{C_1} u^{2g-1} \right) \left( \sum _{g \geqslant 0} N_g^{C_2} u^{2g-1} \right) . \end{aligned}$$

The invariants $N_g^{C_1}$ and $N_g^{C_2}$, coming from curves factoring through $C_1$ and $C_2$, which are $(-1)$-curves in ${\tilde{\mathbb {P}}}_1^\circ$ and ${\tilde{\mathbb {P}}}_2^\circ$ respectively, can be written as relative invariants of $\mathbb {P}^1$:

$$\begin{aligned} N_g^{C_1}=N_g^{C_2}= \int _{[{\overline{M}}_g(\mathbb {P}^1/\infty , 1,1)]^{\mathrm {virt}}} e \left( R^1 \pi _* f^* (\mathcal {O}_{\mathbb {P}^1} \oplus \mathcal {O}_{\mathbb {P}^1} (-1)) \right) , \end{aligned}$$

which is exactly the formula giving $N_g^S$, and so

$$\begin{aligned} \sum _{g \geqslant 0} N_g^{C_1} u^{2g-1} =\sum _{g \geqslant 0} N_g^{C_2} u^{2g-1} =\frac{1}{2 \sin \left( \frac{u}{2} \right) } . \end{aligned}$$

Remark that this equality is a higher genus version of Proposition 5.2 of [23]. Combining the previous equalities, we obtain

$$\begin{aligned} \frac{1}{2 \sin \left( \frac{u}{2} \right) } = \left( \sum _{g \geqslant 0} N_{g, \varDelta }^{1, 2} u^{2g+1} \right) \left( \frac{1}{2 \sin \left( \frac{u}{2} \right) } \right) ^2 , \end{aligned}$$

and so

$$\begin{aligned} \sum _{g \geqslant 0} N_{g, \varDelta }^{1, 2} u^{2g+1} = 2 \sin \left( \frac{u}{2} \right) . \end{aligned}$$

$$\square $$

#### Vertex of multiplicity 2

We now evaluate the contribution $F_2(u)$ of a vertex of multiplicity 2 by direct computation.

We consider $\varDelta =\{(-1,0), (0,-2), (1,2)\}$. The corresponding toric surface $X_\varDelta$ is simply the weighted projective plane $\mathbb {P}^{1,1,2}$, of fan

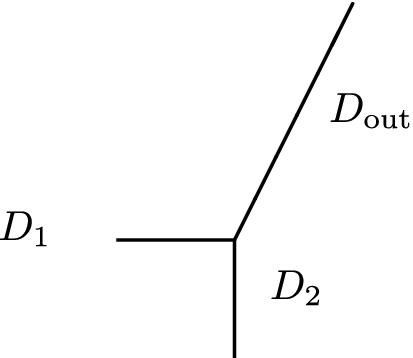



and of dual polygon

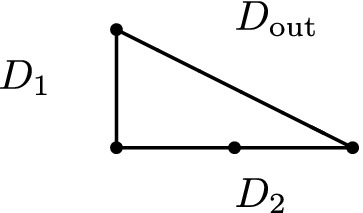



Let $D_1$, $D_2$ and $D_{\text {out}}$ be the toric boundary divisors of $\mathbb {P}^{1,1,2}$. We have the following numerical properties:

$$\begin{aligned}&2D_1=D_2=2D_{\text {out}},\\&D_1.D_2=1,\, D_1.D_{\text {out}}=\frac{1}{2},\, D_2.D_{\text {out}}=1,\\&D_1^2=\frac{1}{2}, D_2^2=2, D_{\text {out}}^2=\frac{1}{2}. \end{aligned}$$

The class $\beta _\varDelta$ satisfies $\beta _\varDelta .D_1=1$, $\beta _\varDelta .D_2=2$, $\beta _\varDelta .D_{\text {out}}=1$ and so

$$\begin{aligned} \beta _\varDelta = 2D_1=D_2=2D_{\text {out}}. \end{aligned}$$

Let ${\overline{M}}_{g,\varDelta }$ be the moduli space of genus *g* stable log maps of type $\varDelta$. We have evaluation maps

$$\begin{aligned} (\text {ev}_1, \text {ev}_2) :{\overline{M}}_{g,\varDelta } \rightarrow D_1 \times D_2 , \end{aligned}$$

and in Sect. 6, we defined

$$\begin{aligned} N_{g,\varDelta }^{1, 2} = \int _{[{\overline{M}}_{g,\varDelta _V}]^{\mathrm {virt}}}(-1)^g \lambda _g \text {ev}_1^* (\mathrm {pt}_1) \text {ev}_2^* (\mathrm {pt}_2) , \end{aligned}$$

where $\mathrm {pt}_1 \in A^* (D_1)$ and $\mathrm {pt}_2 \in A^*(D_2)$ are classes of a point on $D_1$ and $D_2$ respectively.

By definition (see Sect. 8.1), we have

$$\begin{aligned} F_1(u)= 2 \left( \sum _{g \geqslant 0} N_{g,\varDelta }^{1, 2} u^{2g+1} \right) . \end{aligned}$$

##### Proposition 28

The contribution of a vertex of multiplicity two is given by

$$\begin{aligned} F_2(u)= 2 \sin (u) = (-i) \left( q-q^{-1}\right) \end{aligned}$$

where $$q=e^{iu}$$.

##### Proof

We have to prove that

$$\begin{aligned} \sum _{g \geqslant 0} N_{g,\varDelta }^{1, 2} u^{2g+1} = \sin (u) . \end{aligned}$$

Let $P_2$ be a point on $D_2$ away from the torus fixed points. Let *S* be the surface obtained by blowing-up $\mathbb {P}^{1,1,2}$ at $P_2$. Still denote $\beta _\varDelta$ the strict transform of the class $\beta _\varDelta$ and by $E_2$ the exceptional divisor. Denote $\partial S$ the strict transform of the toric boundary $\partial \mathbb {P}^{1,1,2}$ of $\mathbb {P}^{1,1,2}$. We endow *S* with the divisorial log structure with respect to $\partial S$. Let ${\overline{M}}_g(S)$ be the moduli space of genus *g* stable log maps to *S* of class $\beta _\varDelta -2E_2$ with tangency condition to intersect $D_1$ in one point with multiplicity one and $D_{\text {out}}$ in one point with multiplicity one. It has virtual dimension *g* and we have an evaluation map

$$\begin{aligned} \text {ev}_1 :{\overline{M}}_{g,S} \rightarrow D_1 \, \end{aligned}$$

We define

$$\begin{aligned} N_g^S \,{:}{=}\, \int _{[{\overline{M}}_g(S)]^{\mathrm {virt}}} (-1)^g \lambda _g \text {ev}_1^*(\mathrm {pt}_1), \end{aligned}$$

where $\mathrm {pt}_1 \in A^1(D_1)$ is the class of a point on $D_1$.

In fact, because a curve in the linear system $\beta _\varDelta -2E_2$ is of arithmetic genus $g_a$ given by

$$\begin{aligned} 2g_a-2&=(\beta _\varDelta -2E_2) \cdot (\beta _\varDelta -2E_2+K_S) \\&= (2D_1-2E_2) \cdot (2D_1-4D_1-E_2) \\&=-4D_1^2+2E_2^2\\&=-4 , \end{aligned}$$

i.e. $g_a=-1<0$, all the moduli spaces ${\overline{M}}_g(S)$ are empty and so

$$\begin{aligned} \sum _{g \geqslant 0} N_g^S u^{2g-1}=0 . \end{aligned}$$

We write $\tilde{\varDelta } =\{(-1,0), (0,-1), (0,-1), (1,2)\}$ and ${\overline{M}}_{g,\tilde{\varDelta }}$ the moduli space of genus *g* stable log maps of type $\tilde{\varDelta }$. We have evaluation maps

$$\begin{aligned} (\text {ev}_1, \text {ev}_2, \text {ev}_{2'}) :{\overline{M}}_{g,\tilde{\varDelta }} \rightarrow D_1 \times D_2 \times D_2 , \end{aligned}$$

and we define

$$\begin{aligned} N_{g,\tilde{\varDelta }}^{1,2,2'} \,{:}{=}\, \int _{[{\overline{M}}_{g,\tilde{\varDelta }}]^{\mathrm {virt}}} (-1)^g \lambda _g \text {ev}_1^*(\mathrm {pt}_1) \text {ev}_2^*(\mathrm {pt}_2) \text {ev}_{2'}^*(\mathrm {pt}_2) , \end{aligned}$$

where $\mathrm {pt}_1 \in A^* (D_1)$ and $\mathrm {pt}_2 \in A^*(D_2)$ are classes of a point on $D_1$ and $D_2$ respectively.

As in [23], we will work with the non-compact varieties $(\mathbb {P}^{1,1,2})^\circ$, $D_1^\circ$, $D_2^\circ$, $S^\circ$ obtained by removing the torus fixed points of $\mathbb {P}^{1,1,2}$ and their preimages in *S*. Denote $\mathbb {P}_2^\circ$ the projectivized normal bundle to $D_2^\circ$ in $(\mathbb {P}^2)^\circ$, coming with two natural sections $(D_2^\circ )_0$ and $(D_2^\circ )_\infty$. Denote ${\tilde{\mathbb {P}}}_2^\circ$ the blow-up of $\mathbb {P}_2^\circ$ at the point $P_2 \in (D_2^\circ )_\infty$, $\tilde{E}_2$ the corresponding exceptional divisor and $C_2$ the strict transform of the fiber of $\mathbb {P}_2^\circ$ passing through $P_2$. In particular, $\tilde{E}_2$ and $C_2$ are both projective lines with degree $-1$ normal bundle in $({\tilde{\mathbb {P}}}_2)^\circ$. Furthermore, $\tilde{E}_2$ and $C_2$ intersect in one point.

We degenerate $S^\circ$ as in Section 5.3 of [23]. We first degenerate $(\mathbb {P}^{1,1,2})^\circ$ to the normal cone of $D_2^\circ$, i.e. we blow-up $D_2^\circ \times \{0\}$ in $(\mathbb {P}^{1,1,2})^{\circ } \times \mathbb {C}$. The fiber over $0 \in \mathbb {C}$ has two components: $(\mathbb {P}^{1,1,2})^\circ$ and $\mathbb {P}_2^\circ$, with $\mathbb {P}_2^\circ$ glued along $(D_2^\circ )_0$ to $D_2^\circ$ in $(\mathbb {P}^{1,1,2})^\circ$. We then blow-up the strict transform of the section $P_2 \times \mathbb {C}$. The fiber of the resulting family away from $0 \in \mathbb {C}$ is isomorphic to $S^\circ$. The fiber over zero has two components: $(\mathbb {P}^{1,1,2})^{\circ }$ and ${\tilde{\mathbb {P}}}_2^{\circ }$.

We would like to apply a degeneration formula to this family in order to compute $N_g^S$. The key point is that for homological degree reasons, the relevant degenerating relative stable maps do not leave the non-compact geometries we are considering. More precisely, after fixing a point $P_1 \in D_1^\circ$, realizing the insertion ${\text {ev}}_1^{*}(\mathrm {pt}_1)$, any limiting relative stable map has to factor through $L \cup C_2$, with degree one over *L* and degree two over $C_2$, where *L* is the unique curve in $\mathbb {P}^{1,1,2}$, of class $\beta _{\varDelta }$, passing through $P_1$ and through $P_2$ with tangency order two along $D_2^{\circ }$. So, even if the target geometry is non-compact, all the relevant moduli spaces of relative stable maps are compact. It follows that we can apply the ordinary degeneration formula in relative Gromov–Witten theory [28].

The application of the degeneration formula gives two terms, corresponding to the two partitions $2=1+1$ and $2=2$ of the intersection number

$$\begin{aligned} (\beta _\varDelta -2E_2).E_2=2. \end{aligned}$$

For the first term, the invariants on the side of $\mathbb {P}^{1,1,2}$ are $N_{g,\tilde{\varDelta }}^{1,2,2'}$, whereas on the side of ${\tilde{\mathbb {P}}}_2$, we have disconnected invariants, corresponding to two degree one maps to $C_2$. As in the proof of Proposition 27, the relevant connected degree one invariants of $C_2$ are given by

$$\begin{aligned} N_g^{C_2}= \int _{[{\overline{M}}_g(\mathbb {P}^1/\infty , 1,1)]^{\mathrm {virt}}} e \left( R^1 \pi _* f^* (\mathcal {O}_{\mathbb {P}^1} \oplus \mathcal {O}_{\mathbb {P}^1} (-1)) \right) , \end{aligned}$$

satisfying

$$\begin{aligned} \sum _{g \geqslant 0} N_g^{C_2} u^{2g-1} =\frac{1}{2 \sin \left( \frac{u}{2} \right) } . \end{aligned}$$

For the second term, the invariants on the side of $\mathbb {P}^{1,1,2}$ are $N_{g,\varDelta }^{1,2}$, whereas on the side of ${\tilde{\mathbb {P}}}_2$, we have connected invariants, corresponding to one degree two map to $C_2$. More precisely, the relevant connected degree two invariants of $C_2$ are given by

$$\begin{aligned} N_g^{2C_2}= \int _{[{\overline{M}}_g(\mathbb {P}^1/\infty , 2,2)]^{\mathrm {virt}}} e \left( R^1 \pi _* f^* (\mathcal {O}_{\mathbb {P}^1} \oplus \mathcal {O}_{\mathbb {P}^1} (-1)) \right) , \end{aligned}$$

where ${\overline{M}}_g(\mathbb {P}^1/\infty , 2,2)$ is the moduli space of genus *g* stable maps to $\mathbb {P}^1$, of degree two, and relative to a point $\infty \in \mathbb {P}^1$ with maximal tangency order two. According to [12] (see the proof of Theorem 5.1), we have

$$\begin{aligned} \sum _{g \geqslant 0} N_g^{2C_2} u^{2g-1} =-\frac{1}{2} \frac{1}{2 \sin (u)}. \end{aligned}$$

It follows that the degeneration formula takes the form

$$\begin{aligned} \sum _{g \geqslant 0} N_g^S u^{2g-1}= & {} \frac{1}{2} \left( \sum _{g \geqslant 0} N_{g,\tilde{\varDelta }}^{1,2,2'} u^{2g+2} \right) \left( \frac{1}{2 \sin \left( \frac{u}{2} \right) } \right) ^2 \\&+2 \left( \sum _{g \geqslant 0} N_{g, \varDelta }^{1,2} u^{2g+1} \right) \frac{(-1)}{2} \frac{1}{2 \sin (u)} . \end{aligned}$$

The factor $\frac{1}{2}$ in front of the fist term is a symmetry factor and the factor 2 in front of the second term is a multiplicity.

There exists a unique tropical curve of type $\tilde{\varDelta }$, which looks like 
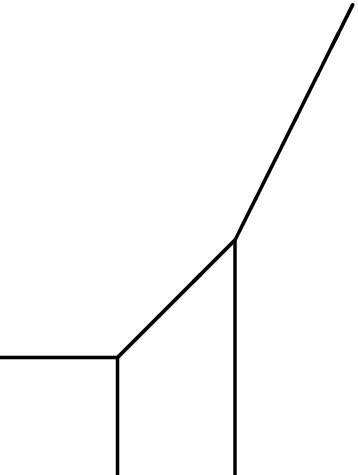


This tropical curve has two vertices of multiplicity one, so using the gluing formula of Proposition 24 and Proposition 27, we find

$$\begin{aligned} \sum _{g \geqslant 0} N_{g,\tilde{\varDelta }}^{1,2,2'} u^{2g+2}=(F_1(u))^2 = \left( 2 \sin \left( \frac{u}{2} \right) \right) ^2 . \end{aligned}$$

Combining the previous results, we obtain

$$\begin{aligned} 0 = \frac{1}{2} - \frac{1}{2 \sin (u)} \left( \sum _{g \geqslant 0} N_{g, \varDelta }^{1,2} u^{2g+1} \right) , \end{aligned}$$

and so the desired formula. $\square$

**Remark** The proofs of Propositions 27 and 28 rely on the fact that the involved curves have low degree. More precisely, in each case, the key point is that the dual polygon does not contain any interior integral point, i.e. a generic curve in the corresponding linear system on the surface has genus zero. This implies that, after imposing tangency constraints, all the higher genus stable maps factor through some rigid genus zero curve in the surface. This guarantees the compactness result needed to work as we did with relative Gromov–Witten theory of non-compact geometries. The higher genus generalization of the most general case of the degeneration argument of Section 5.3 of [23] cannot be dealt with in the same way. This generalization will be treated and applied in [9], using techniques similar to those used to prove the gluing formula in Sect. 7.

### Contribution of a general vertex

#### Proposition 29

The contribution of a vertex of multiplicity *m* is given by

$$\begin{aligned} F_m(u)=(-i) \left( q^{\frac{m}{2}}-q^{-\frac{m}{2}}\right) . \end{aligned}$$

#### Proof

By Proposition 27, the result is true for $m=1$ and by Proposition 28, the result is true for $m=2$. By consistency of the gluing formula of Proposition 24, the function $F(m) \,{:}{=}\, F_m$ valued in the ring $R \,{:}{=}\, \mathbb {Q}[\![u]\!]$ satisfies the hypotheses of Proposition 26. The result follows by induction on *m* using Proposition 26. $\square$

The proof of Theorem 1 (Theorem 5 in Sect. 2.5) follows from the combination of Propositions 24, 25 and 29.

To prove Theorem 6, generalizing Theorem 1 by allowing to fix the positions of some of the intersection points with the toric boundary, we only have to organize the gluing procedure slightly differently. The connected components of the complement of the bivalent vertices of $\varGamma$, as at the beginning of Sect. 6, are trees with one unfixed unbounded edge and possibly several fixed unbounded edges. We fix an orientation of the edges such that edges adjacent to bivalent pointed vertices go out of the bivalent pointed vertices, such that the fixed unbounded edges are ingoing and such that the unfixed unbounded edge is outgoing. With respect to this orientation, every trivalent vertex has two ingoing and one outgoing edges, and so, without any modification, we obtain the analogue of the gluing formula of Corollary 16:

$$\begin{aligned} N_{g, h}^{\varDelta , n} = \left( \prod _{V \in V^{(3)}(\varGamma )} N_{g(V), V}^{1, 2} \right) \left( \prod _{E \in E_f(\varGamma )} w(E) \right) . \end{aligned}$$

In Lemma 23, we defined $N_{g,V} \,{:}{=}\, N_{g(V), V}^{1, 2} w(E_V^{\text {in},1}) w(E_V^{\text {in},2})$, where $E_V^{\text {in},1}$ and $E_V^{\text {in},1}$ are the ingoing edges adjacent to *V*. Every bounded edge is an ingoing edge to some vertex but some ingoing edges are fixed unbounded edges and so

$$\begin{aligned} N_{g, h}^{\varDelta , n} =\left( \prod _{E_\infty ^F \in E_\infty ^F(\varGamma )} \frac{1}{w(E_\infty ^F)} \right) \left( \prod _{V \in V^{(3)}(\varGamma )} N_{g(V), V} \right) , \end{aligned}$$

where the first product is over the fixed unbounded edges of $\varGamma$. Theorem 6 then follows from Proposition 29.

## A An explicit example

In this appendix, we check by a direct computation one of the consequences of Theorem 1. Let us consider the problem of counting rational cubic curves in $\mathbb {P}^2$ passing through 8 points in general position. To match the notations of the Introduction, we choose $\varDelta$ containing three times the vector (1, 0), three times the vector (0, 1) and three times the vector $(-1,-1)$.

The toric surface $X_\varDelta$ is then $\mathbb {P}^2$ and the curve class $\beta _\varDelta$ is the class of a cubic curve in $\mathbb {P}^2$. We have $|\varDelta |=9$, $n=8$, $g_{\varDelta ,n}=0$. Let us write $N_g$ for $N_g^{\varDelta ,n}$. We have $N_0=12$ and the corresponding Block-Göttsche invariant is $q+10+q^{-1}$ (see Example 1.3 of [37] for pictures of tropical curves). From the point of view of Göttsche–Shende [19], the relevant relative Hilbert scheme to consider happens to be the pencil of cubics passing through the 8 given points, i.e. $\mathbb {P}^2$ blown-up in 9 points, whose Hirzebruch genus is indeed $1+10q+q^2$.

According to Theorem 5, we have

$$\begin{aligned} \sum _{g \geqslant 0} N_g u^{2g-2+9}= & {} i(q+10+q^{-1}) (q^{\frac{1}{2}}-q^{-\frac{1}{2}})^7\\= & {} i(q^{\frac{9}{2}} +3q^{\frac{7}{2}} -48q^{\frac{5}{2}} +168q^{\frac{3}{2}} -294q^{\frac{1}{2}} +294q^{-\frac{1}{2}}\\&\quad -\,168q^{-\frac{3}{2}} +48q^{-\frac{5}{2}} -3q^{-\frac{7}{2}} -q^{-\frac{9}{2}})\\= & {} 12 u^7 - \frac{9}{2}u^9+\frac{137}{160}u^{11} -\frac{1253}{11520}u^{13}+\cdots \end{aligned}$$

We will check directly that $N_1=-\frac{9}{2}$. Remark that a Block-Göttsche invariant equal to 12 rather than to $q+10+q^{-1}$ would lead to $N_1=-\frac{7}{2}$. In particular, the value of $N_1$ is already sensitive to the choice of the correct refinement.

We have^21^

$$\begin{aligned} N_1=\int _{[{\overline{M}}_{1,8}(\mathbb {P}^2,3)]^{\text {virt}}} (-1)^1 \lambda _1 \prod _{j=1}^8 \text {ev}_j^{*}(\mathrm {pt}), \end{aligned}$$

where $\text {pt} \in A^2(\mathbb {P}^2)$ is the class of a point. Introducing an extra marked point and using the divisor equation, one can write

$$\begin{aligned} N_1=\frac{1}{3}\int _{[{\overline{M}}_{1,8+1}(\mathbb {P}^2,3)]^{\text {virt}}} (-1)^1 \lambda _1 \left( \prod _{j=1}^8 \text {ev}_j^{*}(\mathrm {pt}) \right) \text {ev}_9^{*}(h) , \end{aligned}$$

where $h \in A^1(\mathbb {P}^2)$ is the class of a line. On ${\overline{M}}_{1,1}$, we have

$$\begin{aligned} \lambda _1 = \frac{1}{12} \delta _0 , \end{aligned}$$

where $\delta _0$ is the class of a point. Taking for representative of $\delta _0$ the point corresponding to the nodal genus one curve, with *j*-invariant $i\infty$, and resolving the node, we can write

$$\begin{aligned} N_1\!=\!-\frac{1}{12}\cdot \frac{1}{2}\cdot \frac{1}{3} \int _{[{\overline{M}}_{0, 8+1+2}(\mathbb {P}^2,3)]^{\mathrm {virt}}} \left( \prod _{j=1}^8 \text {ev}_j^{*}(\mathrm {pt}) \right) \text {ev}_9^{*}(h) (\text {ev}_{10}^{*} \times \text {ev}_{11}^{*})(D), \end{aligned}$$

where the factor $\frac{1}{2}$ comes from the two ways of labeling the two points resolving the node, and *D* is the class of the diagonal in $\mathbb {P}^2 \times \mathbb {P}^2$. We have

$$\begin{aligned} D=1 \times \mathrm {pt} + \mathrm {pt} \times 1 + h \times h. \end{aligned}$$

The first two terms do not contribute to $N_1$ for dimension reasons so

$$\begin{aligned} N_1=-\frac{1}{12}\cdot \frac{1}{2}\cdot \frac{1}{3} \int _{[{\overline{M}}_{0, 8+1+2}(\mathbb {P}^2,3)]^{\mathrm {virt}}} \left( \prod _{j=1}^8 \text {ev}_j^{*}(\mathrm {pt}) \right) \text {ev}_9^{*}(h) \text {ev}_{10}^{*}(h)\text {ev}_{11}^{*}(h). \end{aligned}$$

Using the divisor equation, we obtain

$$\begin{aligned} N_1=-\frac{1}{12}\cdot \frac{1}{2}\cdot 3 \cdot 3 \int _{[{\overline{M}}_{0, 8}(\mathbb {P}^2,3)]^{\mathrm {virt}}} \left( \prod _{j=1}^8 \text {ev}_j^{*}(\mathrm {pt}) \right) = -\frac{9}{24} N_0 = - \frac{9}{2} , \end{aligned}$$

as expected.

## List of symbols

*i*: The standard square root of $-1$ in $\mathbb {C}$
*q*: The formal refined variable in Block-Göttsche invariants
*u*: A formal variable keeping track of the genus in generating series of Gromov–Witten invariants, related to *q* by $q=e^{iu}$
$$A_{*}$$: A Chow group
$A^*$: An operatorial cohomology Chow group, see [16]
$\varDelta$: A balanced collection of vectors in $\mathbb {Z}^2$, of cardinality $|\varDelta |$
$X_\varDelta$: The toric surface defined by $\varDelta$
$\beta _\varDelta$: The curve class defined by $\varDelta$
*n*: A number of points in $(\mathbb {C}^{*})^2$ or $\mathbb {R}^2$
$$g_{\varDelta , n}$$: The integer $$n+1-|\varDelta |$$
*P*: A set of *n* points $P_j$ in $(\mathbb {C}^{*})^2$
*p*: A set of *n* points $p_j$ in $\mathbb {R}^2$
$\varGamma$: A graph, often source of a parametrized tropical curve
$V(\varGamma )$: The set of vertices of $\varGamma$, of cardinality $|V(\varGamma )|$
$E(\varGamma )$: The set of edges of $\varGamma$, of cardinality $|E(\varGamma )|$
$E_f(\varGamma )$: The set of bounded edges of $\varGamma$, of cardinality $|E_f(\varGamma )|$
$E_\infty (\varGamma )$: The set of bounded edges of $\varGamma$, of cardinality $|E_\infty (\varGamma )|$
*h*: A parametrized tropical curve
*m*(*V*): The multiplicity of a vertex *V*
*w*(*E*): The weight of an edge *E*
${\overline{M}}_{g, n, \varDelta }$: A moduli space of genus *g* stable log maps
$N_g^{\varDelta , n}$: A genus *g* log Gromov–Witten invariant
$N_{\mathrm {trop}}^{\varDelta , n}(q)$: A refined tropical curve count
$T_{\varDelta , p}$: A finite set of genus $g_{\varDelta , n}$ parametrized tropical curves
$T^g_{\varDelta , p}$: A finite set of genus *g* parametrized tropical curves
$$\overline{\mathcal {M}}$$: A monoid
$\mathrm {pt}_{\overline{\mathcal {M}}}$: The log point of ghost monoid $\overline{\mathcal {M}}$
$\varSigma$: The tropicalization functor
$X_0$: The central fiber of a toric degeneration of $X_\varDelta$
$P^0$: A set of *n* points $P^0_j$ in $X_0$, degeneration of *P*
$X_{\varDelta _V}$: An irreducible component of $X_0$
$N_{g,h}^{\varDelta , n}$: A genus *g* log Gromov–Witten invariant marked by *h*
$N_{g,V}^{1,2}$: A genus *g* log Gromov–Witten invariant attached to a vertex *V* with a preferred choice of edges
$N_{g,V}$: A genus *g* log Gromov–Witten invariant attached to a vertex *V*
$F_V(u)$: A generating series of log Gromov–Witten invariants attached to a vertex *V*
$F_m(u)$: A generating series of log Gromov–Witten invariants attached to a vertex of multiplicity *m*

## Footnotes

1 Precise definitions are given in Sect. 2.

2 And was for example presented at the Workshop: Curves on surfaces and threefold, EPFL, Lausanne, 21 June 2016.

3 A given element of $\mathbb {Z}^2 -\{0\}$ can appear several times in $\varDelta$. Here we follow the notation used by Itenberg and Mikhalkin in [25].

4 This is true only if the elements in $\varDelta$ are not all collinear. If they are, we replace $X_\varDelta$ by a toric compactification whose choice will be irrelevant for our purposes.

5 Moduli spaces of stable log maps have a natural structure of log stack. The structure of log stack is particularly important to treat correctly evaluation morphisms in log Gromov–Witten theory in general, see [1]. In this paper, we always consider these moduli spaces as stacks over the category of schemes, not as log stacks, and we will always work with naive evaluation morphisms between stacks, not log stacks. This will be enough for us. See the remark at the end of Sect. 4.2 for some justification.

6 The dualizing line bundle of a nodal curve coincides with the log cotangent bundle up to some twist by marked points and so is a completely natural object from the point of view of log geometry.

7 Here, the rigidity assumption is only necessary to forbid contracted edges. It happens to be the natural assumption in the general form of the decomposition formula of [3], as explained and used in Sect. 4.3.

8 All the monoids considered will be commutative and with an identity element.

9 As already mentioned in Sect. 2.2, we consider moduli spaces of stable log maps as stacks, not log stacks. In particular, the morphisms $\text {ev}$, $\iota _{P^0}$ and the fiber product defining ${\overline{M}}_{g,n,\varDelta }(X_0 / \mathrm {pt}_{\mathbb {N}}, P^0)$ are defined in the category of stacks, not log stacks.

10 I thank the referee for stressing this point.

11 In Section 6.3.2 of [3], sections defining point constraints have to interact non-trivially with the log structure of the special fiber to produce something interesting because the degeneration considered there is a trivial product, whereas we are considering a non-trivial degeneration.

12 In [3], the marking includes also a choice of curve classes for the stable maps $f_V$. In our case, the curve classes are uniquely determined because a curve class in a toric variety is uniquely determined by its intersection numbers with the components of the toric boundary divisor.

13 We allow a torically transverse stable log map to have components contracted to points of $\partial X_\varDelta$ which are not torus fixed points. In particular, we use a notion of torically transverse map which is slightly different from the one used by Nishinou and Siebert in [38].

14 This result was expected: see Remark 3.4 of [22] but it seems that no proof was published until now.

15 As in Sect. 2.2, 1-pointed means that the source curves are equipped with one marked point in addition to the marked points keeping track of the tangency conditions.

16 We are considering a stable log map over a point. It is a notational exercise to extend the argument to a stable log map over a general base, which is required to really define a morphism between moduli spaces.

17 The base monoid of a basic stable log map has always such description in terms of deformations of tropical curves. See Remark 1.18 and Remark 1.21 of [24] for more details.

18 It is essentially a cohomological reformulation and generalization of the way the gluing is organized in Mikhalkin’s proof of the tropical correspondence theorem, [34].

19 Recall that we are considering marked points as bivalent vertices and that this affects the notion of bounded edge. According to the gluing formula of Corollary 16, we need to include one weight factor for each bounded edge.

20 All the relevant areas are half-integers and so their doubles are indeed integers.
